# Next-generation DNA sequencing-based assay for measuring allelic expression imbalance (AEI) of candidate neuropsychiatric disorder genes in human brain

**DOI:** 10.1186/1471-2164-12-518

**Published:** 2011-10-20

**Authors:** Xiang Xu, Hao Wang, Min Zhu, Yue Sun, Yu Tao, Qin He, Jian Wang, Li Chen, David Saffen

**Affiliations:** 1 Institutes of Brain Science, Fudan University, 138 Yixueyuan Road, Shanghai 200032, China; 2 Key Laboratory for Medical Neurobiology, Fudan University, 138 Yixueyuan Road, Shanghai 200032, China; 3 School of Biological Sciences, Fudan University, 220 Handan Road, Shanghai 200433, China

## Abstract

**Background:**

Common genetic variants that regulate gene expression are widely suspected to contribute to the etiology and phenotypic variability of complex diseases. Although high-throughput, microarray-based assays have been developed to measure differences in mRNA expression among independent samples, these assays often lack the sensitivity to detect rare mRNAs and the reproducibility to quantify small changes in mRNA expression. By contrast, PCR-based allelic expression imbalance (AEI) assays, which use a "marker" single nucleotide polymorphism (mSNP) in the mRNA to distinguish expression from pairs of genetic alleles in individual samples, have high sensitivity and accuracy, allowing differences in mRNA expression greater than 1.2-fold to be quantified with high reproducibility. In this paper, we describe the use of an efficient PCR/next-generation DNA sequencing-based assay to analyze allele-specific differences in mRNA expression for candidate neuropsychiatric disorder genes in human brain.

**Results:**

Using our assay, we successfully analyzed AEI for 70 candidate neuropsychiatric disorder genes in 52 independent human brain samples. Among these genes, 62/70 (89%) showed AEI ratios greater than 1 ± 0.2 in at least one sample and 8/70 (11%) showed no AEI. Arranging log_2_AEI ratios in increasing order from negative-to-positive values revealed highly reproducible distributions of log_2_AEI ratios that are distinct for each gene/marker SNP combination. Mathematical modeling suggests that these log_2_AEI distributions can provide important clues concerning the number, location and contributions of *cis*-acting regulatory variants to mRNA expression.

**Conclusions:**

We have developed a highly sensitive and reproducible method for quantifying AEI of mRNA expressed in human brain. Importantly, this assay allowed quantification of differential mRNA expression for many candidate disease genes entirely missed in previously published microarray-based studies of mRNA expression in human brain. Given the ability of next-generation sequencing technology to generate large numbers of independent sequencing reads, our method should be suitable for analyzing from 100- to 200-candidate genes in 100 samples in a single experiment. We believe that this is the appropriate scale for investigating variation in mRNA expression for defined sets candidate disorder genes, allowing, for example, comprehensive coverage of genes that function within biological pathways implicated in specific disorders. The combination of AEI measurements and mathematical modeling described in this study can assist in identifying SNPs that correlate with mRNA expression. Alleles of these SNPs (individually or as sets) that accurately predict high- or low-mRNA expression should be useful as markers in genetic association studies aimed at linking candidate genes to specific neuropsychiatric disorders.

## Background

Neuropsychiatric disorders are complex diseases that are strongly influenced by genetic, epigenetic, and environmental factors [1]. One of the central challenges in current psychiatric research is to determine the contributions of these factors to major neuropsychiatric disorders and use this knowledge to develop effective strategies for disease diagnosis, treatment and prevention.

Beginning with the elucidation of a consensus DNA sequence of the human genome and extending through current efforts to exhaustively document genetic variability in human populations, much has been learned about the genes and genetic variants that contribute to major neuropsychiatric disorders, such as schizophrenia [2,3], Alzheimer's disease [4,5] and drug addiction [6,7]. Unlike Mendelian disorders, however, which can often be traced to mutations that disrupt gene structure or coding sequences, genetic markers that associate with complex disorders often map to chromosomal sites located outside of gene coding regions [8]. Such observations suggest that genetic variants that regulate gene expression, rather than disrupt gene structure, may be a major source of liability for, or protection from, complex disorders [8,9].

Because of their potential importance for explaining the etiology and phenotypic diversity of complex diseases, there is currently great interest in developing methods for identifying regulatory genetic variants [10,11]. Unlike coding region mutations or chromosomal rearrangements, which can be identified by DNA sequencing alone, detection of regulatory variants requires experimentation, such as measurements of variation in mRNA expression or splicing. To meet this challenge, hybridization-based microarray assays have been developed that are capable of measuring variation in the expression of hundreds-to-thousands of genes in multiple samples in a single experiment [12,13].

While microarrays have provided important information concerning variation in mRNA expression in a variety of tissues and cell lines and have the potential to identify both *cis*- and *trans*-acting regulatory variants [14,15], they are not necessarily the best choice for analyzing mRNA expression in human brain. When used to compare mRNA expression among independent samples, microarray-based assays typically require large numbers of samples to obtain statistically significant correlations between genetic variants and mRNA expression and, in the absence of a cDNA PCR amplification step, often lack the sensitivity to detect rare mRNAs [16-18]. The requirement for large numbers of samples to attain statistical significance is closely related to the large variation in mRNA expression among samples that is determined by non-genetic factors. This is particularly a problem for studies of mRNA expression in human brain, since the quality of mRNA isolated from autopsy brain tissue often varies among samples and the individuals that provided the samples differ in ages, sex, medical history, drug use and cause of death. Thus, variation in mRNA expression caused by regulatory genetic variants is often obscured, especially in small collections of unmatched samples.

In contrast to assays that involve comparisons between independent samples, assays that measure allele-specific differences in mRNA expression have the advantage that the relative level of mRNA expression from each genetic allele is determined within individual samples, with each autosomal allele serving as a control for the other [19-21]. Combined with PCR-based-amplification of cDNA reverse-transcribed from mRNA, this approach has produced highly accurate measurements of differential mRNA expression in a variety of human tissues, including brain, and provided important information about *cis*-acting genetic variants that regulate mRNA expression [22-34]. To date, however, only assays of low-throughput design have been used for allele-specific measurements of mRNA expression in human brain.

In this paper we describe a medium-throughput method for assaying allele-specific mRNA expression based on PCR amplification and next-generation DNA sequencing technology. Our results show that this assay produces allelic expression imbalance (AEI) ratios (defined as the ratio of the amount of mRNA derived from one genetic allele, divided by the amount of mRNA derived from the other allele) for mRNAs expressed in human brain of outstanding quality and reproducibility.

After measuring AEI ratios for many candidate genes, we noticed that graphs of log_2_AEI ratios ordered from most-negative to most-positive produced distribution patterns that are characteristic for each gene/marker SNP pair. Mathematical modeling of these log_2_AEI distributions revealed that they can be a rich source of information concerning the genetic variants that regulate mRNA expression for each gene. We believe that, used together, our AEI assay and mathematical modeling provide powerful tools for identifying regulatory genetic variants that contribute to major neuropsychiatric disorders.

## Results

### Next generation DNA sequencing-based AEI assay

**Figure 1:**
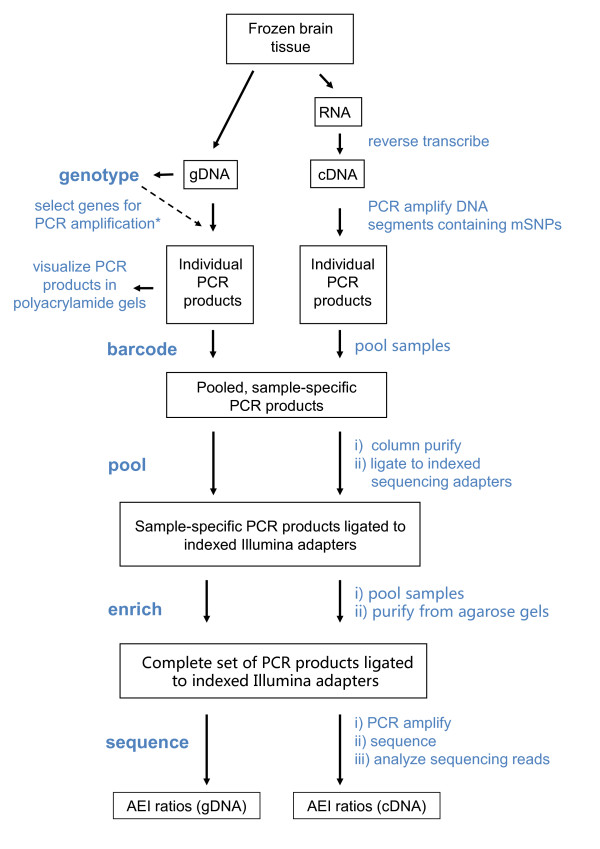
**Flow diagram for AEI assays**. Technical details are provided in Methods and Table 1 in the text and Additional file 1, Figures S1 - S5 and Additional file 2, Tables S1-S6.

An outline of our AEI assay is shown in Figure 1. Experimental details are provided in Methods, Table 1, Additional file 1, Figures S1 - S6, and Additional file 2, Tables S1 - S6. Briefly, genomic DNA (gDNA) isolated from small (~30 mg) samples of frozen human brain was used for both genotyping and as a control for cDNA-based AEI measurements. To facilitate our long-term goal of screening several-hundred neuropsychiatric disorder candidate genes, we carried out genomewide genotyping for each of the 52 brains in our collection using Illumina HumanOmni1-Quad microarrays. For most genes, this information allowed us to infer the genotype of suitable mRNA marker SNPs (mSNPs) for most of our candidate genes. SNaPshot^® ^Multiplex kits (ABI) were used to genotype mSNPs not included or tagged by SNPs on the Illumina arrays.

**Table 1:** Numerical summary for Illumina Assay-2

Stage	gDNA/RNA	Number
0	Candidate genes	74
0	Independent brain samples	52
0	Genotypes	52 × ~1.14 × 10^6^
1	Isolated gDNA/cDNA	2 × 52 tubes
2	Individual PCR products	2762 tubes
2'	Supplemental PCR products	~200 tubes
3	Pooled, sample-specific PCR products	2 × 52 tubes
4	Sample-specific PCR products ligated to indexed Illumina adapters	2 × 52 tubes
5	Complete set of PCR products ligated to indexed Illumina adapters	2 × 1 tube
6	AEI Ratios	1,371 gDNA 1,313 cDNA

For each candidate gene, gene-specific PCR primers were used to amplify segments of gDNA or cDNA that contain the corresponding mSNP. To reduce the total number of PCR reactions, only gDNA and cDNA from brain samples heterozygous for the mSNPs were used as templates. To simplify the generation and analysis of the PCR reaction products, PCR amplifications of individual genes were carried out in sets, with each sample-primer combination in an individual tube. In all, 74 sets (one for each of the 74 candidate genes) comprising 3 to 36 gDNA template-based and 3 to 36 cDNA template-based reaction mixes were independently amplified (total: 2,762 reactions).

To ascertain the efficiency of the PCR amplifications, aliquots from each reaction mix were resolved by electrophoresis in 15% polyacrylamide gels, and the intensity and purity of the PCR products examined by ethidium bromide staining. Approximately 93% of the samples yielded robustly stained, single PCR products. In cases where staining of PCR products with the predicted molecular weight was weak, additional PCR reactions (~200) were carried out to increase the amount of PCR products carried to the next step and to ensure that a sufficient number of mRNA molecules were sampled to obtain meaningful AEI ratios (see Methods for details).

Following confirmation of amplification, approximately equal amounts of PCR products (one PCR product for each gene with a heterozygous mSNP) derived from a single brain sample were pooled, purified using QIAEXII beads and ligated to an indexed Illumina adapter coding for that sample (total: 52 gDNA template-derived samples + 52 cDNA template-derived samples). The ligated PCR products from all of the brain samples were pooled (total: one gDNA template-derived sample + one cDNA template-derived sample) and resolved by electrophoresis in 3% agarose gels. Ligation products in the appropriate size range (150 - 220 bp) were excised from the gels and used as templates for PCR amplification with Illumina Primers 1.1 and 1.2 (see Additional file 1, Figure S1). The resulting PCR products were sequenced using an Illumina Genome Analyzer 2.0. The above procedure (with minor modifications) was independently carried out twice (Illumina Assay-1 and Illumina Assay-2) for a subset of genes and brain samples, beginning from the isolation of gDNA and total RNA.

Following sequencing, reads were sorted, collated and tabulated for calculation of AEI ratios using a custom computer program, as described in Methods and Additional file 1, Figure S2. As shown in Additional file 1, Figure S3, approximately 85% of the sequencing reads were full-length (76 bp). Overall, approximately 60% of the reads survived quality-control triage and were used for the calculation of AEI ratios (Illumina Assay-2). In total, we obtained approximately 6 × 10^6 ^useable, independent reads for gDNA-derived sequences (1 flow cell lane) and 7 × 10^6 ^reads for cDNA-derived sequences (2 lanes) in Assay-1 and 13.2 × 10^6 ^reads for gDNA-derived sequences (2 lanes) and 18.5 × 10^6 ^reads for cDNA-derived sequences (5 lanes) in Illumina Assay-2. For the second assay, the number of reads/sample for individual genes (i.e., the total reads for M + m alleles) ranged from 1,079 to 33,250 for gDNA-derived sequences (average = 9,911) and from 1,277 to 75,440 for cDNA-derived sequences (average = 14,919) (data from Additional file 2, Table S5).

An important application of gDNA-based AEI measurements is the quantification of allele-specific bias that occurs during the PCR-amplification of gDNA and cDNA segments containing the marker SNP. As shown in Additional file 1, Figure S5, significant allele-specific amplification bias for gDNA templates was observed for most genes, with occasional genes showing greater than 1.5-fold differences in amplification between alleles. This type of bias has been previously observed in PCR-based AEI assays [21,30,31].

One documented source of allele-specific PCR amplification bias is the use of PCR primers that bind to sites containing a SNP [35]. As listed in Additional file 2, Table S6, nine of our gene-specific PCR primers bind DNA sequences containing nominal SNPs listed in the NCBI SNP database. Three of these SNPs, however, have 0 or very low heterozygosity in the Han Chinese population and therefore should not present a problem. One SNP has a heterozygosity of 0.162, but did not produce a significantly distorted log_2_AEI ratio. Among the remaining five genes, all with SNPs of unknown heterozygosity in the Chinese population, two showed significant deviation of log_2_AEI values from 0.

In addition to PCR amplification bias, the presence of pseudogenes, highly homologous genes or chromosomal duplications (e.g. copy number variants) can also distort genomic DNA AEI ratios. As described in Methods and Additional file 3, we belatedly found evidence for off-target sites in the human genome for the *CYP2D6 *and *NTAN1 *PCR primer sets. We found no evidence, however, for off-target sites for any of the other PCR primer set used in our study. Following previous examples [21,30,31], we therefore corrected both gDNA and cDNA ratios by multiplying the AEI ratios by the inverse of the average gDNA-based AEI for each gene. A list of the correction factors used to normalize AEI ratios for each gene is provided in Additional file 2, Table S5.

In addition to providing a method for correcting AEI measurements for PCR amplification bias, gDNA-based AEI measurements also yield important information concerning experimental error. Because only heterozygous individuals were included in our final data sets, deviations of normalized gDNA AEI ratios from 1 (or log_2 _normalized ratios from 0) can be used to estimate this error. Two different approaches were used to estimate experimental error. In the first, we determined the distribution of normalized gDNA AEI ratios for the entire set of measurements and calculated the mean, standard deviation (SD) and standard error of the mean (SEM) [details provided in Additional file 3]. This analysis showed that 95% of the normalized gDNA AEI ratios lay within the interval 0.82 to 1.22 (or ± 0.29 for log_2 _normalized gDNA AEI ratios). As described in Additional file 3, this estimate of experimental error was used to evaluate the presence or absence of AEI for each gene in each sample.

In the second method, also described in Additional file 3, experimental error was estimated based upon a comparison of deviations of log_2_AEI values from zero for normalized gDNA AEI ratios as a function of the number of sequencing reads used to calculate each ratio. As shown in Additional file 1, Figure S6(a), deviations from log_2_AEI = 0, are greater than those predicted from a theoretical binomial sampling error curve, but still relatively small and decrease with increasing numbers of sequencing reads. The legend of Additional file 1, Figure S6(b) describes an empirical method for estimating the correlation between the number of sampling reads and experimental error that does not require assumptions about the statistical distribution of the error. As listed in Additional file 2, Table S4, we estimate the log_2 _experimental error to be about ± 0.16 (i.e., linear AEI ratios between 0.895 and 1.117) for AEI measurements generated by 1,000 reads, ± 0.10 (linear ratios between 0.93 and 1.07) for AEI measurements generated by 13,500 reads and ± 0.09 (linear AEI ratios between 0.94 and 1.064) for AEI measurements generated by > 24,300 reads. As described below, this method for evaluating experimental error was used in the mathematical modeling of log_2_AEI population distributions.

### Log_2_AEI ratio distributions for 70 candidate neuropsychiatric disorder genes

**Figure 2:**
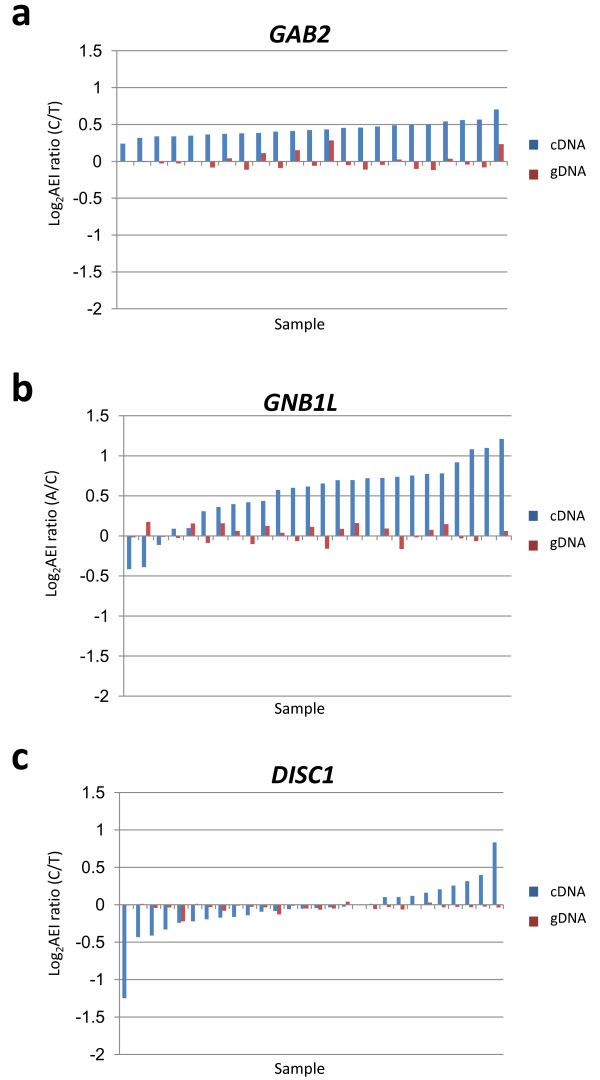
**Representative log**_**2**_**AEI distributions: *GAB2, GNB1L *and *DISC1***. Each pair of bars represents the gDNA-based (red) and cDNA-based (blue) log_2_AEI ratios from a single sample. cDNA-based log_2 _AEI ratios are arranged in order from most- negative on the left to most-positive on the right, forming a distinct distribution of log_2_AEI ratios for each gene. The selected genes illustrate three common patterns of log_2_AEI population distributions: uniphasic, skewed and biphasic. As described in the text and in Additional file 4, log_2_AEI distributions often contain useful information concerning the number, location and linkage of the regulatory genetic variants that produce AEI in each gene.

Figure 2 depicts representative results from our AEI assays for the three candidate neuropsychiatric disorder genes: (A) *GAB2*, (B) *GNB1L *and (C) *DISC1*. As shown in the figure, each of these genes produces robust cDNA-based log_2_AEI ratios (blue), that vary considerably in extent and direction compared to gDNA-based controls (red). To facilitate analysis, the samples are ordered from low-log_2_AEI to high-log_2_AEI (left-to-right). When the data is arranged in this fashion, it is apparent that each gene displays a characteristic distribution of log_2_AEI ratios. As explained below, these distributions often reflect differences among the regulatory variants that produce AEI with respect to: i) allele-frequencies, ii) relative contributions to log_2_AEI, and iii) degree of linkage to the mSNP.

**Figure 3:**
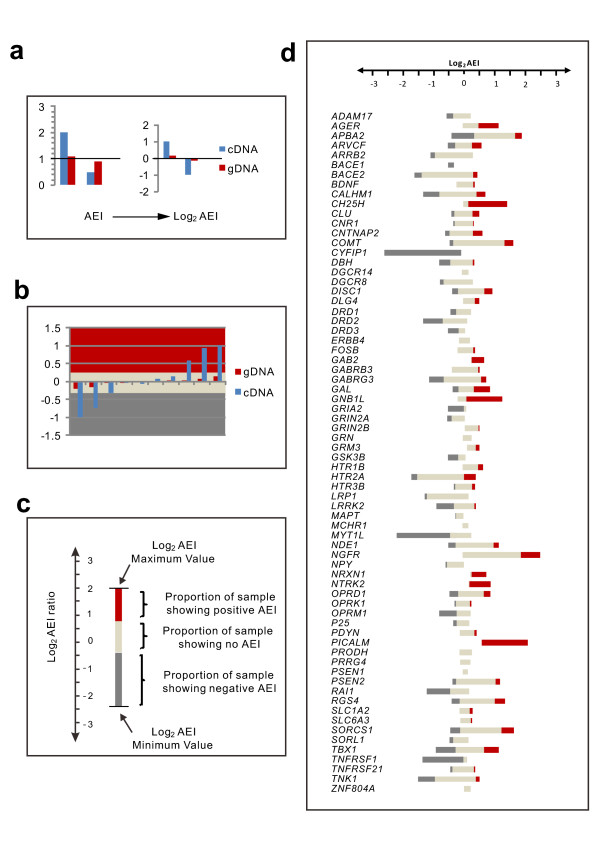
**Summary of AEI results**. Sections (a)-(c) provide keys for interpreting the diagram in (d), which summarizes the results of AEI measurements for 70 neuropsychiatric disorder candidate genes. The lengths of the grey, cream and red bars in (d) represent the percentage of samples with negative log_2_AEI ratios, no significant AEI and positive log_2_AEI ratios, respectively, for each candidate gene. The left-hand edge of each bar marks the smallest negative-AEI ratio and the right-hand edge the largest positive-log2 AEI ratio for the corresponding gene. Data used to produce this summary are provided in Additional file 2, Table S5.

Figure 3 provides a graphical overview of our AEI measurements for 70 genes. (Insufficient sequencing reads prevented calculation of AEI ratios for 3 of the original 74 candidate genes, and, as explained above, *CYP2D6 *was dropped due to co-amplification of *CYP2D7a *sequences.) Parts (a), (b) and (c) of the figure provide a key for interpreting the diagram in part (d). Figure 3(a) shows the relationship between linear AEI ratios and log_2_AEI ratios. In general, expressing AEI ratios in the log_2 _form has the advantage that displacements of positive and negative log_2_AEI ratios appear equal in magnitude, compared to graphs of linear ratios, which compress negative displacements. Figure 3(b and c) shows the color scheme for classifying log_2_AEI ratios as negative, non-significant or positive. (The mSNP alleles in the numerator and denominator of each AEI ratio are listed in Additional file 2, Table S1.) In this Figure, cDNA log_2_AEI ratios less than -0.29 or greater than +0.29 (corresponding to linear ratios less than 0.82 or greater than 1.22) were considered significant.

**Figure 4:**
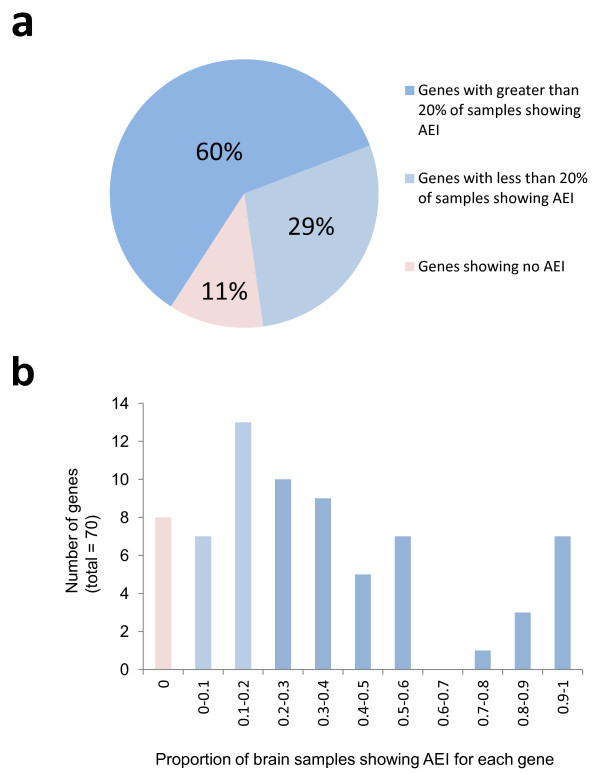
**AEI assay statistics**. (a) Approximately 60% (42/70) of our candidate genes produced significant AEI in at least 20% of the 52 brain samples, 29% (20/70) produced significant AEI in fewer than 20% of the samples and 11% (8/70) showed no AEI in any of the samples. (b) Histogram of the distribution of candidate gene with the indicated proportions of samples showing significant AEI.

As seen in Figure 3(d) there is considerable variability in mRNA expression among the 70 candidate disorder genes, with measured cDNA log_2_AEI ratios ranging from -2.65 (*CYFIP1*) to +2.79 (*NGFR*), corresponding to -6.27 and +6.9-fold differences in expression between alleles, respectively. Figure 4 provides a summary of the results from Illumina Assay-2. Surprisingly, 89% of our candidate genes show significant log_2_AEI ratios for at least one brain sample in our collection. Among samples heterozygous for the mSNPs for a particular gene, the proportion of samples with significant log_2_AEI ratios ranged from 5% to 100%, with an average of 36% and median 29%.

**Figure 5:**
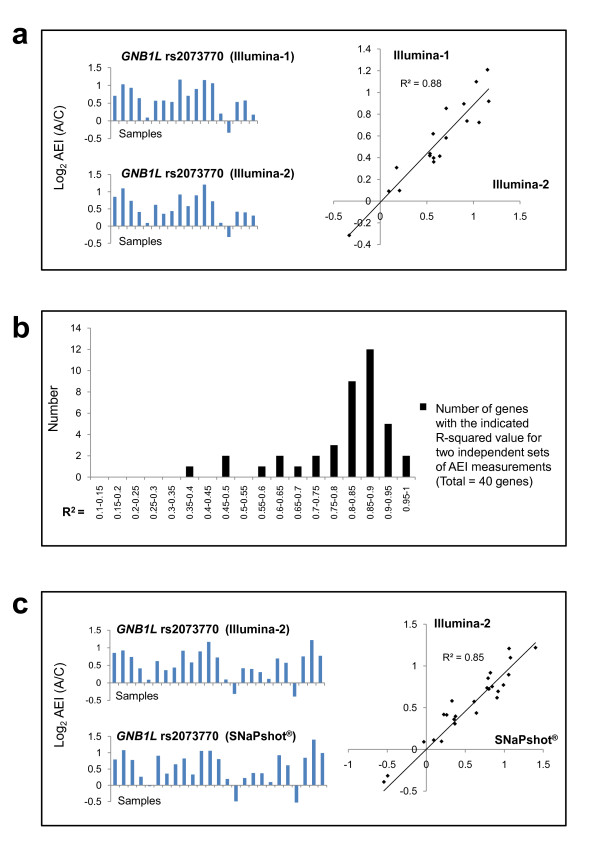
**Reproducibility of AEI assays (*GNB1L*)**. (a) [Left] Comparison of the results of independent measurements of *GNB1L *AEI. (Each assay was performed independently, from the isolation of brain RNA through DNA sequencing.); [Right] Regression analysis. (b) Histogram of the distribution of r^2 ^values obtained in regression analysis of independent AEI assays for 40 candidate genes for which replica assays were carried out. (All data are shown). (c) [Left] Comparison of the results of independent measurements of *GNB1L *AEI using PCR/DNA sequencing-based (top) and PCR/SNaPShot^®^-based (bottom) assays. [Right] Regression analysis. [Note: the Illumina-2 AEI ratios in (a) were matched with those obtained in the Illumina-1 AEI assay, which contained fewer samples. The full set of data from the Illumina-2 assay is shown in (c).]

Figure 5(a) shows that excellent agreement was obtained for independent measurements of log_2 _AEI ratios for *GNB1L*. Figure 5(b) summarizes the results of comparisons of log_2 _AEI ratio measurements using our PCR/DNA sequencing-based assay for 40 candidate genes. More than eighty-percent (33/40) of these genes yielded satisfactory-to-excellent coefficient of determination (r^2^) values (> 0.7) when comparisons were analyzed by linear regression analysis. For some genes, low r^2 ^values reflect the shape of their log_2_AEI distributions (e.g., uniphasic, with a small range of log_2_AEI values) or small sample number. Graphs showing the linear regression analysis of 12 representative genes are provided in Additional file 1, Figure S7. To further validate the results of our DNA sequencing-based assay, we independently measured AEI ratios for several genes using a PCR/primer-extension (SNaPshot^®^)-based assay, which has been extensively verified in previous studies [20,30,31]. Figure 5(c) shows excellent agreement between these two methods for *GNB1L*.

### Modeling log_2_AEI distributions

As described above, log_2_AEI distributions are characteristic for specific gene/mSNP pairs and highly reproducible. To better understand these patterns, we developed a simple mathematical model that can mimic distributions of log_2_AEI ratios with surprising accuracy. Modeling the three candidate neuropsychiatric disorder genes *GAB2, GNB1L *and *DISC1 *is briefly described in Additional file 4. A comprehensive description of this model will be presented in a separate paper (Sun Y, *et al*, manuscript in preparation).

According to our model, the shapes of log_2_AEI distributions are influenced by: i) the number of *cis- *and *trans*-acting regulatory variants (rVar) [rVarA, rVarB, rVarC, rVarT, etc.], ii) their major allele frequencies [P(A), P(B), etc.] iii) the contribution of each variant to log_2_AEI ratios [j, k, l, m, etc], iv) the degree of linkage disequilibrium between rVar's [D'(AB), etc.], v) the degree of linkage disequilibrium between each rVar and the mSNP [D'(AM), D'(BM), etc.] and vi) complex genetic, epigenetic or non-genetic factors.

**Figure 6:**
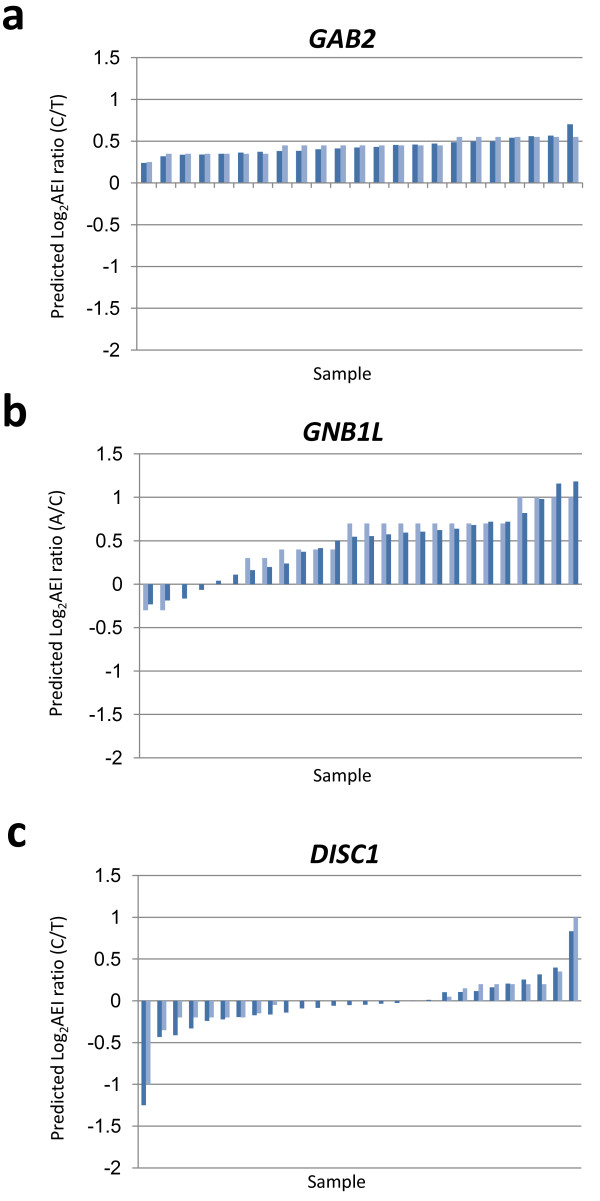
**Modeling log_2_AEI population distributions**. Predicted log_2_AEI distributions for (a) *GAB2*, (b) *GNBIL *and (c) *DISC1 *were calculated using the mathematical models described in Additional file 4. For each gene, the population frequency of the mSNP, P(M), was experimentally determined for our collection of samples. Dark blue: experimental data; light blue: predicted log_2_AEI ratios

A comparison of the predicted and experimentally determined log_2_AEI distributions for *GAB2, GNB1L *and *DISC1 *is shown in Figure 6. In the case of *GAB2*, the observation that the log_2_AEI ratios are all in the same (positive) direction suggests that mRNA expression is primarily regulated by single *cis*-acting regulatory variant that is in complete linkage disequilibrium with the mSNP. By contrast, the skewed log_2_AEI distribution obtained for *GNB1L*, in combination with a gradient in log_2_AEI values and the presence of several samples with negative or non-significant log_2_AEI ratios, suggests that mRNA expression is regulated by two genetic variants, a *cis*-acting variant partially linked to the mSNP and the other unlinked or weakly linked to the mSNP. Finally, for *DISC1*, the nearly equally balanced, biphasic AEI population distribution, suggests the influence of multiple regulatory variants, which are all unlinked (or very weakly linked) to the mSNP. Details of the modeling of these genes are presented in Additional file 4 and a discussion of the results with respect to previously published studies is presented in Additional file 5.

## Discussion

In this paper we describe a PCR/next-generation DNA sequencing-based method for quantifying allele-specific differences in mRNA expression and demonstrate that it produces detailed and highly reproducible information concerning the expression of candidate neuropsychiatric candidate genes in human brain. The two major strengths of our assay are the ability to: 1) reproducibly measure small differences in allelic expression for both common and rare mRNAs and 2) generate population distributions of log_2_AEI ratios that are useful for generating testable hypotheses concerning the number, location and relative contributions of genetic variants that regulate mRNA expression.

When analyzing large numbers (50 - 200) of genes and samples (50 - 100), the cost of our assay is approximately one-half and hands-on time approximately one-third that of low-throughput PCR/primer extension (SNaPshot^©^)-based assays. The use of DNA-sequencing instead of SNaPShot^©^-based primer-extension as the assay read-out obviates the gene-by-gene fine-tuning often required for SNaPshot assays, as well as the influences of differential efficiencies of incorporation of fluorescently labeled dideoxynuclotides into DNA and differences in the quantum efficiency of the fluorophores, which can distort AEI ratios. Experimental error in SNaPshot^©^-based assays varies among genes and generally only allows quantification of AEI ratios greater than 1.2. By contrast, given sufficient numbers of sequencing reads, AEI ratios as low as 1.1 can often be reliably measured using our method.

Our AEI assay also compares favorably to previously developed high-throughput methods. Several studies have described AEI assays that are based on differential hybridization in microarrays [36-39]. While each of these assays is capable of detecting many thousands of differentially expressed transcripts in a single experiment, two of the studies [36,38] reported that reliable AEI could only be detected for linear AEI ratios greater than 1.5. The other two studies [37,39] described an assay that lacked a PCR amplification step, potentially limiting the ability to use the assay to measure AEI ratios for rare mRNA transcripts in brain.

In addition to microarray-based assays, high-throughput AEI assays based on PCR and second-generation DNA sequencing have also recently been described [40-42]. Main BJ *et al*. [40] developed an AEI assay similar in design to ours, which they successfully used to analyze *cis*- and *trans*-regulation of five *Drosophila simulans *genes. Zhang K *et al*. [41] and Lee *et al *[42] established a "digital RNA allotyping" assay, based on deep sequencing of "padlock" oligonucleotide probes designed to distinguish alleles of 27,000 exonic SNPs (minor allele frequency > 0.07). In this assay, genotyping calls were based on a minimum of 20 sequencing reads and AEI ratios for mRNAs expressed in human cells were based on a minimum of 50 sequencing reads.

Several additional studies have used whole transcriptome sequencing (RNA-seq) [43,44] strategies for genomic-scale detection of AEI [45-49]. Although this approach provides valuable information about the spectrum of mRNA in specific tissues, including information concerning the expression of splicing variants, the depth of coverage is usually not sufficient to obtain accurate AEI ratios. Although several studies calculated AEI ratios based on as few as 50 sequencing reads, Fontanillas *et al*. [48], using 454 sequencing technology to quantify mRNA expression in *Drosophila*, established that 500 to 1,000 sequencing reads are required to accurately quantify AEI ratios. This range is consistent with the results of our analysis of experimental error as a function of sequencing reads (Additional file 1, Figure S6 and Additional file 2, Table S4).

To date, three major studies have used microarray-based mRNA expression assays combined with genome-wide genotyping to quantify mRNA expression and identify eQTL's for genes expressed in human brain [16-18]. Although each of these studies yielded important information concerning mRNAs that could be detected, each also lacked the sensitivity to detect mRNAs for many important candidate disease genes.

Myers *et al*. [16] used the Illumina HumanRefseq-8 Expression BeadChip system to quantify levels of 14,078 transcripts in RNA isolated from 193 independent samples of human cortex. Screening SNPs located within approximately 2.1 M bp chromosomal segments centered on each gene for correlations between genotype (additive model) with mRNA expression yielded significant associations for 433 SNP-transcript pairs (in 99 transcripts = 0.7% of detected mRNA transcripts), with the majority of the associated SNPs located within 70 kb of the transcript. Although each of these *cis*-associations was significant after correction for SNPs tested in the same region, only two genes, *KIF1B *and *IPP*, remained significant after corrections for multiple testing for all SNPs-transcript pairs that were studied. Furthermore, with the exception of *MAPT *[26], no genes previously demonstrated to exhibit AEI in human cerebral cortex in PCR-based AEI assays, including *DTNBP1 *[22], *COMT *[23], *GNB1L *[50], *TBX1 *[50], *OPRM1 *[28], *DRD2 *[29], and *CHRNA5 *[33] were included in the list of nominally significant *cis *SNP-transcript pairs.

In a follow-up study by the same group, Webster *et al *[17] used more stringent inclusion procedures for expressed transcripts, analyzing 8650 transcripts in 486 late-onset Alzheimer's disease (LOAD) cases and 279 control brain samples. Again, none of the AEI-positive genes listed above was correlated with a SNP located in the region of the gene, although a SNP located on chromosome 1 was weakly correlated with expression of *GNB1L *mRNA in the set of AD brain samples (r^2 ^= the contribution of SNP rs7527404 to the variance of transcript expression = 0.13).

Using expression data from 269 brain samples available on the Stanley Medical Research Institute Online Genomics database (https://www.stanleygenomics.org), in-house genotyping on Affymetrix Human Genome U133A microarrays, and careful pre-screening of the of the expression and genotyping data to eliminate the effects of confounding variables, Liu *et al*., [18] identified 903 SNP-expression probeset pairs (in 826 genes) that showed significant "region-wide" association (i.e., significant after correction for multiple testing of SNPs in the region of the gene for correlations between genotype and expression). Among these, 562 SNP-expression probeset pairs (in 106 genes) also showed significant "phenotype-wide" association (i.e., significant after correction for multiple testing for all genotype-expression correlations examined).

Although the study of Liu *et al*., identified more genes that are regulated by *cis*-acting genetic elements compared to the study of Myers *et al*., with the exceptions of *GSTM3 *[51] (significant at both the "region-wide" and "phenotype-wide" levels) and *COMT *[23] (significant at the "region-wide," but not the "phenotype-wide" level), genes previously shown in PCR-based assays to have significant AEI in human cortex were not detected. As noted by the authors, the microarray-based expression assays also did not detect mRNA levels sufficient for analysis for many additional neuropsychiatric disorder candidate genes.

Among the 70 genes examined in the present study, *MAPT *was reported by Myers *et al*. [16] to have significant P-values, but the remaining 69 were not detected. In addition to *GNB1L*, Webster *et al*. [17] reported correlations between transcript levels and *trans*-SNPs for an additional three of our candidate genes, *CLU, NPY *and *PICALM*, but no correlating *cis*-SNPs. In the study of Liu *et al *[18], four of our candidate genes, *HTR2A, LRP1, NTAN1 *and *GRIA2*, attained significant "region-wide" P-values, but not "phenotype-wide" P-values, 23 genes were not significant at either level, and 44 were not detected at levels sufficient for analysis.

Taken together, the results of these three studies strongly suggest that PCR-based mRNA expression assays will prove to be the method of choice for investigating genetic regulation of many important disease candidate genes in human brain. An important limitation of this method that should be mentioned, however, is that some candidate genes lack suitable mSNPs for AEI measurements or contain only mSNPs with very low heterozygosity. For such genes, the traditional approach of looking for correlations between relative mRNA expression (e.g., measured by real-time PRC) genotypes of candidate regulatory SNPs is the next-best option.

By contrast to previously published studies, our study suffers somewhat from an "embarrassment of riches" in that a very large percentage of our genes showed differential mRNA expression among the samples tested. For example, previous genome-wide studies of eQTL's and allele-specific expression (mostly in cell lines for transformed human lymphocytes) yielded estimates for differentially regulated genes in the range of 10 - 54% [52,53]. By contrast, studies focusing on sets of candidate disease genes, have consistently detected higher percentages of genes showing AEI: greater than 50% [20,22,54]. The observation that candidate genes are enriched for genes that show allele-specific differences in mRNA expression is consistent with the hypothesis that variation in gene expression contributes to susceptibility to complex diseases [10]. Consistent with this idea, a recent study showed that putative schizophrenia susceptibility alleles are enriched for alleles that influence mRNA expression in human brain [55].

In addition to focusing on leading candidate genes for schizophrenia, Alzheimer's disease and drug addition, another reason for the high percentage of genes showing AEI in our study is the accuracy of our assay, which allows small deviations from 1.0 to be detected with confidence. If experimental error based on sequencing read number is taken as the sole standard, our assay reliably detects AEI ratios was small as 1 ± 0.1. Taking ± 0.1 as the cut-off, almost all of our 70 candidate genes show AEI in at least one sample. These observations suggest that small allele-specific deviations from 1.0 are nearly ubiquitous and raise the question concerning when allele-specific differences in mRNA expression become biologically meaningful.

Certainly the existence of AEI is not equally important for all genes. Rather, biologically important AEI is likely to be restricted to genes that encode proteins that are limiting for important biological processes. An example of such a gene is *TPH2*, which encodes tryptophan hydroxylase-2, the rate-limiting enzyme in the synthesis of serotonin in the brain [56]. For such "dose-sensitive" genes, even small changes in mRNA expression may have a significant biological effect, if the changes in mRNA are reflected in changes in protein level and/or enzymatic activity.

A good example of a dose-sensitive gene that does not encode an enzyme is *APP*, encoding amyloid precursor protein, the precursor of the toxic peptide Aβ, which accumulates in the brains of patients with Alzheimer's disease (AD) [57]. Duplication of the *APP *gene, as the result of rare chromosomal duplications or, as in the case of Down's syndrome, duplication of the entire chromosome 21, is strongly associated with early-onset AD [58]. Rare promoter mutations that modestly increase *APP *expression are also linked to early-onset AD [59]. If duplication of *APP*, which may increase mRNA expression by 50%, can have a dramatic effect on the risk and age of onset of AD, it is not unlikely that smaller increases in mRNA expression, e.g., 10% - 40%, contribute to the more common late-onset AD (LOAD). The same argument holds for genes encoding enzymes and other proteins that function in the production, degradation, clearance and deposition of Aβ. Clearly analysis of as many genes as possible in these pathways and estimating their combined effects will be important to assessing the relative susceptibility to AD. We believe that our assay provides the optimal balance between throughput and sensitivity that will be useful for investigating sets of genes that function within specific biological pathways implicated in AD and other neuropsychiatric disorders.

One of the major implications our AEI measurements and mathematical modeling is that, for many genes, mRNA expression is a "complex phenotype," involving 2, 3 or more *cis-*acting regulatory variants (or, in some cases, at least one *cis*-acting variant plus additional *trans*-acting variants). These results are consistent with detailed studies showing that specific genes are regulated by multiple *cis*- and *trans*-acting variants [60-62]. Based upon our modeling, at least two *cis*-acting regulatory variants are required to account for the observed spectrum of *GNB1L *log_2_AEI ratios in our brain samples. This implies that predicting high- and low-expression mRNA will require the identification of two variants, or, alternatively, two "indicator" (i) SNPs that are tightly linked to these variants and accurately predict high- and low-mRNA expression. Haplotypes comprised of high- or low-expression alleles of regulatory variants or iSNPs would provide the best genetic markers to test the hypothesis that differential expression of *GNB1L *contributes to risk of developing schizophrenia. One of the major goals of our AEI studies is to identify the best possible genetic markers for association studies for this and other candidate neuropsychiatric disorder genes.

In summary, we believe that the AEI assay and molecular modeling described in this study will provide useful tools for investigating the genetic basis of complex diseases, including major neuropsychiatric disorders. These methods should be immediately useful for investigating, in a comprehensive manner, the possible contributions of genes that function within specific biological pathways and systems that contribute to disease. They should also be useful for comparing the regulation of specific genes in developing and mature brain [55], investigating region- and cell-type specific mRNA expression [63], integrating information about expression and splice variants [64,65] and investigating epigenetic mechanisms of gene regulation [66,67].

## Conclusions

In this study we describe a novel, PCR and next-generation DNA sequencing-based method for quantifying allelic expression imbalance (AEI) of mRNA expression in human brain. We show that this assay produced detailed and highly reproducible measurements of AEI ratios for 70 neuropsychiatric disease candidate genes. We also demonstrate that population distributions of log_2_-transformed AEI ratios for individual gene/marker SNP pairs can provide important information concerning the number, location and effect size of regulatory variants that influence mRNA expression. Taken together, our assay and mathematical modeling provide powerful tools for analyzing the genetic regulation of candidate disease genes and should be useful for investigating how regulatory genetic variants contribute to complex human disorders.

## Methods

### Selection of Candidate genes and marker (m)SNPs

Candidate genes for this study were selected based on their relevance to neuropsychiatric disorders currently under investigation in the Saffen laboratory: i) schizophrenia/autism, ii) Alzheimer's disease and/or iii) heroin addiction. Particular attention was given to genes that had been previously linked to one or more of these disorders in genetic association studies or are located within copy number variations (CNVs) that associate with schizophrenia and/or autism. To allow allele-specific quantification of mRNA expression, candidate genes mRNAs were required to contain at least one single nucleotide polymorphism (SNP) with high heterozygosity in the Han Chinese population for use as a marker SNP. Most of the selected candidate genes had not previously been tested for allelic expression imbalance (AEI) in human brain, although several previously studied genes were included for the purpose of comparison and verification. A list of the 74 genes included in this study can be found in Additional file 2, Table S1.

### Human Brain tissue

Frozen sections of human brain from: i) prefrontal cortex (Brodmann Area 46), ii) hippocampus, iii) amygdala, iv) ventral striatum/nucleus accumbens, v) substantia nigra, and vi) rostral pons from 52 Han Chinese individuals were obtained from the Chinese Brain Bank Center (South-Central University for Nationalities, Wuhan, China). This sample population comprised 25 males and 27 females (ages: 1 to 70; average 41) and cause of death, included illness, traffic accidents, electronic shock and heart-attacks. Postmortem intervals were less than 36 hours (most less than 24 hours). In all cases, written consent for tissue donation was obtained from relatives (on file at CBBC). Use of human autopsy tissue is considered non human-subject research and is IRB exempt under NIH guidelines.

### Isolation of genomic DNA and total RNA

Frozen brain tissue (~ 30 mg) was homogenized in 180 μl of DNA lysis buffer + 20 μl proteinase K (QIAamp^®^DNA Mini kit, Qiagen) and genomic DNA (gDNA) isolated, according to the instructions of the manufacturer (Qiagen, Valencia, CA, USA). For total RNA, frozen brain tissue (~ 100 mg) was homogenized in (1 ml) Trizol reagent (Invitrogen, Carlsbad, CA, USA), and total RNA isolated as recommended by the manufacturer. Following resupension in 30 μl RNAase-free water containing 2 μl RNaseOUT™ (Invitrogen), contaminating genomic DNA was eliminated by incubating at 37°C in the presence of 30 U RNase-free DNase I (New England Biolabs) for 30 min. The RNA was then purified using Qiagen RNeasy Mini kits, as recommended by the manufacturer, resuspended in 30 μl RNAase-free water and stored at -80°C. The quantity of gDNA and total RNA was determined using a Nanodrop spectrophotometer (Thermo Inc). [Note: incubation of total RNA with large quantities of DNase I was required to prevent amplification of small genomic DNA sequences during PCR-amplifications using cDNA templates.]

### Genotyping

Genomewide genotyping was carried out using HumanOmni1-Quad genotype arrays (Illumina) for each of the 52 independent samples in our study (~ 1.14 × 10^6 ^genotypes/sample). For SNPs not included in these arrays, additional genotyping was carried out using SNaPshot^® ^Multiplex kits (Applied Biosystems), as previously described [30,31]. Gene-specific PCR primers were designed using Oligo 6.0 (National Biosciences Inc., Plymouth, MN, USA) and synthesized by Sangon Biotech (Shanghai, China). For most genes, the same pairs of PCR primers (forward and reverse) were used for genotyping and amplification of cDNA sequences. Sequences of these primers are listed in Additional file 2, Table S2. In cases where forward and reverse PCR primers designed for amplifying cDNA sequences are located on different exons, alternative primer sets were used for genotyping and for measuring gDNA AEI ratios. These primers are labeled "G" in Table S2.

After completion of our experiments, we discovered that primer sets for two candidate genes, *CYP2D6 *and *NTAN1*, amplified off-target segments of DNA (see notes at the end of Additional file 2, Table S2). Because of possible conflation of genotyping and mRNA expression data with a highly homologous pseudogene, *CYP2D6 *was dropped from the list of "successfully analyzed" genes. By contrast, the *NTAN1 *primers failed to produce sufficient PCR products for AEI analysis. Analysis of primer sets for all the other candidate genes using NCBI's PRIMER-BLAST (http://www.ncbi.nlm.nih.gov/tools/primer-blast/) and/or UCSCs *In-silico *PCR (http://genome.ucsc.edu/cgi-bin/hgPcr?command=start) programs predicted amplification of only the intended target sequences.

### cDNA synthesis

Complementary DNA was generated from 5 μg total RNA in 20 μl reaction mixes using SuperScript^® ^III First Strand kits (Invitrogen, Carlsbad, CA, USA) and 1 μl of 50 μM oligo(dT)_20 _primers, according to the directions of the manufacturer. cDNA reaction mixes were diluted 2x with DNAase- and RNAase-free water (Sigma; final volume 40 μl) and stored at -20 °C until use.

### Screening brain samples for expression of target mRNAs

To obtain meaningful and reproducible AEI ratios, it is essential that the RNA samples contain a sufficient number of candidate gene mRNA molecules. Our previous experience suggests that a minimum of about 1,000 mRNA molecules is required to obtain stable AEI ratios for many genes, an estimate consistent with a recently published study [48]. To determine the appropriate brain section for assessing AEI of candidate genes, PCR amplification was first carried out using cDNA derived from purified prefrontal cortex RNA. Reaction mixes (20 μl total volume) included: 1 μl cDNA (prepared as described above), 0.5 μΜ (total) forward and reverse PCR primers, 0.8 mM dNTP mix, 0.5 Units rTaq (Takara), and 1x PCR buffer (Takara). The amplification conditions comprised: 1x [94°C for 5 min], 28-30 cycles of [i) denaturation: 94°C for 30 sec; ii) annealing: 50-65 °C for 45 sec and iii) elongation: 72°C for 30 sec], followed by a final elongation [72°C for 5 min] and short-term storage at 4°C or long-term storage at -20°C.

For genes that generated sufficient PCR products to produce strong, single bands on polyacrylamide gels (minimum 50 ng/band after 30 cycles from 5 μl aliquot of PCR reaction mix), prefrontal cortical RNA was used for AEI analyses. For genes that did not yield sufficient PCR products (less than 50 ng/band after 30 cycles from 5 μl aliquot of PCR reaction mix), PCR products from 2 to 3 independent cDNA synthesis reactions were pooled or RNA isolated from other brain regions was used as starting material. A list of the brain regions used for AEI analysis for each gene can be found in Additional file, Table S5. [Note: Assuming a PCR amplification efficiency of 2 and calculating backwards, 50 ng/band = 200 ng total PCR product obtained after 30 cycles can be estimated to derive from approximately 1,800 molecules of mRNA.]

### PCR amplification of genomic and cDNA segments containing mSNPs

Segments of gDNA or cDNA containing mSNPs were PCR-amplified using 5'-phosphorylated oligonucleotide primers flanking the marker SNP. Gene-specific primer pairs were designed to generate PCR products 66 - 100 bp in length, with mSNPs located approximately in the center. An upper limit of 100 bp was chosen to ensure that mSNPs would be contained within 76 bp sequencing reads beginning from either end of the PCR product. The sequences of the gene-specific PCR primers are listed in Additional file 2, Table S2. PCR reactions mixes and amplification conditions are the same as those listed above. To minimize the total number of PCR amplifications, we used only genomic and cDNA templates from brain samples heterozygous for the mSNP for any particular candidate gene. A list of the number of brain samples heterozygous for the mSNP for each candidate gene is provided in Additional file 2, Table S5.

Following amplification, 5 μl aliquots of the 20 μl reaction mixes were resolved by electrophoresis on 15% polyacrylamide gels and amounts of PCR products of the predicted size quantified using the Molecular Imager^®^XGel Doc XR+ System X with Quantity One^® ^1-D Analysis Software (Bio-Rad Laboratories). Approximately equal amounts of PCR products derived from each candidate gene from a single brain sample were combined and purified using QIAEXII, according to the directions of the manufacturer (Qiagen, Valencia, CA, USA). Following resuspension in 40 μl distilled water, the amounts of the pooled PCR products were quantified using a Nanodrop Spectrophotometer (Thermo, Inc). In all, 2 × 52 sets of pooled PCR products were prepared, two sets (i.e., one gDNA-derived and one cDNA-derived) for each of the brains in our collection.

### Design and ligation of index-adapters

Fifty-two double-stranded index-adapters were synthesized by Sangon Biotech (Shanghai). Each adapter contained a five-base pair index linked to Illumina adapter sequences. The five-base pair index sequences were designed following Craig DW *et al*. [68], with identical nucleotides at the 1^st ^and 5^th ^positions to provide redundancy. A list of the index sequences and corresponding sample numbers can be found in Additional file 2, Table S3. As shown in Additional file 1, Figure S1, one strand of each index-adapter contained an additional unpaired T residue at the 3'-end, while the second strand was phosphorylated at the 5'-end to facilitate ligation to the gene-specific PCR products.

The 2 × 52 sets of pooled PCR products (1.5 μg each) were independently ligated to 52 sets of indexed Illumina adaptors (3 μg each) using T4 DNA ligase (New England Biolabs) at 16 °C overnight and then heated at 65 °C to inactivate the ligase. All of the ligation products were then combined into 2x one tube (i.e., one for gDNA-derived PCR products and one for cDNA-derived PCR products) and purified and concentrated using QIAEXII beads. The pooled ligation products were resolved by electrophoresis on 3% agarose gels. DNA fragments in the 150 to 220 bp range were extracted from the gels using MiniElute Gel Extraction Kits (Qiagen).

### Final steps in DNA sample preparation

To prepare the DNA fragments for sequencing, the ligated gDNA- and cDNA-derived DNAs were independently PCR-amplified using Illumina Primers 1.1 and 1.2 and Phusion DNA polymerase (Finnzymes Oy). The PCR products were resolved by electrophoresis on 15% polyacrylamide gels and DNAs of the appropriate size extracted using QIAquick Gel Extraction Kits (Qiagen). The concentration and purity of the DNAs were accessed spectrophotometrially (First run: gDNA sample = 55.8 ng/μl; A_260_/A_280 _ratio = 1.85; cDNA sample: 52.3 ng/μl; A_260_/A_280 _ratio = 1.89; second run: gDNA sample = 38.2 ng/μl; A_260_/A_280 _ratio = 1.91; cDNA sample = 51.4 ng/μl; A_260_/A_280 _ratio = 1.91). A summary of the number of independent samples (tubes) required for each step in the preparation of DNA samples for sequencing in provided in Table 1.

### Illumina sequencing

DNAs were prepared for sequencing on an Illumina Genome Analyzer 2.0 as described in the Single Read Cluster Generation Kit (v3) and SBS Sequencing Kit (v3) User Guides. Sequencing was carried out in the Laboratory of Epigenetics, Institutes of Biomedical Sciences, Fudan University. For Illumina assay-2, two flowcell lanes were used for sequencing gDNA-derived PCR products and five flowcell lanes for cDNA-derived PCR products. The average yield was 7.54 × 10^6 ^independent sequencing reads/lane, for a total of 52.8 × 10^6 ^independent reads. The distribution of read lengths is shown in Additional file 1, Figure S3(a).

### Data analysis

As outlined in Additional file 1, Figure S2, sequencing data from the Illumina Genome Analyzer 2.0 was matched to a custom sequence library using BLAST 2.2.25 (NCBI). The library comprised all possible combinations of indices and candidate gene sequences (52 indices × 74 genes × 2 insert directions = 7,696 reference sequences). Each sequencing read was transferred to a folder corresponding to its top match and sequences within each folder were aligned using MUSCLE 3.86 [69]. The SNP loci was then identified and the accuracy of the read classifications and alignments confirmed by comparing 20 bp sequences upstream and downstream from the mSNP. The number of each mSNP allele was tabulated and AEI ratios calculated using the mSNP allele assignments (numerator and denominator) listed in Additional file 2, Table S1.

### Verification

Reproducibility of AEI measurements were evaluated by linear regression analysis of log_2_AEI ratios obtained in independent DNA-sequencing-based assays. For selected genes, AEI ratios were also determined using SNaPshot^®^-based AEI assays, and the closes results evaluated by regression analysis. Representative examples of regression analysis are shown in Additional file 1, Figure S7. [Note: using regression analysis to compare replicate samples works well for log_2_AEI distributions that span a large range of values (negative to positive), but is not very informative for log_2_AEI distributions that span a small range of values. In addition, AEI ratios produced by low-frequency variants replicate poorly, when small numbers of samples are compared.]

## Authors' contributions

XX, HW, MZ: developed the DNA sequencing-based AEI assay, selected candidate genes, prepared DNA samples for sequencing and analyzed data for genes related to schizophrenia/autism, opioid addiction, and Alzheimer's disease, respectively; XX developed the methods for analyzing experimental error and defining the absence or presence of AEI. He also contributed to discussions concerning modeling log_2_AEI population distributions. YS, YT, QH & JW: prepared DNA samples for sequencing, analyzed AEI data and tested mathematical models for log_2_AEI distributions. YS extensively assisted in modeling log_2_AEI population distributions. LC helped design the DNA sequencing-based AEI assay and set up the SNPaShot^®^-based AEI assays; DS: proposed and supervised the development of the DNA sequencing-based AEI assay, selected candidate genes, developed the mathematical model for log_2_AEI distributions, analyzed data and wrote the paper. All authors read and approved the final manuscript.

## Supplementary Material

Additional file 1 **Supplementary figures S1 - S7**. Supplemental figures related to our next-generation DNA sequencing-based AEI assay **Figure S1 - Preparation of samples for DNA sequencing**. This diagram shows molecular details involved in preparing DNA samples for sequencing using the Illumina Genome Analyzer 2.0. **Figure S2 - Flow diagram for sorting DNA sequence reads**. This figure outlines the steps carried out by a computer program developed in- house for the calculation of AEI ratios using data produced by Illumina sequencing. **Figure S3 - Percentage usable sequences**. (a) Histogram of sequencing read lengths. Approximately 85% of the sequences were 76 bp in length following 76 sequencing cycles. (b) Among approximately 52.8 × 10^6 ^sequencing reads, 30% failed to meet match criteria in the BLAST step and were discarded. Another 3% were discarded due to missing sequence data for the marker SNP. Finally, 7% of the reads yielded sequences containing only one of the two mSNP alleles (reflecting genotyping errors or mistaken genotype imputation for the mSNP in some samples), yielding approximately 31.7 × 10^6 ^reads suitable for calculating AEI ratios. **Figure S4 - Distribution of sequencing read numbers used for the calculation of gDNA- and cDNA-based AEI ratios**. Histograms showing the distribution of (a) 13.2 × 10^6 ^gDNA reads and (b) 18.5 × 10^6 ^cDNA reads (mSNP M-allele + m-allele) among the 70 candidate genes in this study. (Data from Additional file 2, Table S5) **Figure S5 - Distribution of non-corrected gDNA-based AEI ratios **(a) Distribution of experimentally determined gDNA AEI ratios. The ideal AEI ratio for heterozygous samples = 1. (b) Regression analysis show that there is no correlation between sequencing read number and calculated gDNA ratios. **Figure S6 - Error analysis **(a) Plot of gDNA log_2_AEI ratios *vs *number of sequencing reads with super-imposed plot of the theoretical binominal sampling distribution (red trace), which was calculated based upon the assumption that the *M*- and *m*-alleles of the mSNP occur at equal frequency (0.5) in gDNA isolated from individuals who are heterozygous for the mSNP. (b) Definition of experimental error (E) based upon the distribution of [gDNA log_2_AEI ratio, sequence read number] data points. The two horizontal lines are drawn at the same distance above and below the X-axis, passing through the Y-axis at E and -E, respectively. The vertical line denoted "X (reads)" forms the left side of a rectangle that contains the data point with the highest sequencing read in the experiment. When the horizontal lines are adjusted so that the rectangle contains 95% of the data points with sequencing reads greater than X, the values log_2_E and - log_2_E represent the maximal ±log_2 _experimental errors of the measurement with 95% confidence. When the horizontal lines are adjusted so that the rectangle contains 99% of the data points with sequencing reads greater than X, the new values of + log_2_E and - log_2_E represent the maximum ± log_2 _experimental errors at 99% with confidence. (c) Empirically determined correlations between experimental error (E) and sequencing reads. A custom computer program was used to calculate correlations between sequencing read number (X) and ± log_2_E at 95% and 99% confidence levels. **Figure S7 - Examples of correlations between independent AEI assays**. Representative linear regression analyses for 15 candidate genes are grouped by level of statistical significance (P) for the correlation. Click here for file

Additional file 2 **Supplementary tables S1 - S6**. Detailed information concerning our candidate neuropsychiatric disorder genes, PCR and sequencing primers, experimental error and measured AEI ratios. **Table S1 - Neuropsychiatric disorder candidate genes. Table S2 - PCR primer sequences. Table S3 - Index sequences. Table S4 - Estimation of Experimental Error. Table S5 - Data for AEI ratio measurements (Illumina Assay-2) Table S6 - SNPs within PCR primer binding sites.** Click here for file

Additional file 3 **Correction factors for AEI ratios and criteria for the presence or absence of AEI in individual samples**. A discussion of factors that influence the measurement of genomic DNA AEI ratios and criteria for assessing whether individual samples show allele-specific differences in mRNA expression. Click here for file

Additional file 4 **Modeling population distributions of log**_**2**_**AEI ratios**. A brief outline of our method for modeling AEI ratios, including a description of the modeling of log_2_AEI population distributions for *GAB2, GNB1L *and *DISC1*. Click here for file

Additional file 5 ***GAB2, GNB1L *and *DISC1*: AEI measurements and modeling**. A discussion of inferences drawn from the modeling of *GAB2, GNB1L *and *DISC1 *in the context of previously published studies on the regulation of these genes. Click here for file

## References

[B1] Burmeister M McInnis MG Zollner S Psychiatric genetics: progress amid controversy Nature reviews 2008 9 7 527 540 10.1038/nrg2381 18560438

[B2] Wray NR Visscher PM Narrowing the boundaries of the genetic architecture of schizophrenia Schizophrenia bulletin 2010 36 1 14 23 10.1093/schbul/sbp137 19996148 PMC2800151

[B3] Kim Y Zerwas S Trace SE Sullivan PF Schizophrenia genetics: where next? Schizophrenia bulletin 2011 37 3 456 463 10.1093/schbul/sbr031 21505112 PMC3080692

[B4] Bertram L Lill CM Tanzi RE The genetics of Alzheimer disease: back to the future Neuron 2010 68 2 270 281 10.1016/j.neuron.2010.10.013 20955934

[B5] Ballard C Gauthier S Corbett A Brayne C Aarsland D Jones E Alzheimer's disease Lancet 2011 377 9770 1019 1031 10.1016/S0140-6736(10)61349-9 21371747

[B6] Li MD Burmeister M New insights into the genetics of addiction Nature reviews 2009 10 4 225 231 10.1038/nrg2536 19238175 PMC2879628

[B7] Bierut LJ Genetic vulnerability and susceptibility to substance dependence Neuron 2011 69 4 618 627 10.1016/j.neuron.2011.02.015 21338875 PMC3095110

[B8] Manolio TA Brooks LD Collins FS A HapMap harvest of insights into the genetics of common disease The Journal of clinical investigation 2008 118 5 1590 1605 10.1172/JCI34772 18451988 PMC2336881

[B9] Epstein DJ Cis-regulatory mutations in human disease Briefings in functional genomics & proteomics 2009 8 4 310 316 10.1093/bfgp/elp021 19641089 PMC2742803

[B10] Cookson W Liang L Abecasis G Moffatt M Lathrop M Mapping complex disease traits with global gene expression Nature reviews 2009 10 3 184 194 10.1038/nrg2537 19223927 PMC4550035

[B11] Pastinen T Genome-wide allele-specific analysis: insights into regulatory variation Nature reviews 2010 11 8 533 538 20567245 10.1038/nrg2815

[B12] Cheung VG Spielman RS Ewens KG Weber TM Morley M Burdick JT Mapping determinants of human gene expression by regional and genome-wide association Nature 2005 437 7063 1365 1369 10.1038/nature04244 16251966 PMC3005311

[B13] Kwan T Benovoy D Dias C Gurd S Provencher C Beaulieu P Hudson TJ Sladek R Majewski J Genome-wide analysis of transcript isoform variation in humans Nature genetics 2008 40 2 225 231 10.1038/ng.2007.57 18193047

[B14] Stranger BE Nica AC Forrest MS Dimas A Bird CP Beazley C Ingle CE Dunning M Flicek P Koller D Population genomics of human gene expression Nature genetics 2007 39 10 1217 1224 10.1038/ng2142 17873874 PMC2683249

[B15] Cheung VG Spielman RS Genetics of human gene expression: mapping DNA variants that influence gene expression Nature reviews 2009 10 9 595 604 10.1038/nrg2630 19636342 PMC2989458

[B16] Myers AJ Gibbs JR Webster JA Rohrer K Zhao A Marlowe L Kaleem M Leung D Bryden L Nath P A survey of genetic human cortical gene expression Nature genetics 2007 39 12 1494 1499 10.1038/ng.2007.16 17982457

[B17] Webster JA Gibbs JR Clarke J Ray M Zhang W Holmans P Rohrer K Zhao A Marlowe L Kaleem M Genetic control of human brain transcript expression in Alzheimer disease American journal of human genetics 2009 84 4 445 458 10.1016/j.ajhg.2009.03.011 19361613 PMC2667989

[B18] Liu C Cheng L Badner JA Zhang D Craig DW Redman M Gershon ES Whole-genome association mapping of gene expression in the human prefrontal cortex Molecular psychiatry 2010 15 8 779 784 10.1038/mp.2009.128 20351726 PMC3057235

[B19] Singer-Sam J Chapman V LeBon JM Riggs AD Parental imprinting studied by allele-specific primer extension after PCR: paternal X chromosome-linked genes are transcribed prior to preferential paternal X chromosome inactivation Proceedings of the National Academy of Sciences of the United States of America 1992 89 21 10469 10473 10.1073/pnas.89.21.10469 1279680 PMC50360

[B20] Yan H Yuan W Velculescu VE Vogelstein B Kinzler KW Allelic variation in human gene expression Science 2002 297 5584 1143 10.1126/science.1072545 12183620

[B21] Bray NJ O'Donovan MC Investigating cis-acting regulatory variation using assays of relative allelic expression Psychiatric genetics 2006 16 4 173 177 10.1097/01.ypg.0000218612.35139.84 16829785

[B22] Bray NJ Buckland PR Owen MJ O'Donovan MC Cis-acting variation in the expression of a high proportion of genes in human brain Human genetics 2003 113 2 149 153 12728311 10.1007/s00439-003-0956-y

[B23] Bray NJ Buckland PR Williams NM Williams HJ Norton N Owen MJ O'Donovan MC A haplotype implicated in schizophrenia susceptibility is associated with reduced COMT expression in human brain American journal of human genetics 2003 73 1 152 161 10.1086/376578 12802784 PMC1180576

[B24] Bray NJ Jehu L Moskvina V Buxbaum JD Dracheva S Haroutunian V Williams J Buckland PR Owen MJ O'Donovan MC Allelic expression of APOE in human brain: effects of epsilon status and promoter haplotypes Human molecular genetics 2004 13 22 2885 2892 10.1093/hmg/ddh299 15385439

[B25] Bray NJ Preece A Williams NM Moskvina V Buckland PR Owen MJ O'Donovan MC Haplotypes at the dystrobrevin binding protein 1 (DTNBP1) gene locus mediate risk for schizophrenia through reduced DTNBP1 expression Human molecular genetics 2005 14 14 1947 1954 10.1093/hmg/ddi199 15917270

[B26] Caffrey TM Joachim C Paracchini S Esiri MM Wade-Martins R Haplotype-specific expression of exon 10 at the human MAPT locus Human molecular genetics 2006 15 24 3529 3537 10.1093/hmg/ddl429 17085483

[B27] Pinsonneault JK Papp AC Sadee W Allelic mRNA expression of X-linked monoamine oxidase a (MAOA) in human brain: dissection of epigenetic and genetic factors Human molecular genetics 2006 15 17 2636 2649 10.1093/hmg/ddl192 16893905

[B28] Zhang Y Wang D Johnson AD Papp AC Sadee W Allelic expression imbalance of human mu opioid receptor (OPRM1) caused by variant A118G The Journal of biological chemistry 2005 280 38 32618 32624 10.1074/jbc.M504942200 16046395

[B29] Zhang Y Bertolino A Fazio L Blasi G Rampino A Romano R Lee ML Xiao T Papp A Wang D Polymorphisms in human dopamine D2 receptor gene affect gene expression, splicing, and neuronal activity during working memory Proceedings of the National Academy of Sciences of the United States of America 2007 104 51 20552 20557 10.1073/pnas.0707106104 18077373 PMC2154469

[B30] Lim JE Papp A Pinsonneault J Sadee W Saffen D Allelic expression of serotonin transporter (SERT) mRNA in human pons: lack of correlation with the polymorphism SERTLPR Molecular psychiatry 2006 11 7 649 662 10.1038/sj.mp.4001797 16432527

[B31] Lim JE Pinsonneault J Sadee W Saffen D Tryptophan hydroxylase 2 (TPH2) haplotypes predict levels of TPH2 mRNA expression in human pons Molecular psychiatry 2007 12 5 491 501 17453063 10.1038/sj.mp.4001923 PMC1896269

[B32] Yuferov V Ji F Nielsen DA Levran O Ho A Morgello S Shi R Ott J Kreek MJ A functional haplotype implicated in vulnerability to develop cocaine dependence is associated with reduced PDYN expression in human brain Neuropsychopharmacology 2009 34 5 1185 1197 10.1038/npp.2008.187 18923396 PMC2778041

[B33] Smith RM Alachkar H Papp AC Wang D Mash DC Wang JC Bierut LJ Sadee W Nicotinic alpha5 receptor subunit mRNA expression is associated with distant 5' upstream polymorphisms Eur J Hum Genet 2011 19 1 76 83 10.1038/ejhg.2010.120 20700147 PMC2995013

[B34] Pinsonneault JK Han DD Burdick KE Kataki M Bertolino A Malhotra AK Gu HH Sadee W Dopamine Transporter Gene Variant Affecting Expression in Human Brain is Associated with Bipolar Disorder Neuropsychopharmacology 2011 36 8 1644 1655 10.1038/npp.2011.45 21525861 PMC3138671

[B35] Ciobanu DC Lu L Mozhui K Wang X Jagalur M Morris JA Taylor WL Dietz K Simon P Williams RW Detection, validation, and downstream analysis of allelic variation in gene expression Genetics 2010 184 1 119 128 10.1534/genetics.109.107474 19884314 PMC2802080

[B36] Serre D Gurd S Ge B Sladek R Sinnett D Harmsen E Bibikova M Chudin E Barker DL Dickinson T Differential allelic expression in the human genome: a robust approach to identify genetic and epigenetic cis-acting mechanisms regulating gene expression PLoS genetics 2008 4 2 e1000006 10.1371/journal.pgen.1000006 18454203 PMC2265535

[B37] Ge B Pokholok DK Kwan T Grundberg E Morcos L Verlaan DJ Le J Koka V Lam KC Gagne V Global patterns of cis variation in human cells revealed by high-density allelic expression analysis Nature genetics 2009 41 11 1216 1222 10.1038/ng.473 19838192

[B38] Daelemans C Ritchie ME Smits G Abu-Amero S Sudbery IM Forrest MS Campino S Clark TG Stanier P Kwiatkowski D High-throughput analysis of candidate imprinted genes and allele-specific gene expression in the human term placenta BMC genetics 2010 11 25 20403199 10.1186/1471-2156-11-25 PMC2871261

[B39] Morcos L Ge B Koka V Lam KC Pokholok DK Gunderson KL Montpetit A Verlaan DJ Pastinen T Genome-wide assessment of imprinted expression in human cells Genome biology 2011 12 3 R25 10.1186/gb-2011-12-3-r25 21418647 PMC3129675

[B40] Main BJ Bickel RD McIntyre LM Graze RM Calabrese PP Nuzhdin SV Allele-specific expression assays using Solexa BMC genomics 2009 10 422 10.1186/1471-2164-10-422 19740431 PMC2749874

[B41] Zhang K Li JB Gao Y Egli D Xie B Deng J Li Z Lee JH Aach J Leproust EM Digital RNA allelotyping reveals tissue-specific and allele-specific gene expression in human Nature methods 2009 6 8 613 618 10.1038/nmeth.1357 19620972 PMC2742772

[B42] Lee JH Park IH Gao Y Li JB Li Z Daley GQ Zhang K Church GM A robust approach to identifying tissue-specific gene expression regulatory variants using personalized human induced pluripotent stem cells PLoS genetics 2009 5 11 e1000718 10.1371/journal.pgen.1000718 19911041 PMC2766639

[B43] Mortazavi A Williams BA McCue K Schaeffer L Wold B Mapping and quantifying mammalian transcriptomes by RNA-Seq Nature methods 2008 5 7 621 628 10.1038/nmeth.1226 18516045 PMC13303166

[B44] Wang Z Gerstein M Snyder M RNA-Seq: a revolutionary tool for transcriptomics Nature reviews 2009 10 1 57 63 10.1038/nrg2484 19015660 PMC2949280

[B45] Verlaan DJ Ge B Grundberg E Hoberman R Lam KC Koka V Dias J Gurd S Martin NW Mallmin H Targeted screening of cis-regulatory variation in human haplotypes Genome research 2009 19 1 118 127 18971308 10.1101/gr.084798.108 PMC2612965

[B46] Pickrell JK Marioni JC Pai AA Degner JF Engelhardt BE Nkadori E Veyrieras JB Stephens M Gilad Y Pritchard JK Understanding mechanisms underlying human gene expression variation with RNA sequencing Nature 2010 464 7289 768 772 10.1038/nature08872 20220758 PMC3089435

[B47] Babak T Garrett-Engele P Armour CD Raymond CK Keller MP Chen R Rohl CA Johnson JM Attie AD Fraser HB Genetic validation of whole-transcriptome sequencing for mapping expression affected by cis-regulatory variation BMC genomics 2010 11 473 10.1186/1471-2164-11-473 20707912 PMC3091669

[B48] Fontanillas P Landry CR Wittkopp PJ Russ C Gruber JD Nusbaum C Hartl DL Key considerations for measuring allelic expression on a genomic scale using high-throughput sequencing Molecular ecology 2011 19 Suppl 1 212 227 10.1111/j.1365-294X.2010.04472.x PMC3217793 20331781

[B49] Heap GA Yang JH Downes K Healy BC Hunt KA Bockett N Franke L Dubois PC Mein CA Dobson RJ Genome-wide analysis of allelic expression imbalance in human primary cells by high-throughput transcriptome resequencing Human molecular genetics 2010 19 1 122 134 10.1093/hmg/ddp473 19825846 PMC2792152

[B50] Williams NM Glaser B Norton N Williams H Pierce T Moskvina V Monks S Del Favero J Goossens D Rujescu D Strong evidence that GNB1L is associated with schizophrenia Human molecular genetics 2008 17 4 555 566 18003636 10.1093/hmg/ddm330

[B51] Maes OC Schipper HM Chong G Chertkow HM Wang E A GSTM3 polymorphism associated with an etiopathogenetic mechanism in Alzheimer disease Neurobiology of aging 2010 31 1 34 45 10.1016/j.neurobiolaging.2008.03.007 18423940

[B52] Lo HS Wang Z Hu Y Yang HH Gere S Buetow KH Lee MP Allelic variation in gene expression is common in the human genome Genome research 2003 13 8 1855 1862 12902379 10.1101/gr.1006603 PMC403776

[B53] Pastinen T Sladek R Gurd S Sammak A Ge B Lepage P Lavergne K Villeneuve A Gaudin T Brandstrom H A survey of genetic and epigenetic variation affecting human gene expression Physiological genomics 2004 16 2 184 193 14583597 10.1152/physiolgenomics.00163.2003

[B54] Johnson AD Zhang Y Papp AC Pinsonneault JK Lim JE Saffen D Dai Z Wang D Sadee W Polymorphisms affecting gene transcription and mRNA processing in pharmacogenetic candidate genes: detection through allelic expression imbalance in human target tissues Pharmacogenetics and genomics 2008 18 9 781 791 10.1097/FPC.0b013e3283050107 18698231 PMC2779843

[B55] Richards AL Jones L Moskvina V Kirov G Gejman PV Levinson DF Sanders AR Purcell S Visscher PM Craddock N Schizophrenia susceptibility alleles are enriched for alleles that affect gene expression in adult human brain Molecular psychiatry in press 10.1038/mp.2011.11 PMC4761872 21339752

[B56] Invernizzi RW Role of TPH-2 in brain function: news from behavioral and pharmacologic studies Journal of neuroscience research 2007 85 14 3030 3035 10.1002/jnr.21330 17492791

[B57] Haass C Selkoe DJ Soluble protein oligomers in neurodegeneration: lessons from the Alzheimer's amyloid beta-peptide Nat Rev Mol Cell Biol 2007 8 2 101 112 10.1038/nrm2101 17245412

[B58] Singleton A Myers A Hardy J The law of mass action applied to neurodegenerative disease: a hypothesis concerning the etiology and pathogenesis of complex diseases Human molecular genetics 2004 13 Spec No 1 R123 126 14976159 10.1093/hmg/ddh093

[B59] Brouwers N Sleegers K Engelborghs S Bogaerts V Serneels S Kamali K Corsmit E De Leenheir E Martin JJ De Deyn PP Genetic risk and transcriptional variability of amyloid precursor protein in Alzheimer's disease Brain 2006 129 Pt 11 2984 2991 16931535 10.1093/brain/awl212

[B60] Horan M Millar DS Hedderich J Lewis G Newsway V Mo N Fryklund L Procter AM Krawczak M Cooper DN Human growth hormone 1 (GH1) gene expression: complex haplotype-dependent influence of polymorphic variation in the proximal promoter and locus control region Human mutation 2003 21 4 408 423 10.1002/humu.10167 12655556

[B61] Tao H Cox DR Frazer KA Allele-specific KRT1 expression is a complex trait PLoS genetics 2006 2 6 e93 10.1371/journal.pgen.0020093 16789827 PMC1475705

[B62] Babbitt CC Silverman JS Haygood R Reininga JM Rockman MV Wray GA Multiple Functional Variants in cis Modulate PDYN Expression Molecular biology and evolution 2010 27 2 465 479 10.1093/molbev/msp276 19910384

[B63] Buonocore F Hill MJ Campbell CD Oladimeji PB Jeffries AR Troakes C Hortobagyi T Williams BP Cooper JD Bray NJ Effects of cis-regulatory variation differ across regions of the adult human brain Human molecular genetics 2010 19 22 4490 4496 10.1093/hmg/ddq380 20829226 PMC3298852

[B64] Kwan T Benovoy D Dias C Gurd S Serre D Zuzan H Clark TA Schweitzer A Staples MK Wang H Heritability of alternative splicing in the human genome Genome research 2007 17 8 1210 1218 10.1101/gr.6281007 17671095 PMC1933514

[B65] Lalonde E Ha KC Wang Z Bemmo A Kleinman CL Kwan T Pastinen T Majewski J RNA sequencing reveals the role of splicing polymorphisms in regulating human gene expression Genome research 2010 21 4 545 554 21173033 10.1101/gr.111211.110 PMC3065702

[B66] McDaniell R Lee BK Song L Liu Z Boyle AP Erdos MR Scott LJ Morken MA Kucera KS Battenhouse A Heritable individual-specific and allele-specific chromatin signatures in humans Science 2010 328 5975 235 239 10.1126/science.1184655 20299549 PMC2929018

[B67] Tycko B Allele-specific DNA methylation: beyond imprinting Human molecular genetics 2010 19 R2 R210 220 10.1093/hmg/ddq376 20855472 PMC2953749

[B68] Craig DW Pearson JV Szelinger S Sekar A Redman M Corneveaux JJ Pawlowski TL Laub T Nunn G Stephan DA Identification of genetic variants using bar-coded multiplexed sequencing Nature methods 2008 5 10 887 893 10.1038/nmeth.1251 18794863 PMC3171277

[B69] Edgar RC MUSCLE: multiple sequence alignment with high accuracy and high throughput Nucleic acids research 2004 32 5 1792 1797 10.1093/nar/gkh340 15034147 PMC390337

