# Supplementary figures and images for: Next-generation DNA sequencing-based assay for measuring allelic expression imbalance (AEI) of candidate neuropsychiatric disorder genes in human brain
Source: BMC Genomics. 2011 Oct 20;12:518. doi: 10.1186/1471-2164-12-518 (PMC3228908; doi:10.1186/1471-2164-12-518)

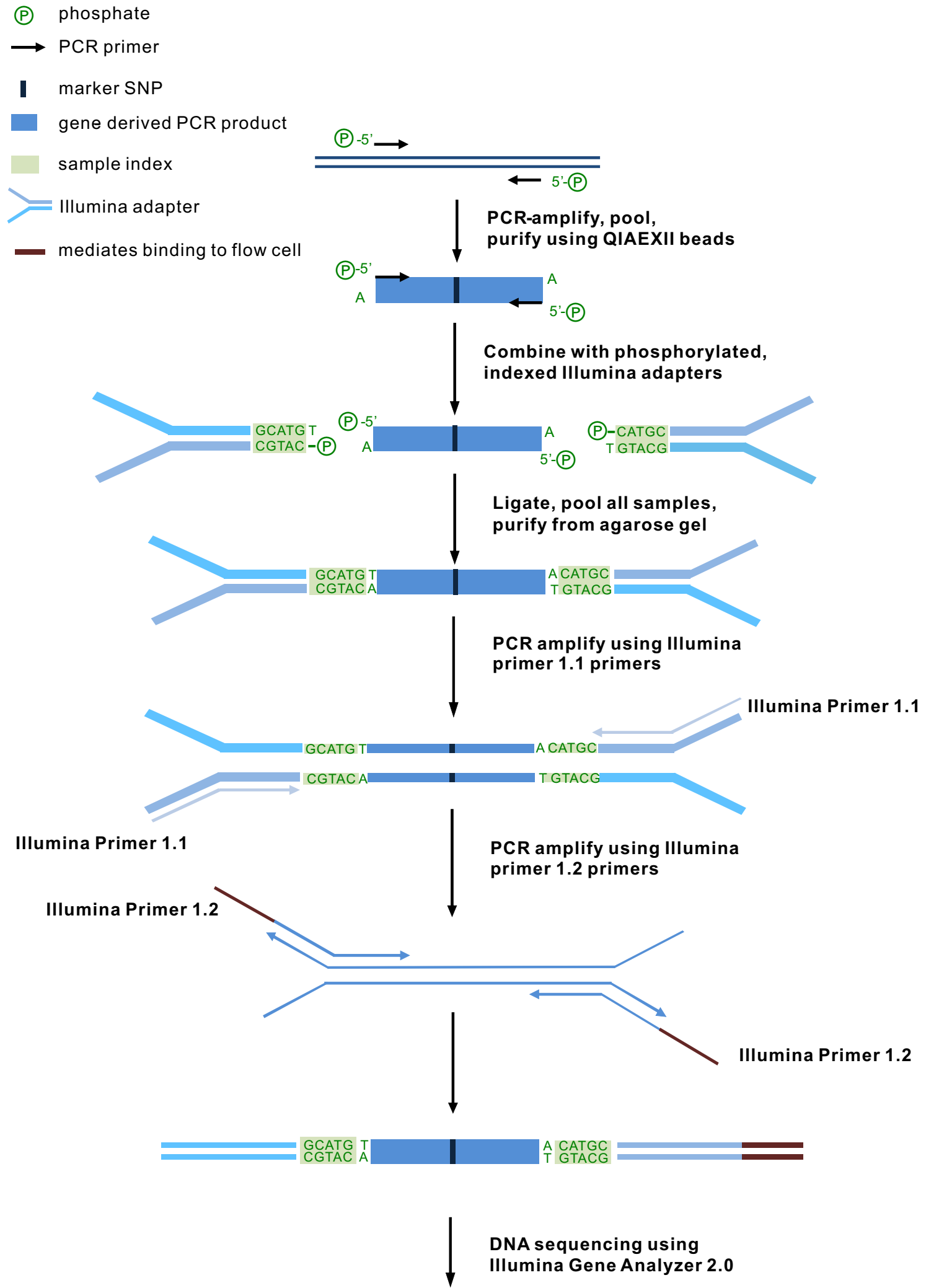

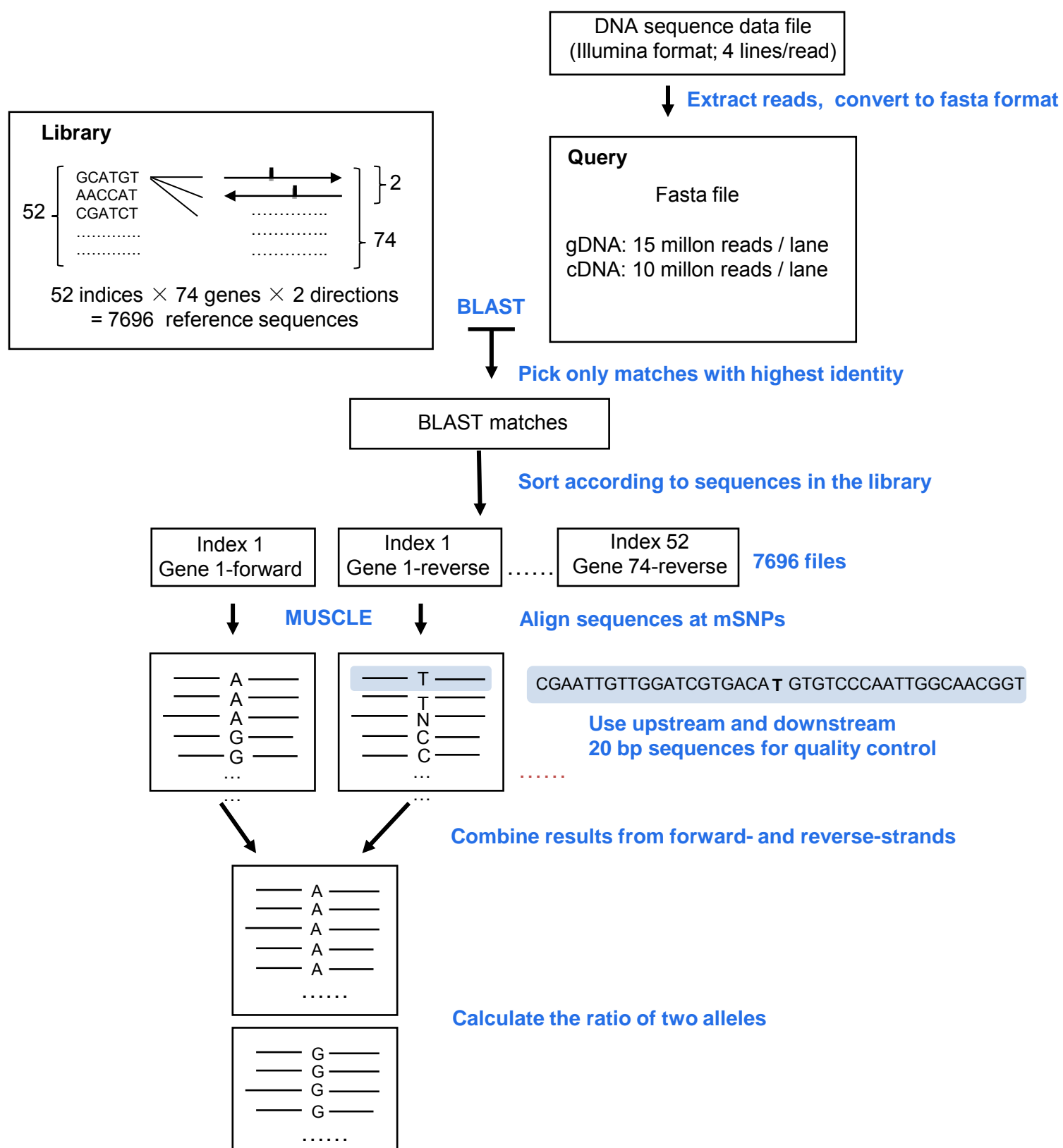

**a**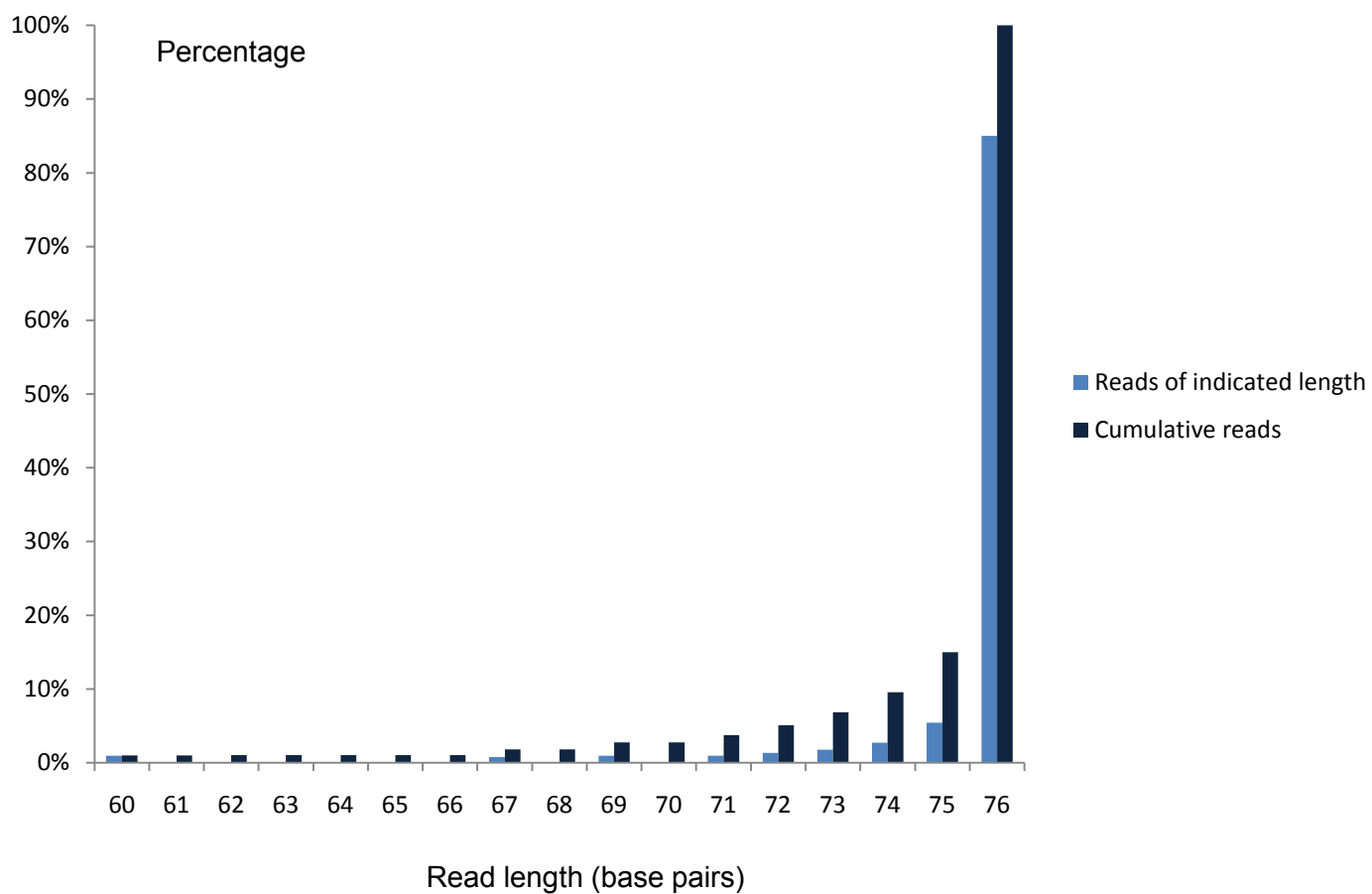**b**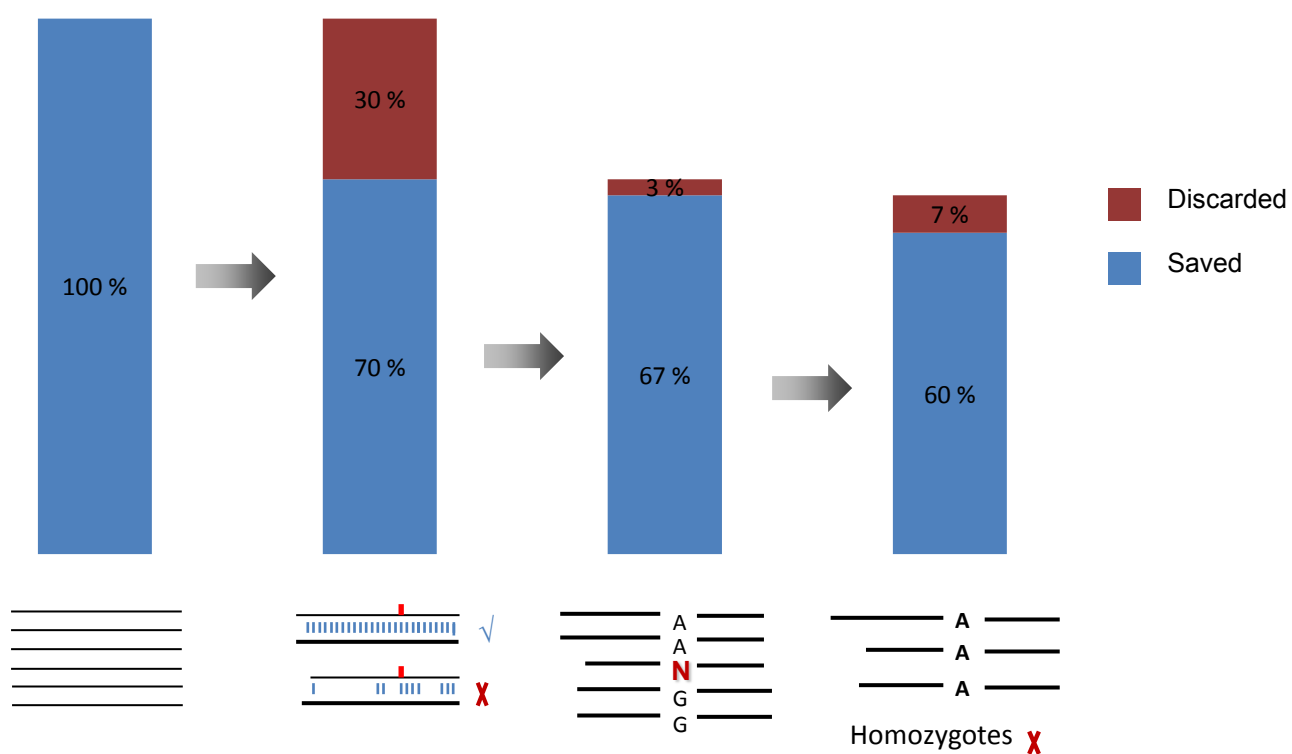

**a****Genomic DNA**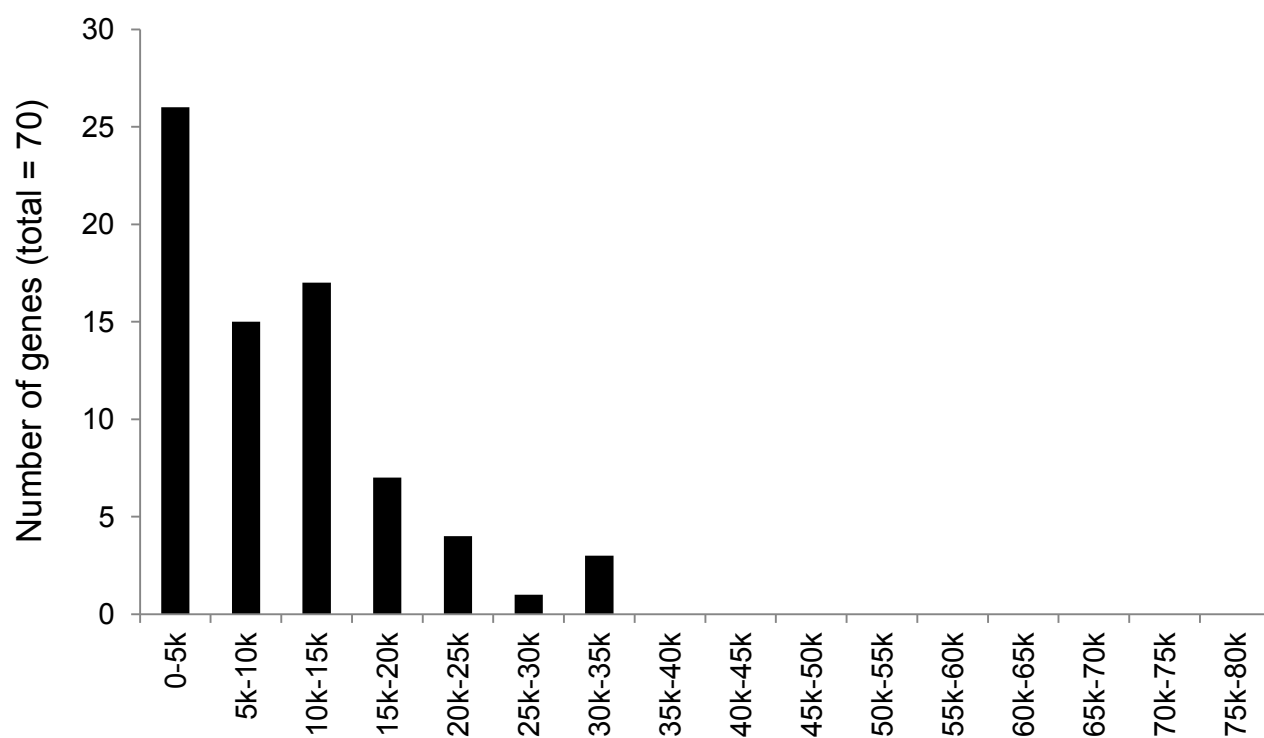**b****cDNA**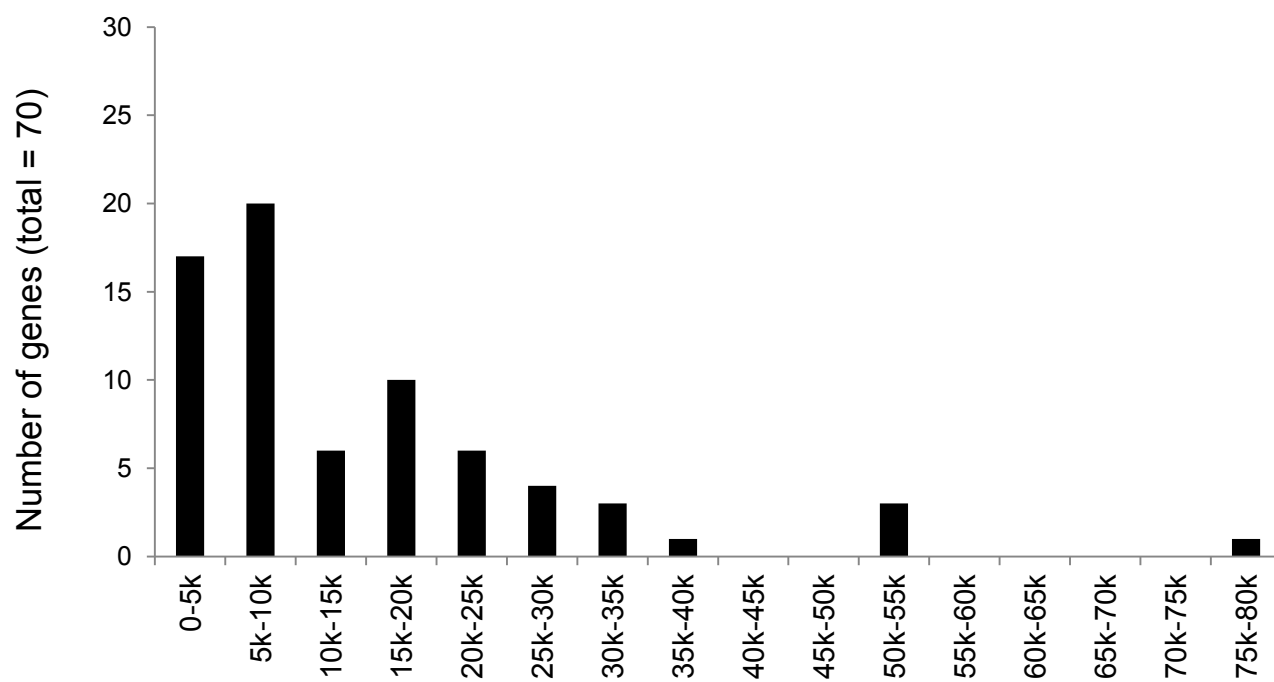

**a**

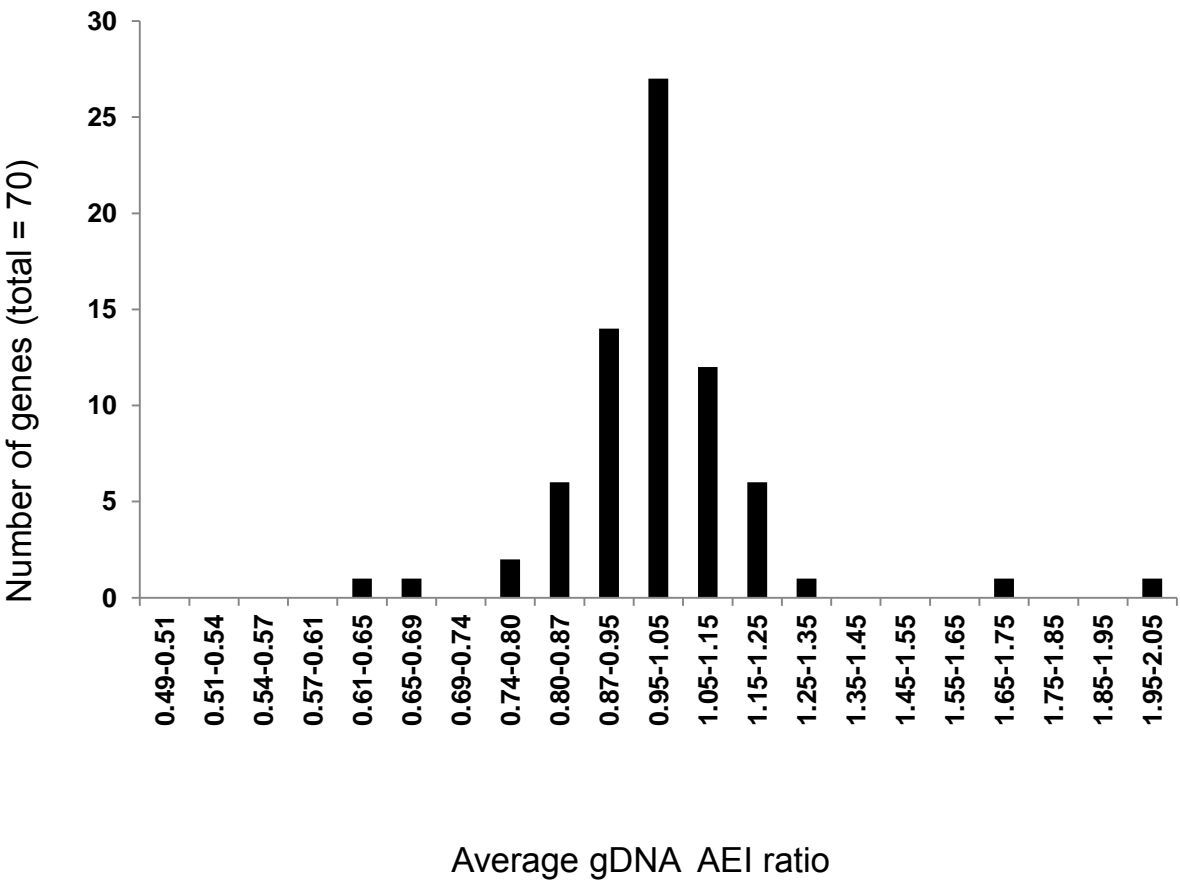

**b**

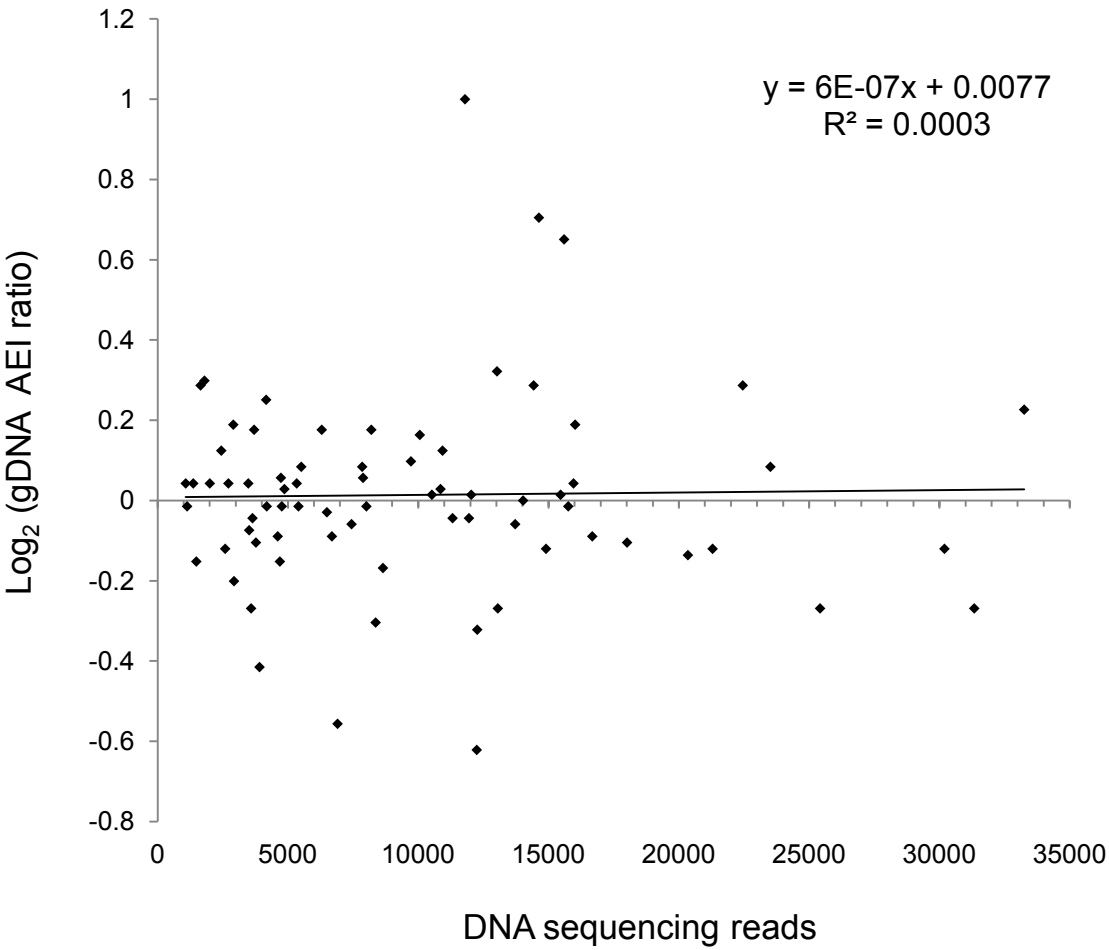

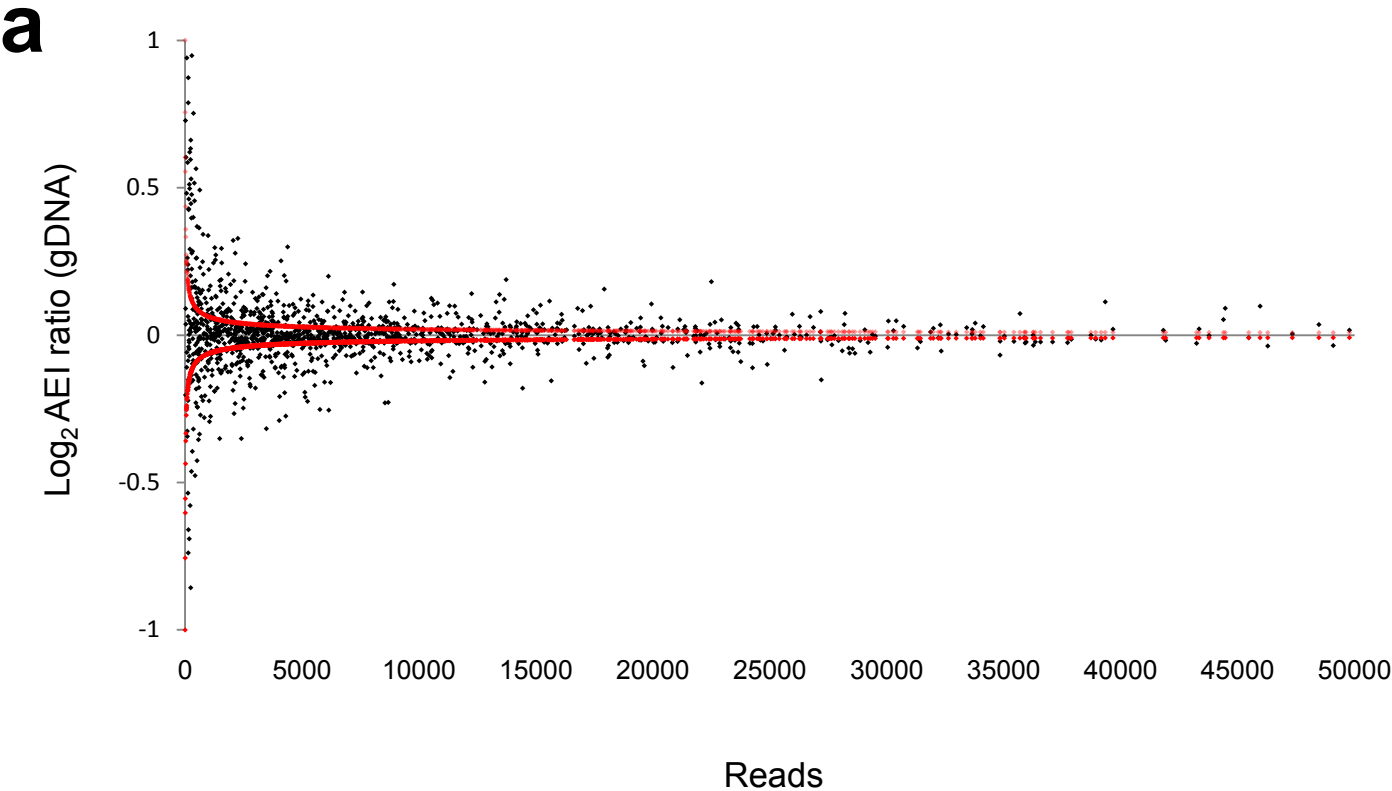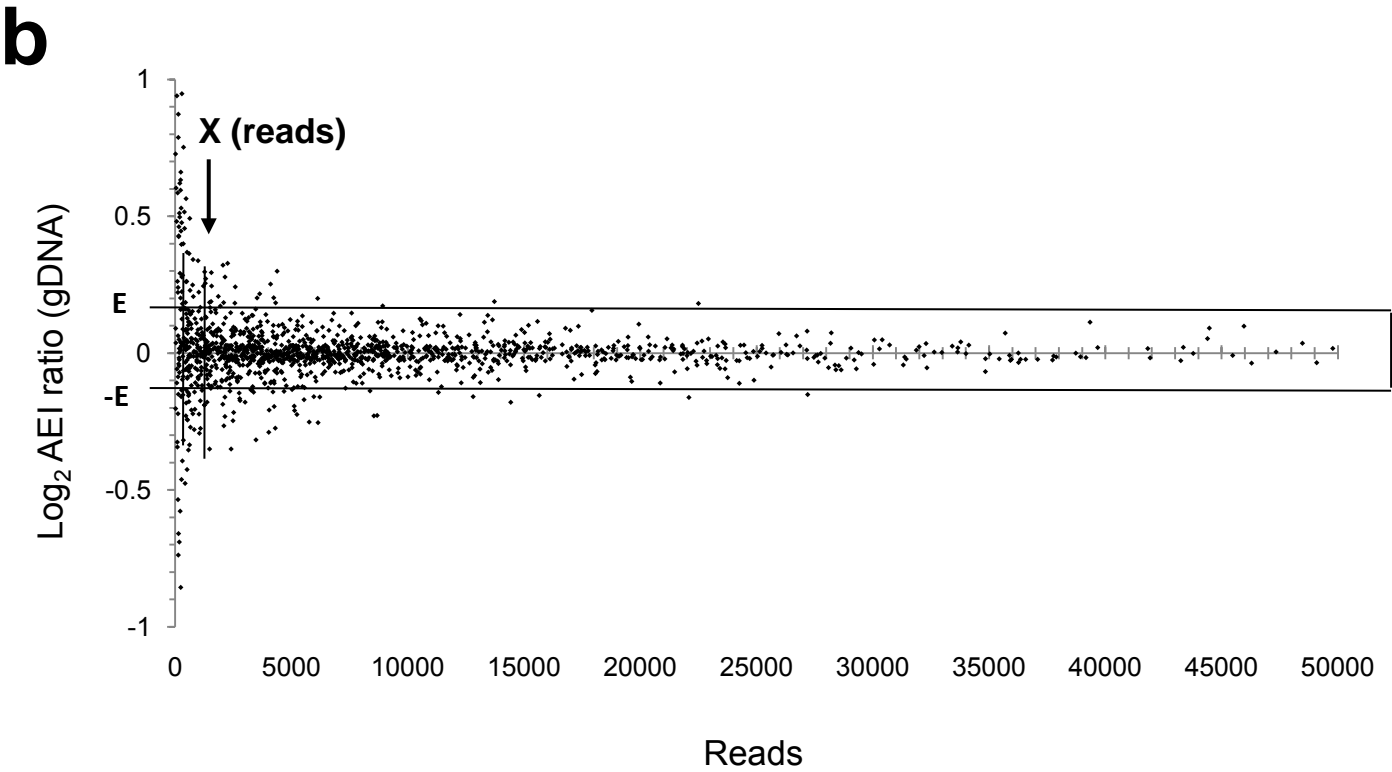

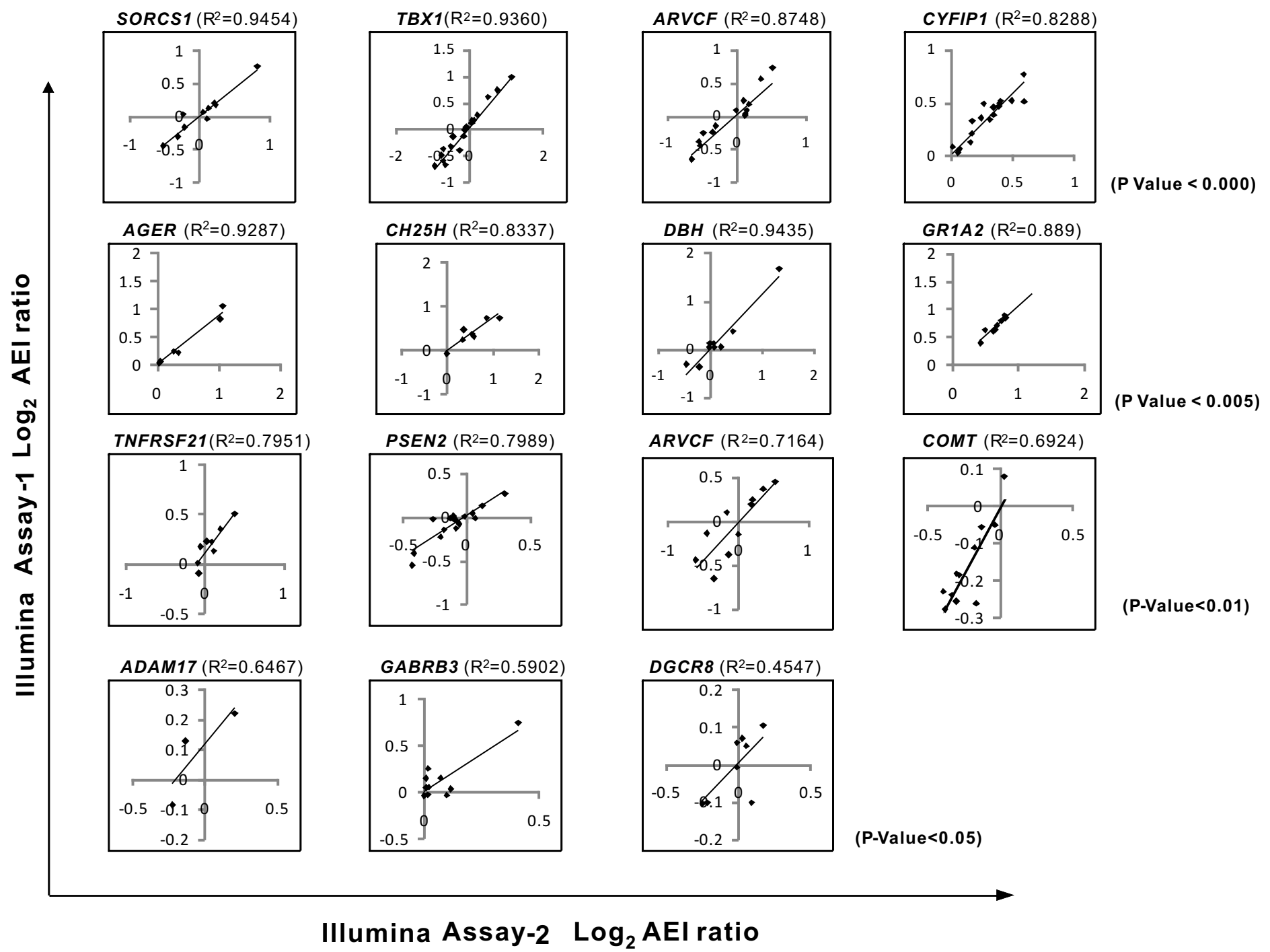

Supplement: Additional file 1 — Supplementary figures S1 - S7. Supplemental figures related to our next-generation DNA sequencing-based AEI assay Figure S1 - Preparation of samples for DNA sequencing. This diagram shows molecular details involved in preparing DNA samples for sequencing using the Illumina Genome Analyzer 2.0. Figure S2 - Flow diagram for sorting DNA sequence reads. This figure outlines the steps carried out by a computer program developed in- house for the calculation of AEI ratios using data produced by Illumina sequencing. Figure S3 - Percentage usable sequences. (a) Histogram of sequencing read lengths. Approximately 85% of the sequences were 76 bp in length following 76 sequencing cycles. (b) Among approximately 52.8 × 106 sequencing reads, 30% failed to meet match criteria in the BLAST step and were discarded. Another 3% were discarded due to missing sequence data for the marker SNP. Finally, 7% of the reads yielded sequences containing only one of the two mSNP alleles (reflecting genotyping errors or mistaken genotype imputation for the mSNP in some samples), yielding approximately 31.7 × 106 reads suitable for calculating AEI ratios. Figure S4 - Distribution of sequencing read numbers used for the calculation of gDNA- and cDNA-based AEI ratios. Histograms showing the distribution of (a) 13.2 × 106 gDNA reads and (b) 18.5 × 106 cDNA reads (mSNP M-allele + m-allele) among the 70 candidate genes in this study. (Data from Additional file 2, Table S5) Figure S5 - Distribution of non-corrected gDNA-based AEI ratios (a) Distribution of experimentally determined gDNA AEI ratios. The ideal AEI ratio for heterozygous samples = 1. (b) Regression analysis show that there is no correlation between sequencing read number and calculated gDNA ratios. Figure S6 - Error analysis (a) Plot of gDNA log2AEI ratios vs number of sequencing reads with super-imposed plot of the theoretical binominal sampling distribution (red trace), which was calculated based upon the assumption that the M- and m-al [file 1471-2164-12-518-S1.PDF]
