# Supplementary material for: Next-generation DNA sequencing-based assay for measuring allelic expression imbalance (AEI) of candidate neuropsychiatric disorder genes in human brain
Source: BMC Genomics. 2011 Oct 20;12:518. doi: 10.1186/1471-2164-12-518 (PMC3228908; doi:10.1186/1471-2164-12-518)
Supplement: Additional file 2 — Supplementary tables S1 - S6. Detailed information concerning our candidate neuropsychiatric disorder genes, PCR and sequencing primers, experimental error and measured AEI ratios. Table S1 - Neuropsychiatric disorder candidate genes. Table S2 - PCR primer sequences. Table S3 - Index sequences. Table S4 - Estimation of Experimental Error. Table S5 - Data for AEI ratio measurements (Illumina Assay-2) Table S6 - SNPs within PCR primer binding sites. [file 1471-2164-12-518-S2.PDF]

Additional File 2 - Supplementary Tables

Table S1 - Neuropsychiatric disorder candidate genes

	Symbol	Encoded protein	Location	Disorder	mSNP; alleles*	Het**	Definition AEI ratio
1	*ADAM17*	ADAM metallopeptidase domain-17	2p25	AD	rs1048610: T>C	0.135	T/C
2	*AGER*	advanced glycosylation end product-specific receptor	6p21.3	AD	rs2070600: G>A	0.288	G/A
3	*APBA2*	amyloid beta precursor protein-binding, family A, member 2	15q11-12	SCZ	rs3751555:C>G	0.51	C/G
4	*ARRB2*	arrestin, beta 2	17p13	Addiction	rs1045280:T>C	0.21	T/C
5	*ARVCF*	armadillo repeat gene deleted in velocardiofacial syndrome	22q11.2	SCZ	rs165655: A>G	0.43	G/A
6	*APH1A*	anterior pharynx defective 1 homolog A	1p36.13-q31.3	AD	rs2275780: G>T	0.419	G/T
7	*BACE1*	beta-site APP-cleaving enzyme 1	11q23.2-q23.3	AD	rs535860: A>T	0.058	A/T
8	*BACE2*	beta-site APP-cleaving enzyme 2	21q22.3	AD	rs12149: T>C	0.308	T/C
9	*BDNF*	brain-derived neurotrophic factor	11p13	Addiction	rs6265: A>G	0.31	A/G
10	*CALHM1*	calcium homeostasis modulator 1	10q24.33	AD	rs729211: A>G	0.481	A/G
11	*CH25H*	cholesterol 25-hydroxylase	10q23	AD	rs4078488: A>G	0.327	A/G
12	*CLU*	clusterin	8p21-p12	AD	rs9331942: T>C	0.385	T/C
13	*CNR1*	cannabinoid receptor 1	6q14-q15	Addiction	rs806368: T>C	0.46	C/T
14	*CNTNAP2*	contactin associated protein-like 2	7q35-36	SCZ, ASD	rs2530311: C>T	0.4	T/C
15	*COMT*	catechol-O-methyltransferase	22q11.21	Addiction	rs165728: T>C	0.42	T/C
16	*CYFIP1*	cytoplasmic FMR1 interacting protein 1	15q11.2	SCZ, ASD	rs2289818: G>C	0.49	C/G
17	*CYP2D6*	cytochrome P450, family 2, subfamily D, polypeptide 6	22q13.1	Addiction	rs1058164: C>G	0.56	C/G
18	*DBH*	dopamine beta-hydroxylase	9q34	Addiction	rs129882: C>T	0.69	C/T
19	*DGCR14*	DiGeorge syndrome critical region gene 14	22q11.2	SCZ	rs1052763: C>T	0.36	C/T
20	*DGCR8*	DiGeorge syndrome critical region gene 8	22q11.2	SCZ	rs1640299: T>G	0.36	G/T
21	*DISC1*	disrupted in schizophrenia 1	1q42.1	SCZ	rs9729179: T>C	0.53	C/T
22	*DLG4*	Discs, large homolog 4	17p13.1	SCZ	rs17203281:G>A	0.42	G/A
23	*DRD1*	Dopamine receptor D1	5q35.1	Addiction	rs4867798: T>C	0.46	T/C
24	*DRD2*	Dopamine receptor D2	11q23	Addiction	rs6279: G>C	0.42	C/G
25	*DRD3*	Dopamine receptor D3	3q13.3	Addiction	rs6280: T>C	0.13	T/C
26	*ERBB4*	v-erb-a erythroblastic leukemia viral oncogene homolog 4	2q34	SCZ	rs16845990: T>C	0.533	T/C
27	*FosB*	FBJ murine osteosarcoma viral oncogene homolog B	19q13.32	Addiction	rs1049739: A>G	0.5	A/G
28	*GAB2*	GRB2-associated binding protein 2	11q14.1	AD	rs1046780: T>C	0.442	T/C
29	*GABRB3*	gamma-amino-butyric acid A receptor, subunit beta 3	15q11.2	SCZ	rs2017247: A>G	0.511	A/G
30	*GABRG3*	gamma-aminobutyric acid A receptor, gamma 3	15q11.2	SCZ	rs140679: T>C	0.5	C/T

31	*GAL*	galanin prepropeptide	11q13.3	Addiction	rs1042577: G>A	0.27	G/A
32	*GNB1L*	**guanine nucleotide binding protein beta polypeptide 1-like**	22q11.2	SCZ, BP	rs2073770: C>A	0.467	A/C
33	*GRIA2*	glutamate receptor, ionotropic, AMPA 2	4q32-q33	Addiction	rs6855973: A>T	0.33	A/T
34	*GRIN2A*	glutamate receptor, ionotropic, N-methyl D-aspartate 2A	16p13.2	Addiction	rs1420040: A>G	0.37	A/G
35	*GRIN2B*	glutamate receptor, ionotropic, N-methyl D-aspartate 2B	12p12	Addiction	rs890: T>G	0.27	G/T
36	*GRM3*	glutamate receptor, metabotropic 3	7q21.1-q21.2	Addiction	rs2228595: C>T	0.13	C/T
37	*GRN*	granulin	17q21.32	FTD	rs5848: G>A	0.404	G/A
38	*GSK3B*	glycogen synthase kinase 3 beta	3q13.3	SCZ	rs3732361: A>G	0.4	A/G
39	*HTR1B*	5-hydroxytryptamine (serotonin) receptor 1B	6q13	Addiction	rs6298: T>C	0.37	C/T
40	*HTR2A*	5-hydroxytryptamine (serotonin) receptor 2A	13q14-q21	Addiction	rs6313: C>T	0.42	C/T
41	*HTR3B*	5-hydroxytryptamine (serotonin) receptor 3B	11q23.1	Addiction	rs2276305: G>A	0.38	G/A
42	*LRP1*	low density lipoprotein receptor-related protein 1	12q13-q14	AD	rs1140648: A>G	0.423	A/G
43	*LRRK2*	leucine-rich repeat kinase 2	12q12	PD	rs10784548: T>C	0.538	T/C
44	*MAPT*	microtubule-associated protein tau	17q21.1	AD	rs7521: G>A	0.154	G/A
45	*MCHR1*	melanin-concentrating hormone receptor 1	2p25.3	SCZ	rs133074: C>T	0.122	C/T
46	*MYT1L*	myelin transcription factor 1-like	2p25.3	SCZ	rs17338616:G>A	0.444	A/G
47	*NDE1*	nudE nuclear distribution gene E homolog 1	16p13.1	SCZ	rs2075511: T>G	0.451	T/G
48	*NGFR*	nerve growth factor receptor	17q21-q22	Addiction	rs741071: C>T	0.25	C/T
49	*NPY*	neuropeptide Y	7p15.1	Addiction	rs5574: C>T	0.25	C/T
50	*NRXN1*	neurexin 1	2p16.3	SCZ	rs11885824:C>A	0.4	C/A
51	*NTAN1*	N-terminal Asn amidase	16p13.1	SCZ	rs1136001: G>T	0.429	G/T
52	*NTRK2*	Neurotrophic tyrosine kinase, receptor, type 2	9q22.1	Addiction	rs3739570: C>T	0.48	C/T
53	*OPRD1*	opioid receptor, delta 1	1p36.1-p34.3	Addiction	rs4654327: A>G	0.38	A/G
54	*OPRK1*	opioid receptor, kappa 1	8q11.2	Addiction	rs1051660: G>A	0.23	G/A
55	*OPRM1*	opioid receptor, mu 1	6q24-q25	Addiction	rs1799971: A>G	0.38	A/G
56	*P25*	tubulin polymerization promoting protein	5p15.3	AD	rs1697963: T=C	0.442	T/C
57	*PDYN*	prodynorphin	20p13	Addiction	rs2235749: A>G	0.19	A/G
58	*PICALM*	phosphatidylinositol binding clathrin assembly protein	11q14	AD	rs694353: G>T	0.481	G/T
59	*PRODH*	proline dehydrogenase (oxidase) 1	22q11.2	SCZ	rs1808320: G>A	0.422	G/A
60	*PRRG4*	proline rich Gla (G-carboxyglutamic acid) 4	11p12-13	SCZ	rs7933966: G=A	0.495	A/G
61	*PSEN1*	presenilin 1	14q24.3	AD	rs7523: G>A	0.192	G/A
62	*PSEN2*	presenilin 2	1q31-q42	AD	rs1046240: C>T	0.481	C/T
63	*RAI1*	retinoic acid induced 1	17p11.2	SCZ	rs11649804:A>C	0.244	A/C
64	*RGS4*	regulator of G-protein signaling 4	1q21	SCZ	rs10759: A>C	0.4	A/C
65	*SLC1A2*	solute carrier family 1	11p12-13	SCZ	rs10768122:G>A	0.45	G/A
66	*SLC6A2*	solute carrier family 6，member 2	16q12.2	Addiction	rs5569: C>T	0.36	C/T
67	*SLC6A3*	solute carrier family 6, member 3	5p15.3	Addiction	rs1042098: T>C	0.15	T/C

68	*SORCS1*	sortilin-related VPS10 domain containing receptor 1	10q23-q25	AD	rs3802553: T>C	0.385	T/C
69	*SORL1*	sortilin-related receptor, L(DLR class) A repeats containing	11q23.2-q24.2	AD	rs12364988: T>C	0.385	T/C
70	*TBX1*	T-box 1	22q11.2	SCZ	rs41298814: T>C	0.58	C/T
71	*TNFRSF1*	tumor necrosis factor receptor superfamily, member 1A	12p13.2	AD	rs767455: T>C	0.25	T/C
72	*TNFRSF21*	tumor necrosis factor receptor superfamily, member 21	6p21.1	AD	rs6458555: A>G	0.423	A/G
73	*TNK1*	tyrosine kinase, non-receptor, 1	17p13.1	AD	rs2075760: C>T	0.365	C/T
74	*ZNF804A*	Zinc finger protein 804A	2q32.1	SCZ	rs12476147: T>A	0.381	A/T

(*) Data for HapMap-HCB population in the NCBI SNP database, except for genes 5, 11, 16, 17, 23, 54 and 70, for which the major and minor allele frequencies were based on genotyping results for the HapMap CHB+JPT populations (NCBI) or our set of brain samples; (**) Heterozygosity in our collection of 52 brain samples (Han Chinese)

Table S2 - PCR primer sequences

Gene Symbol	SNP	Forward Primer (5' to 3')	Reverse Primer (5' to 3')	Length of PCR product (bp)
ADAM17	rs1048610	TGAAACTGACAACTCCTGCA	AGTTCTTTTGTTCAGCATCG	83
AGER	rs2070600	TGGGACAGTGTGGCTCGTGT	CCTCATCCTGGATCCCGACA	70
APBA2	rs3751555	CCTCTCCCGTCGTCTCTTCT	AGGGGGCAGACACCTGTG	95
ARRB2	rs1045280	GCTCCACATTCTGTAAGGTG	CTTGAGTTTCCCATCCAGG	89
ARVCF	rs165655	TGCAGGCAGGCAGACCAC	CCCACCTTGCTGCTTCCA	82
APH1A	rs2275780	CCCTCCCATTTGCCTGTCCT	AGCCGAAAAACACCGCAGCC	81
BACE1	rs535860	GCATCACACGCAGGTTACCT	GACTTTGGCCAGCAGGGAAA	86
BACE2	rs12149	CAAAACAGAGTGGATTGGGC	TGTTGGTGGCACATGTAGAC	72
BDNF	rs6265	GCAGGTTCAAGAGGCTTGACA	CTTTCTGGTCCTCATCCAACA	79
CALHM1	rs729211	CCCCCAAACCAGGTGGAAAA	GGCAGGCGAGTGCTAGGGAG	86
CH25H	rs4078488	CCTTCCACAAGGTGCACCAC	AGAAAACAGTTCCCAGACGC	80
CLU	rs9331942	AAGGAGAAGAATCAGTAAGT	CACCAAATGTTTTCATAAC	80
CNR1	rs806368	TCAGATGTTTGAGCAGTGGC	GCGGAAGATAGCTTTTAAGATG	91
CNTNAP2	rs2530311	TAAATTGCTAGTCCCAGTAG	TGGTTCTTAGCTGAAGTCAG	90
COMT	rs165728	TCCCTGGCCCATGAGTGAG	TTGGAGGAGATGGGGACAGC	74
CYFIP1	rs2289818	ACAATGACAGCGCCCACT	ACATAGATTCACCTCGGCC	84
CYP2D6[1]	rs1058164	GCGCGAGCAGAGGCGCTTCT	ACCCACTGCTCCAGCGACTT	75
DBH	rs129882	ACTCTGCGACGATCCCCATG	CTCAGGCAGGGGCGTGGTCT	72

DGCR14	rs1052763	ACTCAGCGCGGTACTTGC	TGAGATCCTCAGCCACTCG	98
DGCR8	rs1640299	AGTTACCCTGGCCTCCTAGG	AGGAAAGGCAGAGAGGGC	93
DISC1	rs9729179	CCCAAGAGAAGCTCTCTAAA	GCAGATATTATGGTATGGTGTA	80
DLG4	rs17203281	CTGCCATTGCCCTGAAGA	GCCTCGAATCGGCTGTAC	82
DLG4 (G)	rs17203281	CCATGAGCAGGCTGCCATTG	ACTTCCGGCCTGGTACCTTC	85
DRD1	rs4867798	GGCGGTTTTGAACACAGTAGTAGC	GGCGGGCAAATGCTATAAATGCTT	88
DRD2	rs6279	CTAGGGTTGCTGGAGCCTGA	ACTGTCCATCTCTCCCCACC	93
DRD3	rs6280	CTCCCTCTGGGCTATGGCAT	GCTGGCACCTGTGGAGTTCT	85
ERBB4	rs16845990	ATTTTGGGCAGAAGTAAATG	GAGTATTTTCCAGAGGAAGC	99
FOSB	rs1049739	TGTATTTTGCATCTGACCCC	ACAGTGCAGAACCAAGGGGA	75
GAB2	rs1046780	TTTAGGACCTGGGACCAC	CACATGCCACCATAAATCAC	84
GABRB3	rs2017247	GCTGTCAGTGTGAGCCAT	CCAAGGCACACTAGCTAG	97
GABRG3	rs140679	CGTGTCCTACGTGACCGC	GTGGCATACTCCATCAGC	81
GAL	rs1042577	GCCTCCTCAGAAGACATCG	TTGACTTCAGGTTACAGCACA	75
GNB1L	rs2073770	ATCTCAGGCTCCGCGGGGAA	ATGAGTCCCACGCACCTGCA	78
GNB1L (G)	rs2073770	GGGGCATCTCAGGCTCCG	CCTGGCGCACTGACCTGC	82
GRIA2	rs6855973	GCTATTTATTGTACCTCTGG	TAGTTCAAGCTTGACAGAGA	75
GRIN2A	rs1420040	GCTAGTAAATCCCTAACG	TTCTATTCTGACCTCCCT	69
GRIN2B	rs890	GAACCAAAAAGAGGCTATGT	GCTTCCTCACCTAAATGAAA	72
GRM3	rs2228595	CGACGACTCGCGGGAGCTCA	CGTCGCTGGCCACCCAGGTG	71
GRN	rs5848	CGGGACCCCACTCGGA	CAGAATGGGGTCCAGGGAGA	82
GSK3B	rs3732361	TGAGAGAGTGACAGAGAATTG	ACCACTGACGTATCAAAACC	75
HTR1B	rs6298	TGCAGCGCCAAGGACTACATT	TGAGCGCCAATAGCATAACC	79
HTR2A	rs6313	CAGCTCTTGCATGCAGTTTT	CCCCTCTTCTTGATCTTC	81
HTR3B	rs2276305	CATCTGGGACCATTGAGA	GCAATTCTGGACATCAAATG	92
LRP1	rs1140648	CCGACTGGGAAACAAAGTCC	GTGCAGCGTGCTGATGAGGA	80
LRRK2	rs10784548	TAGAATTCCACAGCTCCTAC	CCATCATAACTAAAGCTCC	89
MAPT	rs7521	CTGCGTGTCCCATCTACAGA	CCCTTTTCAAAGCTGAAGAG	67
MCHR1	rs133074	TGAAAAATAAAGCATCCCATCTCTC	TCCCCTCAAATAAACATGCA	87
MYT1L	rs17338616	GACTTTGACTCTGAATCAGC	ATGCTCTATGGTCAACTG	98

NDE1	rs2075511	TCGTCCAGCTCCTGCTGAA	GAGATCGAGAACCTCACCCA	95
NGFR	rs741071	TCCTCCCCTTCCCTGCAAAC	CTTCTTGCCGCATTCCCACA	88
NPY	rs5574	ACATCAACCTCATCACCAG	CTGTGCTTTCTCTCATCAAG	87
NPY (G)	rs5574	CTATTCCAAACTTGCTTTA	CTTTCTCTCATCAAGAGG	96
NRXN1	rs11885824	AATGCATTATTACATTGC	ATCGTTCTTTTAGAAGGT	76
NTAN1[2]	rs1136001	TTAGATCTACCTTGATGTT	TGTAGAGTAGGGCTTTAT	81
NTRK2	rs3739570	TGTGCATGGTCTTCGTCGA	TGGATCTGCTCTACCTATCAGGG	84
OPRD1	rs4654327	GGCTCTACAACTGAGTCC	AGAAACCGAAGCTGTCTC	75
OPRK1	rs7820807	CCACTCTAACTGGGAGGA	CTCGAAGGATGCTCTTAGA	75
OPRM1	rs1799971	GGTTCCTGGGTCAACTTGTC	CAGGTCGGTGCGGTTC	72
P25	rs1697963	GCAAGGGCAAGGGCAAGGCT	GGTCGTAGGTGCCTGCGTGCT	84
PDYN	rs2235749	AGCTGAATTGGCTGCCAA	TTCTAGCTGCTGCTTCTGGA	88
PENK	rs3808633	GCTCCTGGCGACCGTG	GGCGCACTAGGCGGTAG	68
PICALM	rs694353	GGATCTAACTGGCAACCAAA	GGGGTGCGTATTGTGGAA	88
PICALM (G)	rs694353	CAACCAAAGGTTGCACCA	CAACTGTTAGTCTATCAGGAGC	80
PRODH	rs372055	CTCATGAAGGGCACCCAT	GAGGTTGCCAGTTCGGAG	75
PRRG4	rs11032025	CCCTCCATCATTTTCAGA	TAAGAAGGTAATCCTGCA	83
PSEN1	rs7523	CACAAGCAGTCTTTTTCTAC	CAAAGAGTAAGACCCTAGAA	83
PSEN2	rs1046240	AGGCCTGGAGGAAGAGCTGA	TCATGCACAGAGTGACAGGC	80
RAL1	rs11649804	CCCCCCAGCAGGCAGTATGCAG	CGGTGGCTGCTGGACGTGCA	78
RGS4	rs10759	AACTCACAGACTGCTGTCTT	CAACTGAAAAACACACTCAA	99
SLC1A2	rs1043101	GCAAATTAGTGGTAAATG	GCCTCATAGTTGGTTCTA	92
SLC6A2	rs5569	CCTTGACAGCTCAATGGG	CAAATGTGAAGAGTTTCCG	95
SLC6A2 (G)	rs5569	ATGGGAGGCATGGAGGCTGT	GAAGAGTTTCCGGTGTCGCT	75
SLC6A3	rs1042098	AAGGTGTAGAGGGAGCAGA	TCCTTGGTTTGTTCGTGT	74
SORCS1	rs3802553	CAATTAGAAGAGGGTGATGT	TGGAGCTGAGATTGACGTA	88
SORCS1(G)	rs3802553	GGTTGTTTTTCAGGGTGA	GGAGCTGAGATTGACGTAAG	88
SORL1	rs12364988	ATTGATCCCTATGACAAACC	CTGTACTTCGGAAGACAGTG	82
TBX1	rs41298814	GGCACCGAGATGATCGTCA	ATAGTCGGCCATGGGATCCA	90
TBX1(G)	rs41298814	TGCCTTCCACCAGCTAGGGT	GGGATCCATGCCGAAGAGCT	90

TNFRSF1	rs767455	CTGTCTGGCATGGGCCTCTC	CCCCTGAGGGGTATATTCCC	88
TNFRSF1(G)	rs767455	CATAGCTGTCTGGCATGG	CACTCTTCCCTTTGTCCCT	80
TNFRSF21	rs6458555	GTGGTTGGCAGTGGCTGGAA	AGGAGAACCAGGGAGGAGGA	82
TNK1	rs2075760	AAAAGAGCTGCTATACATCA	ATGGCTGTTCACATGGTC	87
ZNF804A	rs12476147	AGTAGATTTGTCCCCAGT	AGAGTTAGTTTTCTCCTCA	84
(G): Primers specifically designed for genotyping: alternative primers for the same gene span exon-exon junctions; (1) These primers also amplify a homologous DNA segment in the *CYP2D7p* pseudogene; (2) These primers also amplify a homologous DNA segment in *FAM82B*.				

Table S3 - Index sequences

Individual	Index		Individual	Index		Individual	Index
2008274	5'-GCATGT		2009325	5'-TCCTTT		2010056	5'-AGGAAT
2008281	5'-AACCAT		2009330	5'-TGACTT		2010066	5'-ACTGAT
2008295	5'-CGATCT		2009337	5'-AGAGAT		2010077	5'-ACGTAT
2008296	5'-CAACCT		2009338	5'-CATGCT		2010085	5'-TACGTT
2008298	5'-GGTTGT		200939	5'-CGCGCT		2010102	5'-CCTTCT
2008306	5'-AAGGAT		200950	5'-GCCGGT		2010106	5'-AGTTAT
2008323	5'-AGCTAT		200951	5'-GCGCGT		2010118	5'-AGCCAT
2009114	5'-ACACAT		200955	5'-GGCCGT		2010150	5'-ACGGAT
2009129	5'-AATTAT		200965	5'-GCTAGT		2010154	5'-ACTTAT
2009147	5'-TCTCTT		200977	5'-CCGGCT		2010159	5'-CTTCCT
2009149	5'-TCAGTT		200978	5'-GGAAGT		2010166	5'-GTTGGT
2009150	5'-TATATT		200981	5'-GACTGT		2010177	5'-GTACGT
2009156	5'-TCGATT		200997	5'-GAGAGT			
2009161	5'-TAGCTT		2010001	5'-CGGCCT			
2009166	5'-GATCGT		2010016	5'-CCAACT			
2009193	5'-TGCATT		2010022	5'-ACCAAT			
2009280	5'-AGTCAT		2010024	5'-CACACT			
2009290	5'-TGTGTT		2010027	5'-GAAGGT			
2009294	5'-TGGTTT		2010028	5'-CGTACT			
2009314	5'-TAATTT		2010052	5'-CAGTCT			

Table S4 – Estimation of Experimental Error

Confidence Level = 0.95			Confidence Level = 0.99		
Range of linear Error	± Log_2 Error	Counts	Range of linear Error	± Log_2 Error	Counts
0.94 - 1.06	0.09	24301	0.94 - 1.06	0.09	-
0.93 - 1.07	0.1	13455	0.93 - 1.07	0.1	-
0.93 - 1.08	0.11	5907	0.93 - 1.08	0.11	-
0.92 - 1.09	0.12	4252	0.92 - 1.09	0.12	-
0.91 - 1.09	0.13	2883	0.91 - 1.09	0.13	-
0.91 - 1.10	0.14	2135	0.90 - 1.11	0.14	-
0.90 - 1.11	0.15	1471	0.90 - 1.11	0.15	14508
0.90 - 1.12	0.16	1074	0.90 - 1.12	0.16	4344
0.89 - 1.13	0.17	759	0.89 - 1.13	0.17	4252
0.88 - 1.13	0.18	505	0.88 - 1.13	0.18	4021
0.88 - 1.14	0.19	345	0.88 - 1.14	0.19	2480
0.87 - 1.15	0.2	280	0.87 - 1.15	0.2	2135

Table S5 – Data for AEI ratio measurements (Assay-2)

	Gene symbol	Het[1]	Source of RNA	Average reads[2] (gDNA)	Average AEI (gDNA)	CF[3] (gDNA)	SD[4] Log_2 AEI (gDNA)	Average reads[2] (cDNA)	smallest log_2 AEI (cDNA)	largest log_2 AEI (cDNA)	Prop[5] Log_2 AEI < 0 (cDNA)	Prop Log_2 AEI ~ 0 (cDNA)	Prop Log_2 AEI > 0 (cDNA)	Prop Log_2 AEI < 0 or > 0
1	*ADAM17*	7	PFC	31334	0.83	1.20	0.07	22108	-0.66	0.21	0.29	0.71	0	0.29
2	*AGER*	15	PFC	4609	0.94	1.06	0.14	1277	-0.07	1.23	0	0.47	0.53	0.53
3	*APBA2*	22	PFC	2932	0.87	1.15	0.12	8695	-0.4	1.88	0.36	0.55	0.09	0.45
4	*APH1A*[6]	22	PFC	-	-	-	-	-	-	-	-	-	-	-
5	*ARRB2*	11	PFC	21295	0.92	1.09	0.04	36188	-1.23	0.27	0.09	0.91	0	0.09
6	*ARVCF*	16	PFC	3642	0.97	1.03	0.17	5672	-0.65	0.44	0.18	0.57	0.25	0.43
7	*BACE1*	3	PFC	14904	0.92	1.09	0.07	75440	-0.61	-0.40	1	0	0	1

8	*BACE2*	16	PFC	18013	0.93	1.08	0.10	19897	-1.82	0.44	0.13	0.81	0.06	0.19
9	*BDNF*	15	PFC	15758	0.99	1.01	0.09	14943	-0.28	0.37	0	0.93	0.07	0.07
10	*CALHM1*	25	PFC	10061	1.12	0.89	0.08	1963	-1.52	0.73	0.28	0.6	0.12	0.4
11	*CH25H*	17	PFC	33250	1.17	0.85	0.13	12161	-0.06	1.53	0	0.12	0.88	0.88
12	*CLU*	20	PFC	12265	0.8	1.25	0.11	1782	-0.47	0.53	0.1	0.65	0.25	0.35
13	*CNR1*	24	PFC	6298	1.13	0.88	0.08	7334	-0.41	0.31	0.04	0.92	0.04	0.08
14	*CNTNAP2*	23	PFC	2717	1.03	0.97	0.13	5120	-0.71	0.45	0.13	0.66	0.21	0.34
15	*COMT*	21	PFC	1486	0.9	1.11	0.14	3292	-0.55	1.76	0.05	0.81	0.14	0.19
16	*CYFIP1*	21	PFC	6497	0.98	1.02	0.16	6320	-2.65	-0.22	1	0	0	1
17	*CYP2D6*	29	HIP	14631	1.63	0.61	0.46	34066	-1.6	0.71	0.69	0.24	0.07	0.76
18	*DBH*	36	PFC	10524	1.01	0.99	0.17	21864	-0.87	0.45	0.33	0.61	0.06	0.39
19	*DGCR14*	19	PFC	1132	0.99	1.01	0.12	1663	-0.15	0.1	0	1	0	0
20	*DGCR8*	19	PFC	3588	0.83	1.20	0.15	7990	-0.76	0.21	0.16	0.79	0.05	0.21
21	*DISC1*	29	PFC	11318	0.97	1.03	0.08	8304	-0.43	0.83	0.21	0.58	0.21	0.42
22	*DLG4*	25	PFC	12033	1.01	0.99	0.07	11016	0	0.46	0	0.72	0.28	0.28
23	*DRD1*	14	STR	1798	1.23	0.81	0.37	1664	-0.53	0.22	0.29	0.71	0	0.29
24	*DRD2*	21	STR	3773	0.93	1.08	0.13	16225	-1.51	0.15	0.43	0.57	0	0.43
25	*DRD3*	7	STR	10933	1.09	0.91	0.05	6183	-0.59	-0.05	0.57	0.43	0	0.57
26	*ERBB4*	20	PFC	8200	1.13	0.88	0.06	7445	-0.19	0.17	0	1	0	0
27	*FosB*	26	HIP	3703	1.13	0.88	0.09	10280	-0.25	0.40	0	0.88	0.12	0.12
28	*GAB2*	23	PFC	6905	0.68	1.47	0.11	11596	0.26	0.73	0	0.04	0.96	0.96
29	*GABRB3*	17	PFC	3481	1.03	0.97	0.10	6140	-0.29	0.40	0.06	0.88	0.06	0.12
30	*GABRG3*	20	PFC	4732	1.04	0.96	0.12	18130	-1.13	0.69	0.25	0.65	0.1	0.35
31	*GAL*	14	PFC	13054	0.83	1.21	0.07	19708	-0.41	0.92	0.14	0.43	0.43	0.57
32	*GNB1L*	26	PFC	2593	0.92	1.09	0.22	30222	-0.23	1.18	0.08	0.15	0.77	0.85
33	*GRIA2*	17	PFC	8366	0.81	1.24	0.12	8625	-0.57	-0.09	0.82	0.18	0	0.82
34	*GRIN2A*	21	PFC	4866	1.02	0.98	0.08	4128	-0.60	0.04	0.24	0.76	0	0.24

35	*GRIN2B*	14	PFC	25419	0.83	1.20	0.07	5860	0.03	0.50	0	0.93	0.07	0.07
36	*GRM3*	7	PFC	4765	0.99	1.01	0.30	25544	0.11	0.34	0	0.71	0.29	0.29
37	*GRN*	21	PFC	6688	0.94	1.06	0.07	24891	-0.06	0.27	0	1	0	0
38	*GSK3B*	20	PFC	7442	0.96	1.04	0.18	4018	-0.59	0.06	0.60	0.40	0	0.60
39	*HTR1B*	27	PFC	1369	1.03	0.97	0.10	1527	-0.05	0.69	0	0.70	0.30	0.30
40	*HTR2A*	22	PFC	5512	1.06	0.94	0.08	3552	-1.91	0.43	0.09	0.73	0.18	0.27
41	*HTR3B*	18	AMG	16021	1.14	0.87	0.07	16263	-0.38	0.40	0.06	0.83	0.11	0.17
42	*LRP1*	22	PFC	23513	1.06	0.94	0.09	28000	-1.42	0.15	0.05	0.95	0	0.05
43	*LRRK2*	27	PFC	10855	1.02	0.98	0.11	2377	-1.01	0.43	0.45	0.50	0.05	0.50
44	*MAPT*	8	PFC	15958	1.03	0.97	0.10	26229	-0.33	-0.02	0.13	0.87	0	0.13
45	*MCHR1*	4	PFC	4165	1.19	0.84	0.15	2912	-0.1	0.05	0	1	0	0
46	*MYT1L*	19	PFC	13023	1.25	0.8	0.23	17137	-1.98	0.17	0.74	0.21	0.05	0.79
47	*NDE1*	20	PFC	5405	0.99	1.01	0.09	5030	-0.38	1.18	0.20	0.70	0.10	0.30
48	*NGFR*	13	PFC	3911	0.75	1.33	0.09	3025	0.01	2.79	0	0.77	0.23	0.23
49	*NPY*	14	PFC	2003	1.03	0.97	0.13	51117	-0.63	0.04	0.07	0.93	0	0.07
50	*NRXN1*	26	PFC	7883	1.04	0.96	0.11	9573	0.19	0.59	0	0.04	0.96	0.96
51	*NTAN1*[6]	21	PFC	1649	1.22	0.82	0.27	-	-	-	-	-	-	-
52	*NTRK2*	25	PFC	2905	1.14	0.88	0.16	5415	0.18	0.63	0	0.04	0.96	0.96
53	*OPRD1*	19	HIP	22452	1.22	0.82	0.14	22719	-0.5	0.99	0.21	0.63	0.16	0.37
54	*OPRK1*	11	STR	7850	1.06	0.94	0.04	8927	-0.32	0.29	0.09	0.82	0.09	0.18
55	*OPRM1*	16	PFC	14428	1.22	0.82	0.05	54731	-0.88	0.26	0.56	0.44	0	0.56
56	*P25*	23	PFC	15598	1.57	0.64	0.14	51270	-0.42	0.17	0.17	0.83	0	0.17
57	*PDYN*	7	HIP	4692	0.9	1.11	0.09	10616	-0.18	0.43	0	0.86	0.14	0.14
58	*PICALM*	25	PFC	12246	0.65	1.54	0.11	16439	0.6	2.28	0	0	1	1
59	*PRODH*	17	PFC	3513	0.95	1.05	0.08	4882	-0.2	0.19	0	1	0	0
60	*PRRG4*	21	PFC	2444	1.09	0.91	0.13	16020	-0.19	0.18	0	1	0	0
61	*PSEN1*	10	PFC	11947	0.97	1.03	0.10	34746	-0.05	0.12	0	1	0	0

62	*PSEN2*	25	PFC	16678	0.94	1.06	0.05	26780	-0.43	1.29	0.08	0.84	0.08	0.16
63	*RAI1*	15	PFC	5338	1.03	0.97	0.07	8625	-1.3	0.10	0.60	0.40	0	0.60
64	*RGS4*	21	PFC	14024	1	1	0.13	18755	-0.38	1.45	0.14	0.67	0.19	0.33
65	*SLC1A2*	19	PFC	4188	0.99	1.01	0.07	9840	-0.13	0.29	0	0.95	0.05	0.05
66	*SLC6A2*[6]	25	PFC	9721	1.07	0.93	0.04	-	-	-	-	-	-	-
67	*SLC6A3*	7	SBN	13720	0.96	1.04	0.28	24692	-0.11	0.31	0	0.86	0.14	0.14
68	*SORCS1*	20	PFC	30191	0.92	1.09	0.05	3857	-0.53	1.78	0.15	0.65	0.20	0.35
69	*SORL1*	20	PFC	20348	0.91	1.10	0.06	24841	-0.55	0.13	0.15	0.85	0	0.15
70	*TBX1*	29	PFC	15462	1.01	0.99	0.11	8095	-0.97	1.12	0.31	0.45	0.24	0.55
71	*TNFRSF1*	13	PFC	11795	2	0.50	0.09	2746	-1.54	0.08	0.92	0.08	0	0.92
72	*TNFRSF21*	22	PFC	8647	0.89	1.12	0.14	18772	-0.53	0.36	0.09	0.86	0.05	0.14
73	*TNK1*	19	PFC	1079	1.03	0.97	0.15	4838	-1.69	0.52	0.27	0.68	0.05	0.32
74	*ZNF804A*	8	PFC	8012	0.99	1.01	0.06	7130	-0.1	0.09	0	1	0	0

(1) Number of brain samples heterozygous for mSNP, (2) average sequencing reads for the indicated gene in a single sample = sum of the reads for each of the two mSNP alleles in a single sample; (3) gDNA AEI correction factor, (4) Standard deviation of log_2 (normalized gDNA AEI ratios); (5) proportion; (6) No gDNA and/or cDNA AEI results due to insufficient number of sequencing reads.

Table S6- SNPs within PCR primer binding sites

Gene	SNP	Heterozygosity*	gDNA log_2AEI
HTR2A	rs1805055	0	-0.01
TBX1	rs41298816	0	0.03
BDNF	rs1048218	0.008	0.09
AGER	rs35795092	0.162	-0.08
NR2B	rs1805504	unknown	0.03
GRN	rs1059738	“	-0.09
HTR3B	rs34550504	“	0.19
PICALM	rs1043858<br>rs318352	“<br>“	-0.61
TNFRSF1	rs4441073	“	1.00

(*) Han Chinese population (SNP database, NCBI)
