# Supplementary material for: Next-generation DNA sequencing-based assay for measuring allelic expression imbalance (AEI) of candidate neuropsychiatric disorder genes in human brain
Source: BMC Genomics. 2011 Oct 20;12:518. doi: 10.1186/1471-2164-12-518 (PMC3228908; doi:10.1186/1471-2164-12-518)
Supplement: Additional file 3 — Correction factors for AEI ratios and criteria for the presence or absence of AEI in individual samples. A discussion of factors that influence the measurement of genomic DNA AEI ratios and criteria for assessing whether individual samples show allele-specific differences in mRNA expression. [file 1471-2164-12-518-S3.PDF]

Additional File 3 – Factors that influence genomic DNA AEI ratios and criteria for the absence or presence AEI in individual samples

A. Factors that influence genomic DNA AEI ratios

In addition to PCR amplification bias, the presence of pseudogenes, highly homologous genes and/or chromosomal duplications can distort genomic (g) DNA AEI ratios. As mentioned in the main text, analysis of the DNA sequences of the primer sets and PCR products for the 74 candidate genes in this study using NCBI's Primer-BLAST and/or UCSC's *In-silico* PRC, predicted unique target sites for all but two (*CYP2D6* and *NTAN1*) , suggesting that off-target PCR amplification of known genomic sequences was not a significant cause for deviation of genomic DNA AEI ratios from 1.0.

The possible influence of copy number variants (CNVs) on gDNA AEI ratios is a little more difficult to assess. Deletions that include the marker SNP, would have no effect, since samples missing one allele would be scored as "homozygotes" and not assayed for gDNA or cDNA AEI. By contrast, duplications that include the marker SNP would double the number of sequencing reads of one of the two alleles. Depending upon which allele is affected, duplications would produce AEI ratios approximately equal to twice or one-half the true heterozygote AEI ratio. Because most CNV-related duplications are rare, however, one would not expect to observe them in small sets of samples. Furthermore, the presence of one or two-samples that deviate two-fold from the mean would usually have only a small effect on the average genomic AEI and therefore only a small effect on the correction factor. Nevertheless, the possible presence of duplications

should be examined and the potential impact on the gDNA AEI ratios and correction factor assessed.

Reviewing our current data for each gene showed no clear examples of individual samples that deviated 2-fold from the mean AEI ratio, suggesting that it is unlikely that chromosomal duplications (CNVs) contribute to deviations of gDNA AEI ratios from 1.0. In absence of contrary evidence, we conclude that, except for *CYP2D6* and *NTAT1*, deviations of gDNA AEI ratios from 1.0 result from differential amplification of the respective alleles.

B. Criteria for the absence or presence AEI in individual samples

Previous studies have accessed the presence or absence of AEI in individual samples by comparing the normalized (i.e, corrected) gDNA AEI ratio and the cDNA ratio derived for each sample. For example, Lim *et al.* [1], required that there be no overlap between 2 x SEM for each of these ratios for the sample to show significant AEI. A review of our current results, however, suggests that this approach may not be optimal, since outlying values of normalized gDNA AEI ratios in individual samples tend to disqualify otherwise perfectly acceptable cDNA AEI ratios. We now think that, with rare exceptions, small deviations of normalized gDNA AEI ratios from 1.0 are most likely due to experimental error, rather than true differences in the genomic copy number. For this reason, we decided to evaluate the presence or absence of AEI in individual samples based on estimates of experimental error determined from the entire set of normalized gDNA AEI ratios. As described in Results, we used two methods to estimate this experimental error.

In the first method, we examined the distribution of 1,371 $\log_2$ normalized gDNA AEI ratios in our data set. The mean for this distribution was 0.0 (by definition), with a standard deviation = 0.1415 and standard error of the mean = 0.0038. Although the distribution of $\log_2$ normalized gDNA AEI ratios was symmetrical with respect to the mean, the AEI ratios were not normally distributed (Anderson-Darling Normality Test $A^2 = 33.67$; $p = 0.005$). For this reason, we estimated probabilities for various magnitudes of experimental error directly from the list of $\log_2$ normalized AEI ratios. This analysis showed that 95% of the ratios fell within the interval ± 0.29 (corresponding to a range for linear corrected AEI ratios of 0.82 to 1.22). Based on these results, we decided to use ± 0.29 as the criterium for AEI: $\log_2$ normalized cDNA AEI ratios less than -0.29 or greater than +0.29 were classified as AEI-positive, while corrected cDNA ratios between -0.29 and +0.29 were classified as AEI-negative. On a linear scale: normalized AEI ratios between 0.82 and 1.22 were classified as AEI-negative, while $\log_2$ normalized AEI ratios outside of this range were classified as AEI-positive. The proportion of samples showing or not showing AEI are listed in Additional file 1, Table 5S and graphically displayed in Fig 3.

In the second method, which we used primarily for mathematical modeling, estimates of experimental error were based on the total number of sequencing reads used to calculate the cDNA $\log_2$AEI ratio for each gene in each brain sample (i.e., reads in numerator + reads in denominator). As described in Results, correlations between experimental error and sequencing read number for cDNA ratios were estimated from the data in Additional

file 1, Fig. 6S(b), based upon the assumption that experimental error is the same for cDNA- and gDNA-based measurements. Single measurements of gDNA-based or cDNA-based $\log_2$AEI ratios (i.e., the $\log_2$AEI ratios the we obtain for a specific gene in a specific sample), can be considered as “point estimates” for the “true” AEI ratios in that sample, with the error terms defining the “confidence interval” for that ratio at a defined level of confidence (e.g., 95% or 99%). To assess the presence of absence of AEI in a given sample, we evaluated $\log_2$ corrected cDNA AEI ratios based simply upon their associated experimental error, as determined by the total number of sequence reads used to calculate the ratio. Following this line of reasoning, we defined samples as having AEI if the measured $\log_2$AEI value was different from 0 and the interval of associated experimental error did not include 0.

The first method described above provides a relatively strict criterium for the presence of AEI, while the second is more liberal: 8 genes were judged to have no samples showing AEI using the first method, while only 2 genes had no samples showing AEI using the second method. Distinguishing genuine, but small AEI ratios from experimental error, however, is extremely difficult, and it is probable that no single criterium will allow every sample to be classified correctly. Rather, combining statistical analysis of the data with mathematical modeling is likely to provide a more accurate estimate of the actual numbers of samples with significant AEI.

Reference:

1. Lim, J.E., et al., *Allelic expression of serotonin transporter (SERT) mRNA in human pons: lack of correlation with the polymorphism SERTLPR.* Mol Psychiatry, 2006. **11**(7): p. 649-62.
