# Supplementary material for: Next-generation DNA sequencing-based assay for measuring allelic expression imbalance (AEI) of candidate neuropsychiatric disorder genes in human brain
Source: BMC Genomics. 2011 Oct 20;12:518. doi: 10.1186/1471-2164-12-518 (PMC3228908; doi:10.1186/1471-2164-12-518)
Supplement: Additional file 4 — Modeling population distributions of log2AEI ratios. A brief outline of our method for modeling AEI ratios, including a description of the modeling of log2AEI population distributions for GAB2, GNB1L and DISC1. [file 1471-2164-12-518-S4.PDF]

Additional File 4 - Modeling population distributions of log_2AEI ratios

(A detailed description of the methods used in this section will be published separately: Sun Y, *et al*., manuscript in preparation.)

A. Preliminaries

Procedure for modeling log_2AEI distributions:

1) Based upon the shape of the experimentally determined log_2AEI distribution and known major allele frequency of the marker (m)SNP [P(M)], make rough estimates of the number of rVar's and their major allele frequencies [P(A), P(B), etc], linkage to the marker (m)SNP and each other [D'(AM), D'(BM), D'(AB), etc.] and individual contributions to log_2AEI (j, k, l, etc.);

2) Calculate the expected population frequencies for haplotypes containing the mSNP and the estimated number of rVar's;

3) Calculate the expected population frequencies of diplotypes that arise from combinations of these haplotypes;

4) Create a log_2AEI contribution matrix listing the estimated log_2AEI produced by each diplotype;

5) Multiply the expected diplotypes frequencies by the number of samples used to generate the experimentally determined log_2AEI distributions, rounding off numbers where appropriate;

6) Graph predicted log_2AEI distribution and compare with the experimentally determined log_2AEI distribution; The goodness of fit of the model can be assessed by: i) calculating the fraction (percentage) of predicted log_2AEI ratios that lie within the bounds of the experimentally determined log_2AEI ratios ± experimental error and/or ii) by linear regression analysis of the experimentally determined log_2AEI ratios vs ratios predicted by the model;

7) Adjust input values of parameters as needed to obtain the closest possible match.

Examples:

i) Model #1: one *cis*-acting regulatory variant (rVarA; alleles: A/a) with varying degrees of linkage to the marker SNP (mSNP; alleles: M/m):

Haplotypes: AM, aM, Am, am

Expected haplotype frequencies:

P(AM) = P(A)P(M) + D(AM)
P(aM) = P(a)P(M) – D(AM)
P(Am) = P(A)P(m) – D(AM)
P(am) = P(a)P(m) + D(AM)

Where D(AM) = P(AM)- P(A)P(M)

Note: to ensure that all haplotype frequencies are non-negative, the second-order LD constant D(AM) is subject to the following constraints:

$[-P(A)P(M), -P(a)P(m)] \leq D(AM) \leq [P(A)P(m), P(a)P(M)]$

Following RC Lewontin [1], the normalized second-order LD constant D'(AM) is defined as;

i) $D'(AM) = D(AM)/D(AM)_{max}$, where $D(AM)_{max}$ = the <u>smaller</u> of P(M)P(a) and P(m)P(A), when $D(AM) > 0$;
ii) $D'(AM) = D(AM)/|D(AM)_{min}|$, where $|D(AM)_{min}|$ = the <u>smaller</u> of P(M)P(A) and P(m)P(a), when $D(AM) < 0$.

Note: Under these definitions, D'(AM) takes on values between -1 and +1.

Expected diplotypes:

	AM	aM	Am	am
**am**	**AM/am**	**aM/am**	**Am/am**	**am/am**
**Am**	**AM/Am**	**aM/Am**	**Am/Am**	**am/Am**
**aM**	**AM/aM**	**aM/aM**	**Am/aM**	**am/aM**
**AM**	**AM/AM**	**aM/AM**	**Am/AM**	**am/AM**

Expected diplotype frequencies:

	P(AM)	P(aM)	P(Am)	P(am)
**P(am)**	**P(AM) P(am)**	**P(aM) P(am)**	**P(Am) P(am)**	**P(am) P(am)**
**P(Am)**	**P(AM) P(Am)**	**P(aM) P(Am)**	**P(Am) P(Am)**	**P(am) P(Am)**
**P(aM)**	**P(AM) P(aM)**	**P(aM) P(aM)**	**P(Am) P(aM)**	**P(am) P(aM)**
**P(AM)**	**P(AM) P(AM)**	**P(aM) P(AM)**	**P(Am) P(AM)**	**P(am) P(AM)**

Expected contributions to $\log_2$AEI from rVarA .

	AM	aM	Am	am
**am**	**j**	**0**	**[-]**	**[-]**
**Am**	**0**	**-j**	**[-]**	**[-]**
**aM**	**[-]**	**[-]**	**-j**	**0**
**AM**	**[-]**	**[-]**	**0**	**j**

Note: AEI ratios are measured only for samples heterozygous for the marker SNP.

Effect of varying D’(AM) on log_2AEI distributions for Model#1 (j = 0.8):

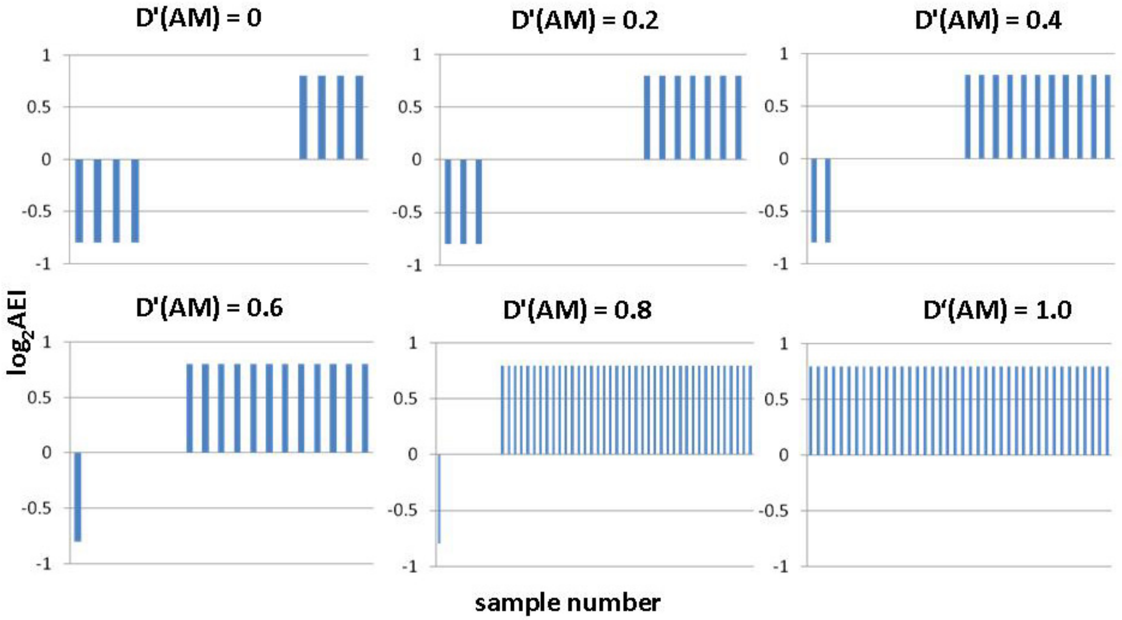

Note: Linkage between one or more rVar’s and mSNP produces asymmetry (skewing) of the log_2AEI distribution.

ii) Model #2: two *cis*-acting rVar's (rVarA and rVarB), with rVarA and rVarB have varying degrees of linkage to each other and the mSNP.

Haplotypes: ABM, aBM, AbM, abM, ABm, aBm, Abm and abm;

Expected haplotype frequencies [2], where D(ABM) = third-order LD constant:

$$
\begin{aligned}
P(ABM) &= P(A)P(B)P(M) + P(A)D(BM) + P(B)D(AM) + P(M)D(AB) + D(ABM)\\
P(aBM) &= P(a)P(B)P(M) + P(a)D(BM) - P(B)D(AM) - P(M)D(AB) - D(ABM)\\
P(AbM) &= P(A)P(b)P(M) - P(A)D(BM) + P(b)D(AM) - P(M)D(AB) - D(ABM)\\
P(abM) &= P(a)P(b)P(M) - P(a)D(BM) - P(b)D(AM) + P(M)D(AB) + D(ABM)
\end{aligned}
$$

$$
\begin{aligned}
P(ABm) &= P(A)P(B)P(m) - P(A)D(BM) - P(B)D(AM) + P(m)D(AB) - D(ABM)\\
P(aBm) &= P(a)P(B)P(m) - P(a)D(BM) + P(B)D(AM) - P(m)D(AB) + D(ABM)\\
P(Abm) &= P(A)P(b)P(m) + P(A)D(BM) - P(b)D(AM) - P(m)D(AB) + D(ABM)\\
P(abm) &= P(a)P(b)P(m) + P(a)D(BM) + P(b)D(AM) + P(m)D(AB) - D(ABM)
\end{aligned}
$$

Note: the values of second and third-order LD constants are subject to additional constraints related to the requirement that population frequencies for all haplotypes remain non-negative [3].

Expected diplotype frequencies:

	P(ABM)	P(aBM)	P(AbM)	P(abM)	P(ABm)	P(aBm)	P(Abm)	P(abm)
P(abm)	P(ABM)P(abm)	P(aBM)P(abm)	P(AbM)P(abm)	P(abM) P(abm)	P(ABm)P(abm)	P(aBm)P(abm)	P(Abm)P(abm)	P(abm)P(abm)
P(Abm)	P(ABM)P(Abm)	P(aBM)P(Abm)	P(AbM)P(Abm)	P(abM)P(Abm)	P(ABm)P(Abm)	P(aBm)P(Abm)	P(Abm)P(Abm)	P(abm)P(Abm)
P(aBm)	P(ABM)P(aBm)	P(aBM)P(aBm)	P(AbM)P(aBm)	P(abM) P(aBm)	P(ABm)P(aBm)	P(aBm)P(aBm)	P(Abm)P(aBm)	P(abm)P(aBm)
P(ABm)	P(ABM)P(ABm)	P(aBM)P(ABm)	P(AbM)P(ABm)	P(abM)P(ABm)	P(ABm)P(ABm)	P(aBm)P(ABm)	P(Abm)P(ABm)	P(abm)P(ABm)
P(abM)	P(ABM)P(abM)	P(aBM)P(abM)	P(AbM)P(abM)	P(abM)P(abM)	P(ABm)P(abM)	P(aBm)P(abM)	P(Abm)P(abM)	P(abm)P(abM)
P(AbM)	P(ABM)P(AbM)	P(aBM)P(AbM)	P(AbM)P(AbM)	P(abM)P(AbM)	P(ABm)P(AbM)	P(aBm)P(AbM)	P(Abm)P(AbM)	P(abm)P(AbM)
P(aBM)	P(ABM)P(aBM)	P(aBM)P(aBM)	P(AbM)P(aBM)	P(abM)P(aBM)	P(ABm)P(aBM)	P(aBm)P(aBM)	P(Abm)P(aBM)	P(abm)P(aBM)
P(ABM)	P(ABM)P(ABM)	P(aBM)P(ABM)	P(AbM)P(ABM)	P(abM)P(ABM)	P(ABm)P(ABM)	P(aBm)P(ABM)	P(Abm)P(ABM)	P(abm)P(ABM)

Expected contributions to log_2AEI from rVarA and rVarB for various diplotypes (For simplicity, this model assumes that the effects of alleles of independent variants on mRNA expression are multiplicative on a linear scale and additive on a log_2 scale):

	P(ABM)	P(aBM)	P(AbM)	P(abM)	P(ABm)	P(aBm)	P(Abm)	P(abm)
P(abm)	j+k	k	j	0	[-]	[-]	[-]	[-]
P(Abm)	k	-j+k	0	-j	[-]	[-]	[-]	[-]
P(aBm)	j	0	j-k	-k	[-]	[-]	[-]	[-]
P(ABm)	0	-j	-k	-j-k	[-]	[-]	[-]	[-]
P(abM)	[-]	[-]	[-]	[-]	-j-k	-k	-j	0
P(AbM)	[-]	[-]	[-]	[-]	-k	j-k	0	j
P(aBM)	[-]	[-]	[-]	[-]	-j	0	-j+k	k
P(ABM)	[-]	[-]	[-]	[-]	0	j	k	j+k

Note: AEI ratios are measured only for samples heterozygous for the marker SNP.

The effect of increasing D'(AM) on log_2AEI distributions for Model #2 (j = 0.8; k = 0.6):

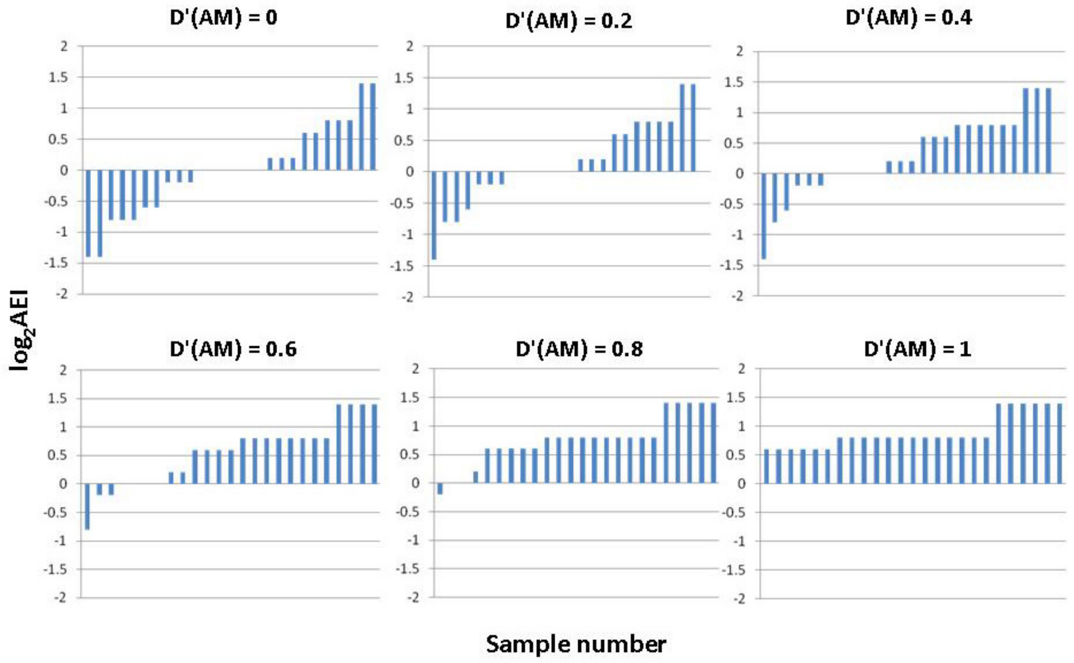

Note: Two *cis*-acting regulatory variants can combine to produce a gradient of log_2AEI values. Again, linkage between one or more of the rVar's and mSNP produces skewing of the distribution.

iii) Models containing one *cis*-acting variant (rVarA) plus a single *trans*-acting variant (rVarT) or complex non-*cis*-acting factors that modify the AEI ratios generated by rVarA.

Trans-acting variants can modify AEI produced by a *cis*-acting variant, but cannot by themselves produce AEI. For example, the effectiveness of a transcription factor to stimulate mRNA expression may be influenced by a SNP within its binding site in the promoter of its target gene. Such a SNP would be considered a *cis*-acting regulatory variant for the target gene. If the SNP produces a significant difference in the ability of the transcription factor to bind to the promoter, AEI of mRNA expression may result. On the other hand, a missense variant in the coding region of the transcription factor gene might produces a transcription factor with increased or decreased binding affinity for one of the two DNA sequences generated by the *cis*-acting SNP. Such a missense variant would be considered to be a *trans*-acting regulatory variant with respect to it target gene. If the effect is sufficiently large, the population distribution of $\log_2$AEI ratios would change. The ability of the *trans*-acting variant to modify AEI ratios, however, depends entirely upon its interaction with the *cis*-acting variant.

Similar arguments hold for the effects of complex *trans*-genetic, epigenetic or non-genetic factors on $\log_2$AEI population distributions: any effect that changes AEI ratios depends upon interactions with one or more *cis*-acting variants. Thus, allele-specific changes in gene expression related to DNA methylation, for example, depend upon allele-specific differences in methylation. An important prediction that follows from this requirement is that non-*cis*-acting factors produce changes in $\log_2$AEI distributions in a manner that leaves the fraction of samples showing no-AEI unchanged. By contrast, additional *cis*-acting variants usually reduce the fraction of samples showing no-AEI. These following examples show how this principle can be incorporated into models for non-*cis*-acting factors.

1) Modeling the contribution of a single *cis*-acting regulatory variant, rVarA, plus a single *trans*-acting regulatory variant, rVarT, is carried out exactly as described for two *cis*-acting regulatory variants, with the exception of the values assigned for the contributions of various combinations to log_2AEI ratios.

Example: Assume contributions to log_2AEI from one *cis*-acting variant rVarA and a single *trans*-acting variant (rVarT) follow these rules: 1) the *trans*-acting regulatory variant, rVarT, contributes to AEI only in samples heterozygous for the *cis*-acting variant, rVarA; 2) the log_2 contribution of the two T alleles (i.e. genotype T/T) = 2m, the contribution of one T allele (i.e. genotype T/t) = m, the contribution of no T alleles (i.e., genotype t/t) = 0m = 0; 3) m can be assigned positive or negative values, but to ensure consistency of effect, the final sign of m is opposite for positive and negative values of j: i.e., m is: i) <u>positive</u> when j is positive and <u>negative</u> when j is negative or ii) <u>negative</u> when j is positive and <u>positive</u> when j is negative. These rules are implemented in the log_2AEI-contribution matrix below.

Expected contributions to log_2AEI from rVarA and rVarT for various diplotypes: (For simplicity, this model assumes that the effects of alleles of independent variants on mRNA expression are multiplicative on a linear scale and additive on a log_2 scale.):

	**P(ATM)**	**P(aTM)**	**P(AtM)**	**P(atM)**	**P(ATm)**	**P(aTm)**	**P(Atm)**	**P(atm)**
**P(atm)**	**j + m**	**0**	**j + 0m**	**0**	**[-]**	**[-]**	**[-]**	**[-]**
**P(Atm)**	**0**	**-j - m**	**0**	**-j**	**[-]**	**[-]**	**[-]**	**[-]**
**P(aTm)**	**j + 2m**	**0**	**j + m**	**0**	**[-]**	**[-]**	**[-]**	**[-]**
**P(ATm)**	**0**	**-j - 2m**	**0**	**-j - m**	**[-]**	**[-]**	**[-]**	**[-]**
**P(atM)**	**[-]**	**[-]**	**[-]**	**[-]**	**-j - m**	**0**	**-j + 0m**	**0**
**P(AtM)**	**[-]**	**[-]**	**[-]**	**[-]**	**0**	**j + m**	**0**	**j**
**P(aTM)**	**[-]**	**[-]**	**[-]**	**[-]**	**-j - 2m**	**0**	**-j - m**	**0**
**P(ATM)**	**[-]**	**[-]**	**[-]**	**[-]**	**0**	**j + 2m**	**0**	**j + m**

Note: AEI ratios are measured only for samples heterozygous for the marker SNP.

2) Modeling the effects of complex trans-acting genetic, epigenetic and non-genetic factors on a single *cis*-acting

Example: Assume contributions to $\log_2$AEI from one *cis*-acting variant rVarA and a single trans-acting variant (rVarT) follow these rules: 1) the non-*cis*-acting regulatory variants contribute to AEI only in samples heterozygous for the *cis*-acting variant, rVarA; 2) the $\log_2$ contribution of these can be expressed in terms of percentage of the $\log_2$AEI contribution from rVarA.

Expected contributions to $\log_2$AEI from one *cis*-acting variant rVarA plus modulation of AEI rations due to complex non-*cis*-acting factors in individuals heterozygous for mSNP:

	**AM**	**aM**	**Am**	**am**
**am**	**j ± (j, 0.75j, 0.5j, 0.25j, 0j)**	**0**	**[-]**	**[-]**
**Am**	**0**	**-j ± (j, 0.75j, 0.5j, 0.25j, 0)**	**[-]**	**[-]**
**aM**	**[-]**	**[-]**	**-j ± (j, 0.75j, 0.5j, 0.25j, 0)**	**0**
**AM**	**[-]**	**[-]**	**0**	**j ± (j, 0.75j, 0.5j, 0.25j, 0)**

Note: For simplicity, we have introduced an arbitrary graded effect for the non-*cis*-acting factors on $\log_2$AEI.

Comparison of the predicted $\log_2$AEI population distributions for one *cis*-acting variant alone (a), or in combination with one *trans*-acting variant (b), complex non-*cis*-acting factors (c) or an additional *cis*-acting variant (d): (In these examples, P(A) = P(T) = P(B) = 0.5 and all variants are unlinked to each other and to the mSNP. The effect sizes are 0.8 for rVarA and 0.6 for rVarT and rVarB.)

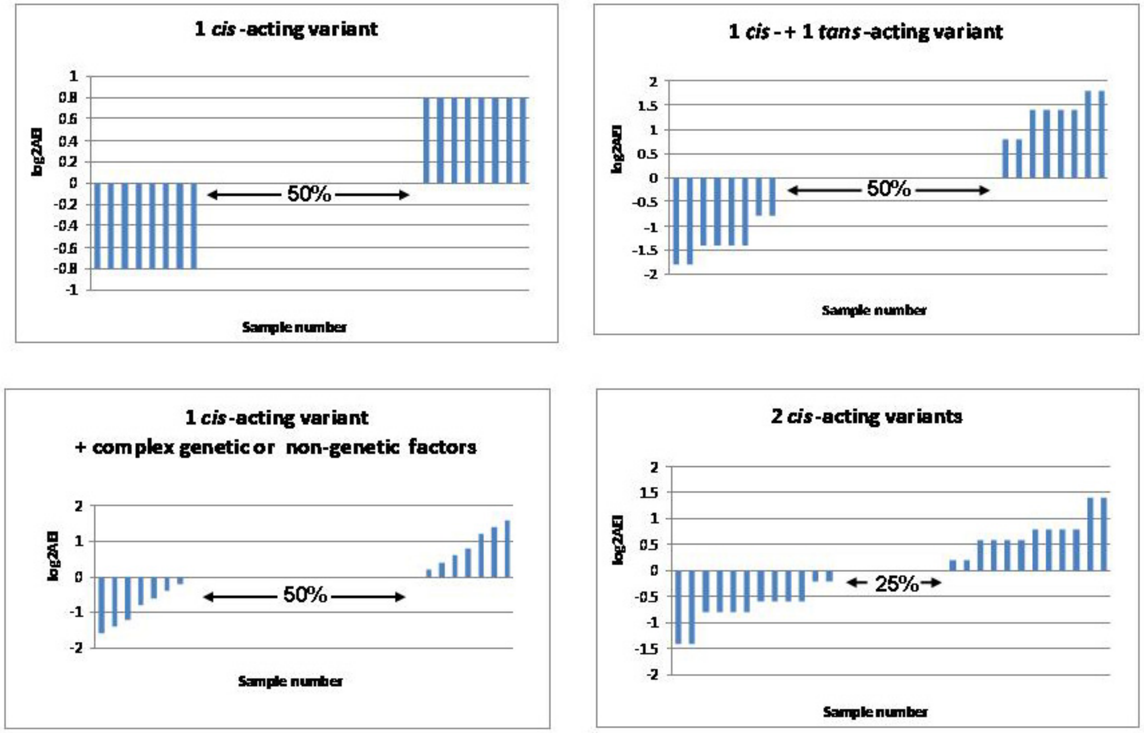

Note: Non-*cis*-acting factors modify AEI produced by a *cis*-acting variant, but do not change the percentage of samples showing no AEI. By contrast, additional *cis*-acting variants reduce the percentage of samples with no AEI.

Note on the purpose and limitations of modeling $\log_2$AEI distributions: We want to emphasize that the purpose of modeling $\log_2$AEI distributions is not to “prove” how genes are regulated, but rather to aid in the construction of testable hypotheses concerning the location, number and relative contributions of regulatory elements. For many genes, models differing significantly in detail will provide good matches with observed distributions. In such cases, further experimentation will be required to determine which model provides the best description of the mechanisms underlying differential gene regulation.

B. Modeling $\log_2$AEI distributions for *GAB2*

The observation that all of the $\log_2$AEI ratios are in the same direction (positive) and of roughly the same magnitude (0.45 $\log_2$AEI units) suggests that mRNA expression is controlled by a single rVar (rVarA) that is in complete linkage disequilibrium [D'(AM) =1] with the marker SNP (rs1046780). The shallow gradient in $\log_2$AEI values can be accounted for in several ways: 1) two additional regulatory variants that are unlinked to the mSNP, each contributing 0.1 $\log_2$AEI units; and 3) complex genetic or non-genetic factors. The diagram below is based on the first model, with input values of: P(A) = 0.6; P(B) = 0.4; P(C) = 0.6; P(M) = 0.6; D'(AM) = 1, all additional second-, third- and fourth-order LD constants = 0; j = 0.45, k = 0.1, l = 0.1. Log_2 experimental error bars were estimated from the number of sequencing reads (listed in Table S5) used to calculate the experimentally determined $\log_2$AEI ratios, as described in the legend to Fig S6. In all, 22 out of 23 of the predicted $\log_2$AEI ratios (96%) match the measured $\log_2$AEI values ± experimental error. Linear regression analysis confirmed a close correlation between the model and experimental data: coefficient of determination = 0.79.

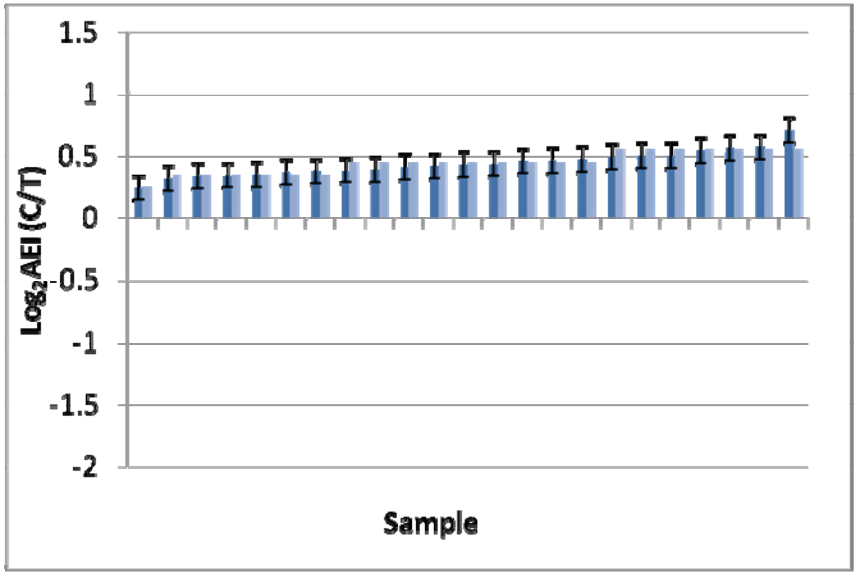

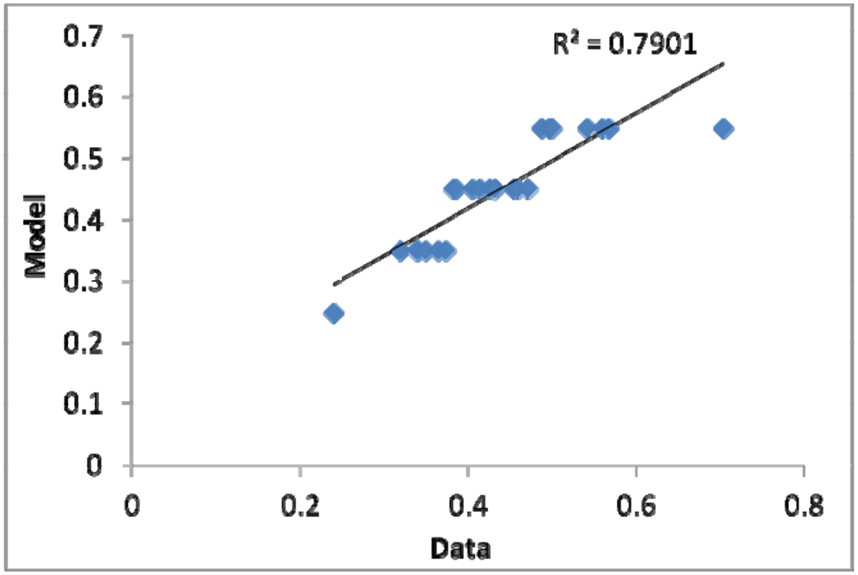

C. Modeling log_2AEI distributions for *GNB1L*

The skewed log_2AEI distribution obtained for *GNB1L* varies more that would be expected from experimental error alone, suggesting the involvement of at least two rVar's. In addition, the presence of several samples with negative or non-significant log_2AEI ratios suggests that one of the variants is partially linked to the mSNP. Based upon these inferences, we selected the following input values for the model : P(A) = 0.6, P(B) = 0.7, P(M) = 0.52, D'(AM) = 0.85, D'(BM) = D'(AB) = 0; j= 0.7, k=0.4. Log_2 experimental error terms were estimated from the number of sequencing reads used to calculate the experimentally determined log_2AEI ratios. In all, 20 out of 26 of the predicted log_2AEI ratios (77%) match the measured log_2AEI values ± experimental error (with several additional samples just missing the mark). Linear regression analysis confirmed a close correlation between the model and experimental data: coefficient of determination = 0.9465.

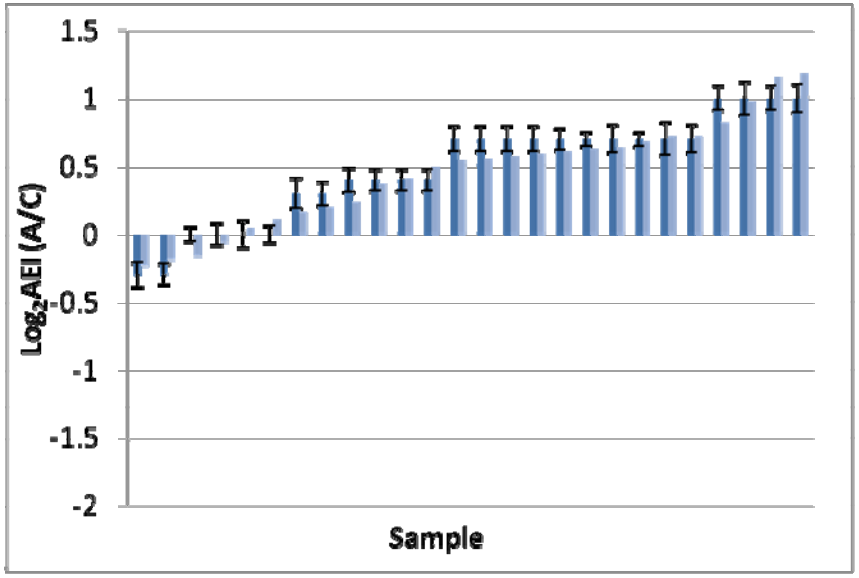

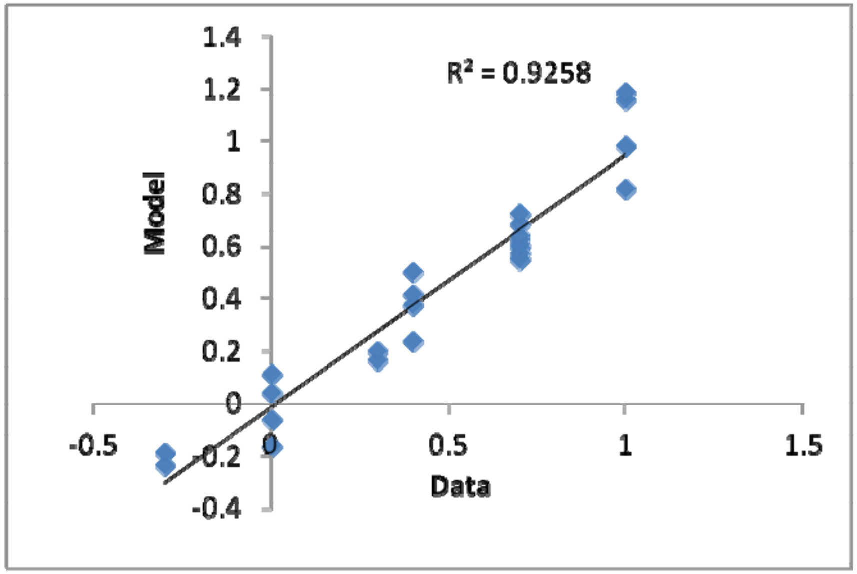

D. Modeling log_2AEI distributions for *DISC1*

The nearly equally balanced, biphasic log_2AEI distribution obtained for *DISC1*, suggests the influence of multiple regulatory variants, which are all unlinked to the mSNP. The presence of only two out of 29 samples with relatively large log_2AEI values, suggest the presence of a relatively rare regulatory variant of large effect plus one or two regulatory variants of small effect. Based on these inferences, we selected the following input values for the model: P(A) = 0.4, P(B) = 0.05, P(C) = 0.1; P(M) =0.4; all LD (D') constants = 0; j= 0.4, k= 1, l = 0.15. Again, the error terms were estimated from the number of sequencing reads used to calculate the experimentally determined log_2AEI ratios. In all, 26 out of 28 of the predicted log_2AEI ratios (93%) match the measured log_2AEI values ± experimental error. Linear regression analysis confirmed a close correlation between the model and experimental data: coefficient of determination = 0.952.

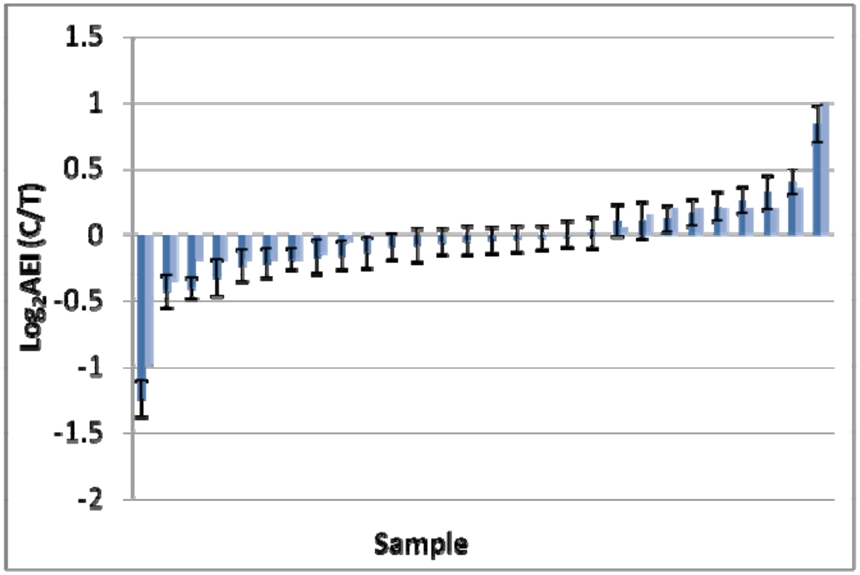

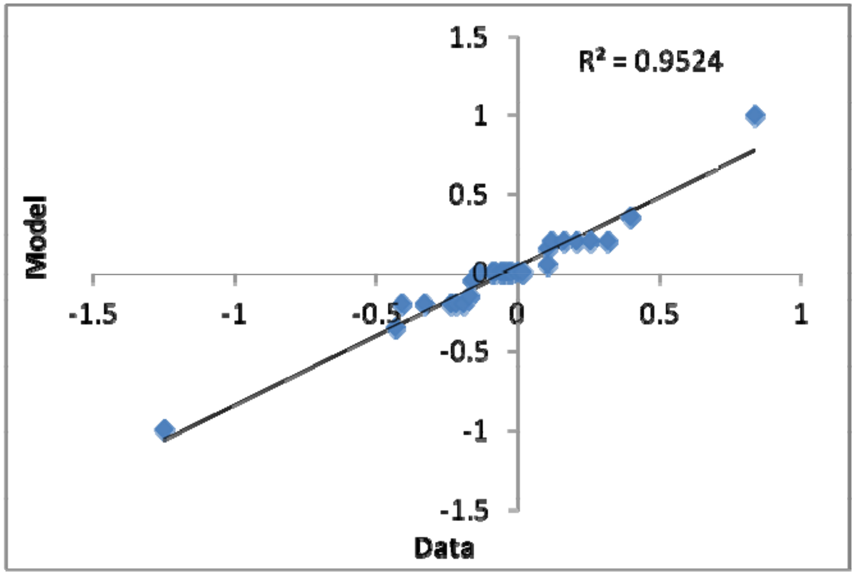

References

1. Lewontin RC: **The Interaction of Selection and Linkage. I. General Considerations; Heterotic Models**. *Genetics* 1964, **49**(1):49-67.
2. Weir BS: **Linkage disequilibrium and association mapping**. *Annual review of genomics and human genetics* 2008, **9**:129-142.
3. Robinson WP, Asmussen MA, Thomson G: **Three-locus systems impose additional constraints on pairwise disequilibria**. *Genetics* 1991, **129**(3):925-930.
