# Supplementary material for: Next-generation DNA sequencing-based assay for measuring allelic expression imbalance (AEI) of candidate neuropsychiatric disorder genes in human brain
Source: BMC Genomics. 2011 Oct 20;12:518. doi: 10.1186/1471-2164-12-518 (PMC3228908; doi:10.1186/1471-2164-12-518)
Supplement: Additional file 5 — GAB2, GNB1L and DISC1: AEI measurements and modeling. A discussion of inferences drawn from the modeling of GAB2, GNB1L and DISC1 in the context of previously published studies on the regulation of these genes. [file 1471-2164-12-518-S5.PDF]

Additional File 5 -

GAB2, *GNB1L* and *Disc1*: AEI measurements and modeling

1) *GAB2* (Grb2-associated binder 2; Chr11q14.1) encodes a 676 amino acid residue protein that functions as a molecular scaffold, linking Grb2 or Grb2/Shc complexes bound to membrane receptor tyrosine kinases and cytokine receptors to downstream effectors such as phospholipase Cγ, PI3 kinase, and the molecular adapters Shp2 and Crk [1]. GAB2 was first shown to modify genetic risk for Alzheimer's disease (AD) in *APOE4* carriers in a large genome-wide association study [2, 3] and remains one of the most highly replicated candidate AD genes [4], although there is significant heterogeneity among studies [5]. Association studies for GAB2 and AD in the Han Chinese population, have produced mixed results [6-8].

As described in the main manuscript, the results of our AEI assays and mathematical modeling, suggest that *GAB2* is regulated by a *cis*-acting genetic variant that is tightly linked to the mSNP used to distinguish *GAB2* alleles (rs1046780). Identifying this regulatory variant, however, is made difficult by the fact that *GAB2* is located entirely within a 189 kb haplotype block that includes more than 614 SNPs. Reiman et al. [3] identified a common haplotype comprising alleles of 10 *GAB2* SNPs that is associated with increased risk for late onset Alzheimer's disease (LOAD) in *APOE4* carriers. Because all of the known *GAB2* missense variants have very low minor allele frequencies in European and Chinese populations, the common variant that contributes to AD may do so by influencing *GAB2* mRNA expression.

Based on linkage data for HapMap SNPs in the European and Chinese populations and analysis of genotyping data from our 52 brain samples, we found that the high-expression mSNP allele (i.e., the *T*-allele) also belongs to the LOAD risk haplotype. Based on our AEI measurements, we predict that individuals homozygous for the mSNP *T*-allele express *GAB2* mRNA at levels approximately 40% higher than individuals homozygous for the *C*-allele.

Our observation that the LOAD risk haplotype is a high-expression haplotype for *GAB2* mRNA, is consistent with the observation of Reiman *et al.* [3] that regions of the brain prone to develop AD-related neurofibrillary tangles (in particular, the hippocampus and posterior cingulate cortex) express higher levels of *GAB2* mRNA compared to regions with less prone to develop tangles (e.g., the visual cortex). It is not necessarily consistent, however, with the results of an experiment in the same study, showing that phosphorylation of serine-262 of wild-type tau is increased following pretreatment of neuroglioma cells with siRNA directed against *GAB2* mRNA. (Because phosphorylated tau is a component of neurofibrillary tangles in AD brain, one might expect that low-expression of *GAB2* mRNA would correlate with increased risk.) The fact that GAB2 functions in multiple intracellular signaling pathways and the possibility that tau phosphorylation may be regulated differently in neuroglioma cells (which over-express tau) and neurons, however, suggest that additional work is required to determine how differential expression of *GAB2* mRNA contributes to LOAD risk and/or protection.

2) *GNB1L* (guanine nucleotide binding protein, beta-polypeptide 1-like; Chr22q11.2) encodes an 327 amino acid residue protein of unknown function that contains six WD40 repeats, similar to those in the G-protein beta-subunit [9, 10]. The gene is located within the ~1.5 M bp velocardiofacial (VCF)/DiGeorge (DG) critical region on chromosome 22. Hemizygous deletion of this critical region produces cleft palate, congenital heart disease and a variety of other developmental abnormalities affecting the circulatory, skeletal and endocrine systems, although usually not all of these defects are present in individual patients [11]. Deletion of the VCR/DG critical region is also associated with high rates of learning disabilities and psychiatric disorders in children, including autism, attention deficit/hyperactivity disorder, anxiety disorders and depression [12, 13], and obsessive compulsive disorder [14] and schizophrenia in adults [15, 16].

Genetic studies in European [17] and Han Chinese populations [18] have found associations between SNPs in the 3'-end of the *GNB1L* and schizophrenia. The Chinese study also demonstrated association between these SNPs and bipolar disorder [18]. A case-control study from Japan, failed to find significant correlations between *GNB1L* SNPs and schizophrenia, but did find lower levels of *GNB1L* in postmortem prefrontal cortex of schizophrenia patients compared to controls [19].

Our observation that *GNB1L* shows allele-specific differences in mRNA expression in the Han Chinese populations replicates a similar observation for *GNB1L* in the European population. Williams *et al.* [17] reported linear AEI ratios ranging from approximately -0.5 to +1.3 using a mSNP located in the 3'-untranslated region of *GNB1L* mRNA

(rs5746832; ratios calculated as A/G) compared with our observed range of linear AEI ratios of -0.45 to +1.17 obtained with an exon 7 mSNP (rs2073770; ratios calculated here as C/A for comparison). Williams *et al.* also obtained an asymmetric distribution of AEI ratios, although the degree of skewing was slightly less than what we observed. This difference may result from the use of different mSNPs and/or differences in linkage between *GNB1L* SNPs in European and Chinese populations. Consistent with the results of Williams *et al*, analysis of our genotyping data using Haploview [20] shows that the low-expression alleles of rs5746832 (*A*) and rs2073770 (*C*) on one hand and the high-expression alleles (*G*) and (*A*) on the other, reside within two distinct haplotypes in ~76% of the Han Chinese brain samples.

Analysis of genotyping results for our collection of brain samples also revealed that the *C*-allele of the mSNP rs2073770 is a preferentially associated with a haplotype containing the risk alleles for schizophrenia and bipolar disorder identified in the Han Chinese population by Li et al [18]. Our observation that the *C*-allele of rs2073770 is associated with low expression of *GNB1L* mRNA is consistent with the observation that hemizygous deletion of the VCF/DG critical region associates with elevated risk for schizophrenia and bipolar disorder.

3) *DISC1* (disrupted in schizophrenia; Ch1q42.1) comprises 13 exons spanning 415 kb on chromosome 1. Alternative splicing of the *DISC1* primary RNA transcript produces numerous protein isoforms that have been proposed to function as a protein scaffolds during neurodevelopment [21] and in the mature brain [21, 22]. Many genetic studies

have linked *DISC1* SNPs to serious psychiatric disorders, including schizophrenia, bipolar disorder, major depression and autism [21]. Genetic association studies in Han Chinese have produced mixed results, with weak association of a single *DISC1* SNP (rs2295959) with schizophrenia in females reported in one study [23] and robust association of two SNPs (rs821616 and rs821597) with schizophrenia in as second study [24]. A haplotype comprising the *C*-alleles of two SNPs (rs2738864 and rs16841582) was also reported to weakly associate with bipolar disorder [25]. A recent study also reported significant association for one *DISC1* SNP and several haplotypes with autism in the Han Chinese population [26].

A previous study of AEI of *DISC1* mRNA expression in human cerebral cortex found only one sample among 65 that produced an AEI ratio greater than 1.2 ($\log_2$AEI > 0.263) [27]. A data mining-based study of publicly available microarray expression data [28] and recent study using Illumina WG-6 Beadchips [29] reported evidence for the contribution of as many as 15 *cis*-acting SNPs to *DISC1* mRNA expression in human lymphocytes. By contrast, our mathematical modeling suggests that small AEI ratios are common among the Han Chinese brain samples and that two-to-three genetic variants, each unlinked to the mSNP, are sufficient to account for the observed distribution of ratios. Additional studies will be required to determine if any of the *cis*-acting SNPs identified in the microassay assays cited above contribute to the regulation of *DICS1* mRNA in human brain.

References

1. Wohrle FU, Daly RJ, Brummer T: **Function, regulation and pathological roles of the Gab/DOS docking proteins**. *Cell Commun Signal* 2009, **7**:22.

2. Coon KD, Myers AJ, Craig DW, Webster JA, Pearson JV, Lince DH, Zismann VL, Beach TG, Leung D, Bryden L *et al*: **A high-density whole-genome association study reveals that APOE is the major susceptibility gene for sporadic late-onset Alzheimer's disease**. *The Journal of clinical psychiatry* 2007, **68**(4):613-618.

3. Reiman EM, Webster JA, Myers AJ, Hardy J, Dunckley T, Zismann VL, Joshipura KD, Pearson JV, Hu-Lince D, Huentelman MJ *et al*: **GAB2 alleles modify Alzheimer's risk in APOE epsilon4 carriers**. *Neuron* 2007, **54**(5):713-720.

4. Bertram L, Tanzi RE: **Genome-wide association studies in Alzheimer's disease**. *Human molecular genetics* 2009, **18**(R2):R137-145.

5. Belbin O, Carrasquillo MM, Crump M, Culley OJ, Hunter TA, Ma L, Bisceglio G, Zou F, Allen M, Dickson DW *et al*: **Investigation of 15 of the top candidate genes for late-onset Alzheimer's disease**. *Human genetics* 2010, **129**(3):273-282.

6. Lin K, Tang M, Han H, Guo Y, Lin Y, Ma C: **GAB2 is not associated with late-onset Alzheimer's disease in Chinese Han**. *Neurol Sci* 2010, **31**(3):277-281.

7. Zhong XL, Yu JT, Hou GY, Xing YY, Jiang H, Li Y, Tan L: **Common variant in GAB2 is associated with late-onset Alzheimer's disease in Han Chinese**. *Clinica chimica acta; international journal of clinical chemistry* 2010, **412**(5-6):446-449.

8. Wang G, Pan XL, Cui PJ, Wang Y, Ma JF, Ren RJ, Deng YL, Xu W, Tang HD, Chen SD: **Association Study of the GAB2 Gene With the Risk of Alzheimer Disease in the Chinese Population**. *Alzheimer disease and associated disorders* 2011, *in press*.

9. Gong L, Liu M, Jen J, Yeh ET: **GNB1L, a gene deleted in the critical region for DiGeorge syndrome on 22q11, encodes a G-protein beta-subunit-like polypeptide**. *Biochimica et biophysica acta* 2000, **1494**(1-2):185-188.

10. Funke B, Pandita RK, Morrow BE: **Isolation and characterization of a novel gene containing WD40 repeats from the region deleted in velo-cardio-facial/DiGeorge syndrome on chromosome 22q11**. *Genomics* 2001, **73**(3):264-271.

11. Gothelf D, Frisch A, Michaelovsky E, Weizman A, Shprintzen RJ: **Velo-Cardio-Facial Syndrome**. *Journal of mental health research in intellectual disabilities* 2009, **2**(2):149-167.

12. Prasad SE, Howley S, Murphy KC: **Candidate genes and the behavioral phenotype in 22q11.2 deletion syndrome**. *Developmental disabilities research reviews* 2008, **14**(1):26-34.

13. Jolin EM, Weller RA, Weller EB: **Occurrence of affective disorders compared to other psychiatric disorders in children and adolescents with 22q11.2 deletion syndrome**. *Journal of affective disorders* 2011, in press.

14. Gothelf D, Presburger G, Zohar AH, Burg M, Nahmani A, Frydman M, Shohat M, Inbar D, Aviram-Goldring A, Yeshaya J *et al*: **Obsessive-compulsive disorder in**

patients with velocardiofacial (22q11 deletion) syndrome. *Am J Med Genet B Neuropsychiatr Genet* 2004, **126B**(1):99-105.

15. Murphy KC, Jones LA, Owen MJ: **High rates of schizophrenia in adults with velo-cardio-facial syndrome**. *Archives of general psychiatry* 1999, **56**(10):940-945.

16. Bassett AS, Chow EW, AbdelMalik P, Gheorghiu M, Husted J, Weksberg R: **The schizophrenia phenotype in 22q11 deletion syndrome**. *The American journal of psychiatry* 2003, **160**(9):1580-1586.

17. Williams NM, Glaser B, Norton N, Williams H, Pierce T, Moskvina V, Monks S, Del Favero J, Goossens D, Rujescu D *et al*: **Strong evidence that GNB1L is associated with schizophrenia**. *Human molecular genetics* 2008, **17**(4):555-566.

18. Li Y, Zhao Q, Wang T, Liu J, Li J, Li T, Zeng Z, Feng G, He L, Shi Y: **Association study between GNB1L and three major mental disorders in Chinese Han populations**. *Psychiatry research* 2011, **187**(3):457-459.

19. Ishiguro H, Koga M, Horiuchi Y, Noguchi E, Morikawa M, Suzuki Y, Arai M, Niizato K, Iritani S, Itokawa M *et al*: **Supportive evidence for reduced expression of GNB1L in schizophrenia**. *Schizophrenia bulletin* 2010, **36**(4):756-765.

20. Barrett JC, Fry B, Maller J, Daly MJ: **Haploview: analysis and visualization of LD and haplotype maps**. *Bioinformatics (Oxford, England)* 2005, **21**(2):263-265.

21. Chubb JE, Bradshaw NJ, Soares DC, Porteous DJ, Millar JK: **The DISC locus in psychiatric illness**. *Molecular psychiatry* 2008, **13**(1):36-64.

22. Kim JY, Duan X, Liu CY, Jang MH, Guo JU, Pow-anpongkul N, Kang E, Song H, Ming GL: **DISC1 regulates new neuron development in the adult brain via modulation of AKT-mTOR signaling through KIAA1212**. *Neuron* 2009, **63**(6):761-773.

23. Chen QY, Chen Q, Feng GY, Lindpaintner K, Wang LJ, Chen ZX, Gao ZS, Tang JS, Huang G, He L: **Case-control association study of Disrupted-in-Schizophrenia-1 (DISC1) gene and schizophrenia in the Chinese population**. *Journal of psychiatric research* 2007, **41**(5):428-434.

24. Qu M, Tang F, Yue W, Ruan Y, Lu T, Liu Z, Zhang H, Han Y, Zhang D, Wang F *et al*: **Positive association of the Disrupted-in-Schizophrenia-1 gene (DISC1) with schizophrenia in the Chinese Han population**. *Am J Med Genet B Neuropsychiatr Genet* 2007, **144B**(3):266-270.

25. Xiao Y, Zhang J, Wang Y, Wang P, Li X, Ji J, Yang F, Feng G, He L, He G: **Limited association between Disrupted in Schizophrenia 1 (DISC1) gene and bipolar disorder in the Chinese population**. *Psychiatric genetics* 2011, **21**(1):42-46.

26. Zheng F, Wang L, Jia M, Yue W, Ruan Y, Lu T, Liu J, Li J, Zhang D: **Evidence for association between Disrupted-in-schizophrenia 1 (DISC1) gene polymorphisms and autism in Chinese Han population: a family-based association study**. *Behav Brain Funct* 2011, **7**(1):14.

27. Hayesmoore JB, Bray NJ, Owen MJ, O'Donovan MC: **DISC1 mRNA expression is not influenced by common Cis-acting regulatory polymorphisms or imprinting**. *Am J Med Genet B Neuropsychiatr Genet* 2008, **147B**(7):1065-1069.

28. Hennah W, Porteous D: **The DISC1 pathway modulates expression of neurodevelopmental, synaptogenic and sensory perception genes**. *PloS one* 2009, **4**(3):e4906.

29. Carless MA, Glahn DC, Johnson MP, Curran JE, Bozaoglu K, Dyer TD, Winkler AM, Cole SA, Almasy L, Maccluer JW *et al*: **Impact of DISC1 variation on neuroanatomical and neurocognitive phenotypes**. *Molecular psychiatry* 2011, 1-9.
